# Polymerization, Stimuli‐induced Depolymerization, and Precipitation‐driven Macrocyclization in a Nitroaldol Reaction System**

**DOI:** 10.1002/chem.202201863

**Published:** 2022-09-19

**Authors:** Yunchuan Qi, Olof Ramström

**Affiliations:** ^1^ Department of Chemistry University of Massachusetts Lowell One University Ave. Lowell MA 01854 USA; ^2^ Department of Chemistry and Biomedical Sciences Linnaeus University SE-39182 Kalmar Sweden

**Keywords:** dynamic covalent, dynamer, nitroaldol, self-sorting, systems

## Abstract

Dynamic covalent polymers of different topology have been synthesized from an aromatic dialdehyde and α,ω‐dinitroalkanes via the nitroaldol reaction. All dinitroalkanes yielded dynamers with the dialdehyde, where the length of the dinitroalkane chain played a vital role in determining the structure of the final products. For longer dinitroalkanes, linear dynamers were produced, where the degree of polymerization reached a plateau at higher feed concentrations. In the reactions involving 1,4‐dinitrobutane and 1,5‐dinitropentane, specific macrocycles were formed through depolymerization of the linear chains, further driven by precipitation. At lower temperature, the same systemic self‐sorting effect was also observed for the 1,6‐dinitrohexane‐based dynamers. Moreover, the dynamers showed a clear adaptive behavior, displaying depolymerization and rearrangement of the dynamer chains in response to alternative building blocks as external stimuli.

## Graphical abstract

**Dynamic covalent** nitroaldol polymers of different topology is presented. Linear dynamers were generally produced, from which specific macrocycles were formed through depolymerization, driven by precipitation. The dynamers showed a clear adaptive behavior, displaying depolymerization and rearrangement of the dynamer chains in response to alternative building blocks as external stimuli.

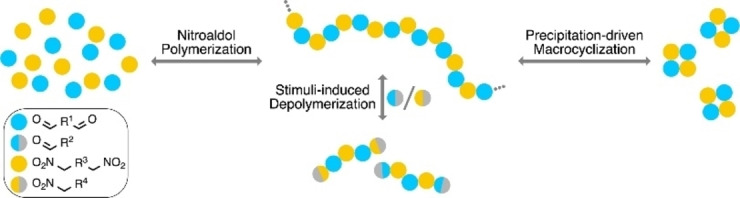

## Introduction

Complex entities constructed by reversible covalent bonds have demonstrated fascinating dynamic behaviors and rich functionalities.[^1^, ^2^, ^3^, ^4^, ^5^, ^6^, ^7^, ^8^, ^9^, ^10^, ^11^, ^12^, ^13^, ^14^, ^15^, ^16^, ^17^] For example, main‐chain dynamic covalent polymers (dynamers), in which the repeating units are connected by dynamic covalent bonds, show great potential in the development of novel functional materials for a wide range of applications. For example, dynamers show intriguing malleability, recyclability, self‐healing, stimuli‐responsiveness, shape‐memory and shear‐thinning effects, as well as tunable mechanical and optical properties.[^2^, ^4^, ^18^, ^19^, ^20^, ^21^, ^22^, ^23^] These materials are characterized by their adaptive properties, able to predictably respond to various stimuli, such as environmental factors (e. g., temperature, pH, light) and additional reagents, and can give rise to emergent properties.[^4^, ^11^, ^24^, ^25^, ^26^, ^27^]

A variety of dynamic covalent reactions have been applied to dynamer formation, such as imine formation (incl. hydrazones, oximes, etc.),[^28^, ^29^, ^30^, ^31^, ^32^] reversible cycloadditions (incl. olefin metathesis),[^1^, ^33^, ^34^, ^35^] acetals,^[36]^ transesterifications (incl. boronates),[^37^, ^38^, ^39^, ^40^, ^41^, ^42^, ^43^] and reactions involving disulfides.[^12^, ^44^] Among these, reactions that can generate carbon‐carbon bonds are of special interest since these transformations can be regarded as the essence of organic synthesis.[^45^, ^46^] Moreover, C−C bonds build up the carbon backbone of organic molecules and materials and constitute the foundation for further functionalization.[^47^, ^48^] Hence, employing reversible C−C bonds in molecules can potentially increase the dynamic performance of the whole entity since even the molecular skeleton is dynamic.^[3]^

Due to its efficiency in generating new C−C bonds, the nitroaldol (Henry) reaction has been widely applied in organic synthesis since it was first discovered in 1895.[^49^, ^50^, ^51^] The reaction is robust, stereogenic, self‐contained, and can be carried out under mild conditions. Studies have also shown its excellent performance in dynamic and systems chemistry,[^14^, ^48^, ^52^, ^53^, ^54^] and in the synthesis of dynamers under aqueous conditions.^[3]^ However, the scope and application of the nitroaldol reaction in dynamer synthesis still require a much‐needed expansion.

In our previous study,^[3]^ we exploited nitromethane as a bifunctional nitrocompound in dynamer synthesis, since it enables multiple nitroaldol reaction steps at the same carbon center.^[55]^ The degree of polymerization of these dynamers was limited (ca. 17), likely due to the steric hindrance and lower reactivity of the remaining α‐protons after the first nitroaldol reaction. To circumvent this limitation, we applied α,ω‐dinitroalkanes as building blocks in the present study, able to provide relatively equal reactivities at either end of the chains.

An important challenge with dynamers is the generation and control of different oligomeric and polymeric topologies, as well as their relative stimuli‐responsive properties. Since dynamer systems rely on multiple, sequential or parallel dynamic covalent reactions, the systems may undergo complex topology changes over time, potentially leading to self‐sorting of specific structures. Furthermore, such processes may lead to emergent property changes, and a detailed understanding of these processes is, therefore, of significant value for future applications.

**Figure 1:**
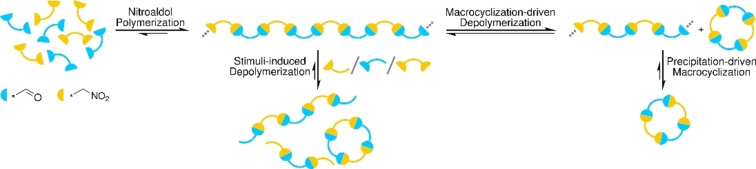
Nitroaldol dynamer formation, topology changes, self‐sorting, and stimuli‐responsiveness.

Herein, we report the generation and evaluation of complex nitroaldol systems involving a dialdehyde and a series of α,ω‐ dinitroalkanes. In all cases, dynamers were formed, and the interplay between different topologies were studied. The dynamerization process was evaluated with respect to dinitroalkane structure, catalyst loading, and feed concentration, and kinetic studies were performed. Intriguingly, specific macrocyclization was observed in several systems, occurring in competition with the formation of linear dynamers. This gave rise to polymerization/depolymerization processes, in several cases undergoing further self‐sorting through phase changes (Figure 1). Moreover, the stimuli‐responsive behavior of the dynamers was explored.

## Results and Discussion

**Figure 2:**
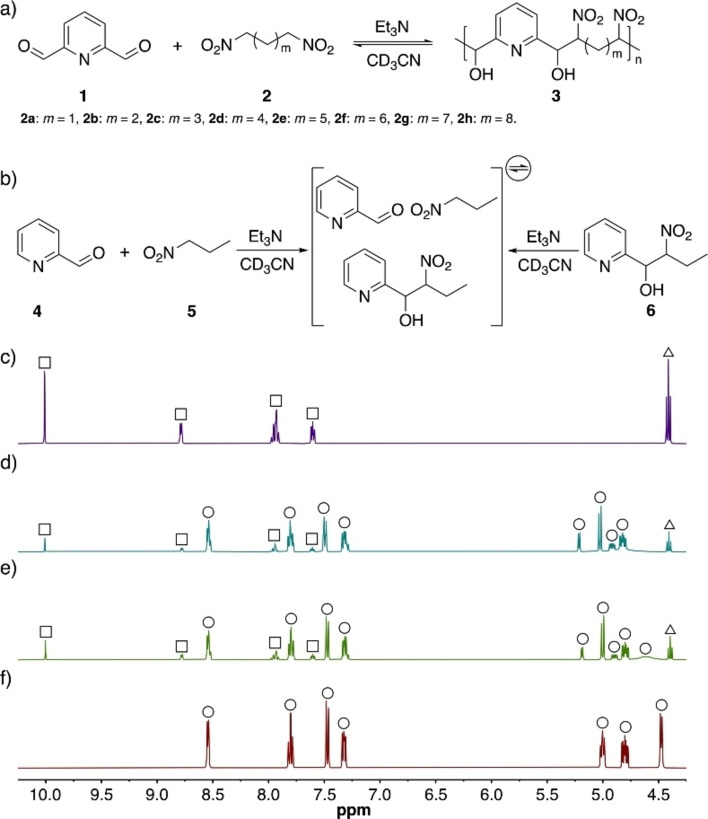
a) Proposed nitroaldol polymerization system; b) model nitroaldol system; ^1^H NMR spectra (400 MHz, CD_3_CN) of c) initial mixture of compounds **4** (□) and **5** (▵); d) mixture of compounds **4**, **5**, and Et_3_N after 24 h of incubation at r.t.; e) mixture of compound **6** (○) and Et_3_N after 24 h of incubation at r.t.; f) compound **6**.

Based on our previous report,^[3]^ 2,6‐pyridinedicarboxaldehyde (**1**) was chosen as the dialdehyde building block because of its high reactivity in the nitroaldol reaction. In complement to this, eight α,ω‐dinitroalkanes (**2**), including 1,3‐dinitropropane (**2a**), 1,4‐ dinitrobutane (**2b**), 1,5‐dinitropentane (**2c**), 1,6‐dinitrohexane (**2d**), 1,7‐dinitroheptane (**2e**), 1,8‐dinitrooctane (**2f**), 1,9‐dinitrononane (**2g**), and 1,10‐dinitrodecane (**2h**), were selected as dinitroalkane building blocks (Figure 2). Triethylamine (Et_3_N) was used as base throughout, and all reactions were carried out in acetonitrile due to the high solubility of the starting materials in this solvent. Under these conditions, dual entrypoint equilibration analysis indicated that the proposed nitroaldol polymerization operated under thermodynamic control (Figure 2).

**Figure 3:**
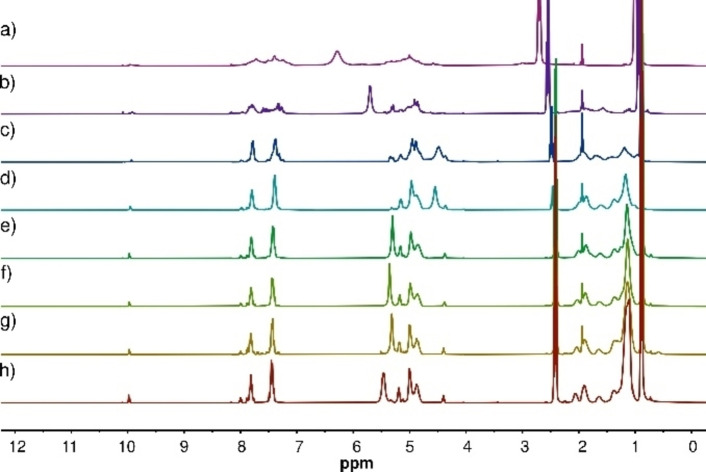
^1^H NMR spectra of a) **3a**; b) **3b**; c) **3c**; d) **3d**; e) **3e**; f) **3f**; g) **3g**; h) **3h** in solution (1.5 M feed concentration, 10 mol % Et_3_N; 400 MHz, CD_3_CN).

Polymers were generated from all systems within 48 h, as demonstrated by the broad signals in the ^1^H NMR spectra (Figure 3). Interestingly, white precipitates were formed in the systems containing the shorter dinitroalkanes **2b** and **2c**, whereas the solutions of the reactions with the other dinitroalkanes remained clear. Nevertheless, the formation of dynamers **3b** and **3c** resulted in spectra with typical nitroaldol characteristics, showing new nitroalcohol signals at 5.7–4.5 ppm. Dynamers **3d**–**3h**, having longer alkyl linkers, all produced relatively similar spectra apart from the hydroxyl protons shifts. High conversions of the starting materials were observed in all cases, and the resonances of the aldehyde protons (10.1 ppm) and the α‐protons of the nitroalkane (4.57–4.38 ppm) nearly disappeared.

The products of the polymerization process were analyzed by diffusion‐ordered NMR spectroscopy (DOSY NMR) and gel permeation chromatography (GPC). The DOSY experiments revealed the formation of species with diffusion coefficients in the range of (0.26–1.10)×10^−10^ cm^2^/s in all eight dynamer systems, indicating the formation of oligomers or polymers (Table 1, Figures S21–S28).^[56]^

**Table 1:** DOSY NMR and GPC data of dynamers **3a**–**3h** (1.5 M feed concentration).

Dynamer	*D* [cm^2^/s]	*M*_n_ [g/mol]	*M*_w_ [g/mol]	*M*_z_ [g/mol]	*Đ*	DP
**3a**	1.10×10^−10^	2700	3100	3600	1.1	10
**3b**	1.69×10^−10^	2700	2900	3200	1.1	9
**3c**	2.30×10^−10^	6300	8700	12400	1.4	21
**3d**	3.46×10^−11^	10400	17500	28000	1.7	33
**3e**	8.29×10^−11^	10500	18100	30200	1.7	32
**3f**	5.40×10^−11^	11800	20400	33300	1.7	35
**3g**	4.61×10^−11^	12100	20700	33100	1.7	34
**3h**	2.60×10^−11^	12000	20300	32500	1.7	34

*D*: diffusion coefficient; *Đ*: polydispersity index; DP: degree of polymerization.

Further information of the molecular weights and the weight distributions of the dynamers were provided by GPC (Table 1) from which clear trends could be discerned. In general, longer alkyl chains in the starting dinitroalkanes gave rise to higher molecular weights of the produced polymers. The degree of polymerization (DP) increased from dynamer **3a** to dynamer **3d** and reached a plateau at around 34 from dynamer **3d** to dynamer **3h**. The lower degree of polymerization for the shorter dinitroalkanes is likely an effect of steric hindrance (dynamer **3a**), possibly coupled with the precipitation of insoluble products (dynamers **3b**–**3c**). On the other hand, the comparable structure and reactivity of the longer dinitroalkanes (**2d**–**2h**), resulted in the generation of dynamers with very similar degrees of polymerization. Given the high solubility of the products, this is a reflection of the concentration‐dependent polymer growth and the dynamics of the polymerization reaction. At a concentration threshold of around 45 mM of the starting materials, the rates of polymerization and depolymerization were thus balanced. Moreover, dynamers **3a**–**3c** showed relatively low polydispersity indices (*Đ*), indicating the formation of shorter, more homogeneous oligomers; whereas dynamers **3d**–**3h** all had a wider molecular weight distribution (*Đ=*1.7).

**Figure 4:**
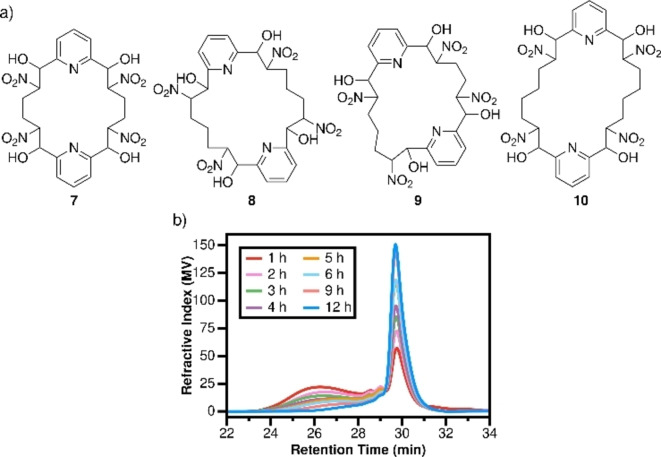
a) Structures of lowellane macrocycles **7**–**10**; b) GPC profile of the reaction between compounds **1** and **2b** from 1 h to 12 h.

Further NMR and MS analysis (Figures S37–S48) indicated that the precipitates in the systems involving dialdehyde **1** and dinitroalkane **2b**, as well as dialdehyde **1** with dinitroalkane **2c**, were composed of small molecules instead of polymers (Figure 4a). The HRMS results thus suggested the formation of structures composed of exactly two repeating units. Furthermore, while peaks corresponding to typical β‐nitroalcohols were recorded by NMR, neither aldehyde nor nitromethyl group signals were observed. These results indicate the formation of cyclic nitroalcohol structures where the two ends are connected, and the formed tetra‐ nitroalcohol macrocycles were coined *lowellanes* after the city where they were first synthesized. Macrocyclization in nitroaldol‐based systems is in this context of high interest, leading to cyclic, flexible carbon chains involving multiple stereocenters, in complement to previously observed cyclization in molybdenum‐catalyzed alkyne metathesis and imine exchange.[^30^, ^57^, ^58^]

To investigate the macrocyclization process in our system, dialdehyde **1** and dinitroalkane **2b** were dissolved in acetonitrile at a feed concentration of 0.5 M. After adding 10 mol % of Et_3_N, aliquots of the solution were withdrawn periodically and analyzed by GPC. The resulting GPC‐profile revealed that the reaction products were mainly short oligomers (*M*_n_ *=*3700, DP=6) in the early stage of the reaction. However, at longer reaction times, the oligomer concentration decreased while the amount of lowellane **7**, composed of two of each building block (DP corresponding to 2), increased (Figure 4b, Figures S49–S58). Thus, a depolymerization process promoted by macrocyclization took place over time, further driven by precipitation of the formed macrocycle. In general, macrocyclization coupled to linear polymerization indicates a ring‐chain polymerization process, observed with both supramolecular and covalent polymers,^[59]^ where macrocyclization is favored under high dilution conditions, and linear polymerization takes place at higher concentrations. However, the macrocyclization behavior in the present system suggests a special case in agreement with a ring‐closing depolymerization process.^[60]^ Thus, in this case, the rate of depolymerization is significantly lower than the rate of macrocyclization, thereby generating transient linear polymers that gradually degrade over time in response to macrocycle formation. Moreover, the dynamic system constituted a self‐sorting process, amplified by solubility constraints, and, although multiple macrocyclic species in principle could be formed, only lowellane **7** was formed.

At room temperature, the precipitation‐driven process was only observed with dinitroalkanes **2b** and **2c**. However, when the temperature was lowered to 8 °C, the macrocycle self‐sorting process was also observed with dinitroalkane **2d**. The process was in this case slower, and lowellane **10**, composed of two dialdehyde and two dinitrohexane building blocks, only precipitated from the polymerization solution after several weeks. Furthermore, the reaction of dialdehyde **1** with both dinitroalkanes **2b** and **2c** in the same mixture was carried out in acetonitrile. The reaction yielded a mixture of macrocycles, including lowellanes **7** and **8**, as well as hybrid lowellane **9**, all composed of two repeating units (Figure 4a). This further demonstrates the dynamic nature of the nitroaldol polymerization system. Unfortunately, the separation of the macrocyclic species from the crude product proved challenging, so compound **9** could only be observed by ESI‐MS (Figure S47).

**Figure 5:**
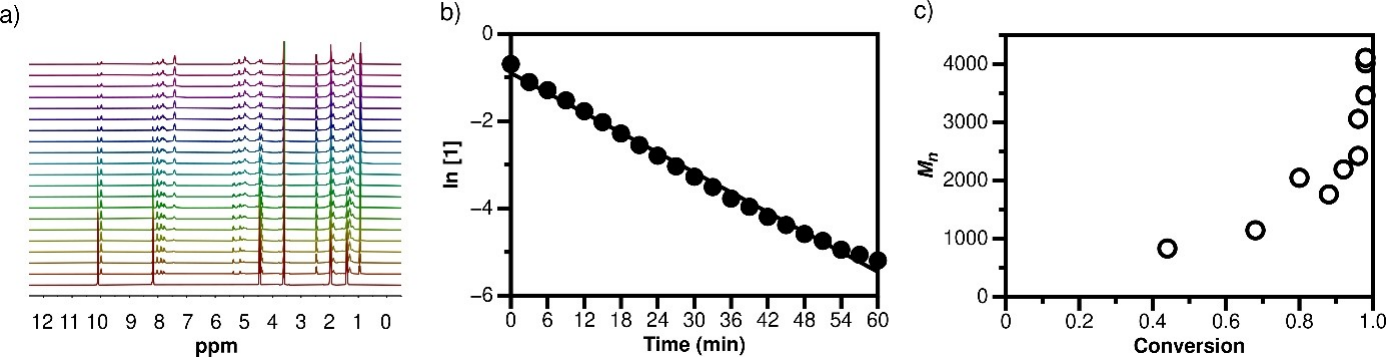
a) ^1^H NMR spectra of reaction between compounds **1** and **2d** in CD_3_CN at 0–60 min. (3 min interval; 0.5 M; 10 mol % Et_3_N; r.t.; 1,4‐dioxane as internal standard (*δ* 3.57 ppm); 400 MHz); b) Consumption of dialdehyde **1** following first‐order kinetics; c) *M*_n_ as function of conversion of compound **1**.

**Figure 6:**
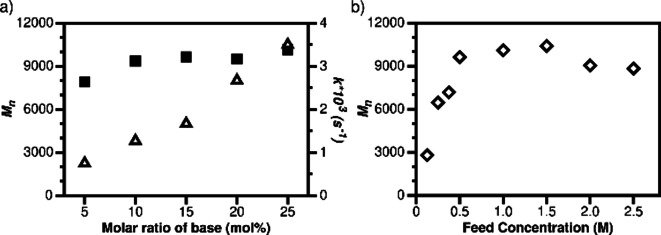
a) Rate constants of compound **1** in its reaction with compound **2d** with different amount of base (▵); *M*_n_ of polymers with different amount of base (▪). (0.5 M feed concentration); b) effect of feed concentration on *M*_n_.

To better understand the polymerization systems in which macrocyclization did not occur, a kinetic study was carried out using the reaction between dialdehyde **1** and dinitroalkane **2d** at room temperature in CD_3_CN. The ^1^H NMR spectra show that the reaction proceeded rapidly during the first hour after Et_3_N was added (Figure 5a), and then significantly slowed down (Figure S59). Using 1,4‐dioxane as the internal standard, the concentration change and conversion of compound **1** over time was calculated. The results reveal that the consumption of dialdehyde **1** matches first‐order kinetics during the first hour, with a rate coefficient (*k*) of 1.27×10^−3^/s (Figure 5b). Under these conditions, the relation between the number‐average molecular weight (*M_n_*) and the conversion of dialdehyde **1** from the GPC and ^1^H NMR data approximately fits a step‐growth polymerization model (Figure 5c, Figures S60–S69).^[61]^ The effect of the molar fraction of Et_3_N (5–25 mol % of compound **1**) on the reaction kinetics was also investigated by ^1^H NMR. The reaction was found to be approximately first order in Et_3_N, and the reaction rate constant increased with the amount of Et_3_N within the first hour (Figure 6a, ▵). After 12 h, when all reactions had reached equilibria, the final products showed almost identical ^1^H NMR spectra despite the difference in base percentage (Figure S75). On the other hand, the GPC results reveal clearer differences between the polymers (Table S4). The *M*_n_ of the polymer prepared using 5 mol % of Et_3_N was slightly lower than those made with higher ratios of base, although the difference was not significant in the range of 10–25 mol % (Figure 6a, ▪).

Dynamers are generally sensitive to the feed concentration of the starting materials, and this was next evaluated for the polymerization of compounds **1** and **2d**. In the concentration range of 0.125–2.5 M, all reactions resulted in polymer species with similar ^1^H NMR spectra (Figure S91), and DOSY analysis revealed a clear inverse dependency between the diffusion coefficients and the feed concentration (Table S5, Figures S92–S99). However, the GPC analysis resulted in a clearer influence of the feed concentration (Figure 6b, Table S6, Figures S100–S107). The molecular weights of the polymers increased steadily in the concentration range from 0.125 M to 0.5 M of the starting materials, reaching a plateau between 0.5 M to 1.5 M. This behavior is consistent with an isodesmic polymerization mechanism,^[59]^ as proposed for certain supramolecular polymers in which the interactions between the repeating units display equal association constants. However, at feed concentrations higher than 2.0 M, the molecular weights of the dynamers decreased to some extent, likely in response to viscosity effects and slower polymerization.[^62^, ^63^]

**Figure 7:**
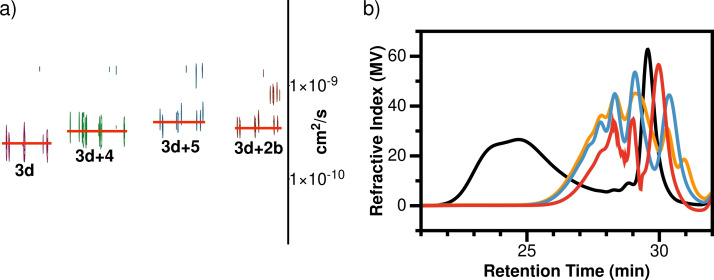
a) Pseudo‐2D DOSY plots of dynamer **3d** before and after reaction with compounds **4**, **5**, or **2b**; b) GPC profiles of dynamer **3d** in the absence (black) or presence of compounds **4** (blue), **5** (orange), or **2b** (red).

The responsive behavior of the polymers was investigated by adding monofunctional compounds (**4**, **5**) or bifunctional compound **2b** as stimuli. The results indicate that the dynamer underwent substantial changes in response to the added reagents. Thus, after exposure to the stimuli, the product solutions produced significantly higher diffusion coefficients than the original dynamer sample (Figure 7a, Table S7). Assuming that the viscosity of the solutions remains unchanged under these conditions, the results indicate that the polymer chains were shorter after responding to the stimuli.^[56]^ At the same time, the changes observed in the ^1^H NMR spectra were relatively minor since the functional groups are similar before and after the reaction (Figure S108).

However, as shown by GPC analysis (Figure 7b, Table S8), the molecular weight of the dynamer experienced significant drops after the stimuli exposures. The monofunctional compounds **4** and **5** induced changes that resulted in smaller polymer chains since they would act as polymerization stoppers. Compound **2b**, on the other hand, would favor macrocyclization over polymerization, which would lead to breakdown of the polymer. Even through no precipitate was observed by the naked eye in the reaction between compound 3d and dinitroalkane **2b**, the peak in the GPC profile at around 30 min retention time (Figure 7b, red trace) is consistent with the GPC profile of lowellane **7** (Figure S58). These results all demonstrate the depolymerization of the dynamer induced by different stimuli.

## Conclusion

In summary, a series of dynamic covalent polymers have been synthesized through the nitroaldol reaction, leading to structures with linear and intriguing cyclic topologies. The use of dinitroalkanes efficiently resulted in dynamers with higher degrees of polymerization than corresponding structures based on nitromethane,^[3]^ pronounced for building blocks with longer alkyl chains. A complex interplay between linear dynamers and macrocyclic oligomers were observed for shorter dinitroalkanes, where polymerization and depolymerization coupled with phase change of specific macrocycles occurred. This precipitation‐driven macrocyclization represents the first dynamic systemic self‐sorting process from a nitroaldol dynamer system. Furthermore, the adaptivity of the dynamers could be clearly shown, where the polymers responded to external stimuli through depolymerization and rearrangement of the polymer chains.

These results constitute important steps towards a detailed understanding of complex dynameric systems, and can lead to useful applications of nitroaldol‐based dynamers where the controlled reversible nature is of importance. Such applications can be found in many areas, ranging from 3D‐printing, surface adhesion, and degradable materials, to drug delivery, wound dressing, and matrices undergoing self‐healing. We also expect that the unique features of the dynamic nitroaldol polymers will enable them to find use in a wide variety of neighboring fields, such as systems chemistry, porous self‐assembled materials and frameworks, surface science, controlled adhesion technology, degradable and recyclable materials for temporary utility, etc. Some of these uses have great potential to become more widespread applications, as they can lead to “smart”, programmable, adaptive‐responsive materials.

## Experimental Section

Synthesis of dynamer **3** (typical procedure). 2,6‐Pyridinedicarboxaldehyde (**1**, 0.101 g, 0.75 mmol), α,ω‐dinitroalkane (0.75 mmol), and triethylamine (10.5 μL, 0.075 mmol) were dissolved in CD_3_CN (0.5 mL). The solution was kept at r.t. for 48 h without stirring and the resulting solution analyzed by NMR. The volatiles were then removed *in vacuo* and the residue was dissolved in THF for analysis by GPC.

Synthesis of lowellanes. The lowellanes were collected from the reaction between 2,6‐pyridinedicarboxaldehyde (**1**) and the corresponding α,ω‐dinitroalkane (**2b**, **2c**, **2d**) as a precipitate, washed several times by ethyl ether and dried under vacuum.

Dual entrypoint equilibration analysis. Performed using model system in deuterated acetonitrile in the presence of Et_3_N.^[64]^ From starting components: 2‐pyridinecarboxaldehyde (**4**) and 1‐nitropropane (**5**) were mixed in anhydrous CD_3_CN and stirred at rt under N_2_. Et_3_N (10 mol %) was added and the reaction was followed by NMR over time, indicating that equilibrium was reached within 24 h. From product: 2‐nitro‐1‐(pyridin‐2‐yl)butan‐1‐ol (**6**) was dissolved in anhydrous CD_3_CN and stirred at r.t. under N_2_. Et_3_N (10 mol %) was added and the reaction progress followed. The same equilibrium as from the starting components was reached within 24 h.

## Conflict of interest

The authors declare no conflict of interest.

1

## Supporting information

As a service to our authors and readers, this journal provides supporting information supplied by the authors. Such materials are peer reviewed and may be re‐organized for online delivery, but are not copy‐edited or typeset. Technical support issues arising from supporting information (other than missing files) should be addressed to the authors.

Supporting Information

## Data Availability

The data that support the findings of this study are available in the supplementary material of this article.
