# Supplementary material for: Polymerization, Stimuli‐induced Depolymerization, and Precipitation‐driven Macrocyclization in a Nitroaldol Reaction System
Source: Chemistry. 2022 Sep 19;28(64):e202201863. doi: 10.1002/chem.202201863 (PMC9826525; doi:10.1002/chem.202201863)
Supplement: Supplementary file 1 — Supporting Information [file CHEM-28-0-s001.pdf]

Chemistry–A European Journal

Supporting Information

Polymerization, Stimuli-induced Depolymerization, and Precipitation-driven Macrocyclization in a Nitroaldol Reaction System

Yunchuan Qi and Olof Ramström*

Contents

General methods and materials S1
Synthesis S1
NMR spectra of components S3
DOSY NMR and GPC data of dynamers S12
NMR, MS, and GPC data of lowellane macrocycles S19
Reaction kinetics S28
Effects of base S33
Effects of feed concentration S41
System dynamics - stimuli-responsiveness S49
References S50

General methods and materials

All reagents and solvents were obtained from commercial suppliers and used without further purification. ^{1}H NMR, ^{13}C NMR and diffusion-ordered spectroscopy (DOSY) NMR data were recorded on a JEOL ECZ 400 spectrometer at 298 K. Chemical shifts are reported as δ-values (ppm) with (residual) solvent as internal reference. *J*-values are given in hertz (Hz). All NMR data was processed using MestReNova 14.2.1. 2D DOSY plots were generated by Bayesian transformation. Gel permeation chromatography (GPC) tests were performed on a Waters Alliance 2695 GPC with THF as the mobile phase. GPC data was processed using the Empower Pro software. High-resolution mass spectroscopy (HRMS) data were obtained from a Bruker SolariX FT-ICR Mass Spectrometer (ESI FT-ICR). Thin layer chromatography (TLC) was performed on aluminum plates coated with silica gel 60 F254 (0.25 mm, Merck), visualized with UV-detection. Flash column chromatography was performed on silica gel 60, 0.032-0.063 mm (Alfa Aesar).

Synthesis

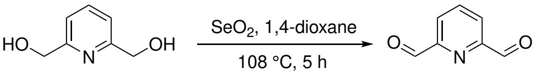

1

2,6-Pyridinedicarboxaldehyde (1).[1] 2,6-Pyridinedimethanol (1.39 g, 10 mmol) and SeO_2 (1.11 g, 10 mmol) was added to 1,4-dioxane (30 mL). The reaction mixture was refluxed with stirring at 108 °C for 5 h. After the reaction, the solid residue was filtered off, and the solvent in the filtrate was removed under reduced pressure. The crude product was purified by column chromatography (SiO_2, Hexanes:EtOAc 3:1 v/v). The product was a white crystal with the yield of 87%. ^{1}H NMR (400 MHz, DMSO-d_6) δ 10.09 (s, 2H), 8.30 (dd, *J* = 8.4, 6.9 Hz, 1H), 8.20 (d, *J* = 7.7 Hz, 2H). ^{13}C NMR (101 MHz, DMSO-d_6) δ 192.8, 152.6, 139.6, 125.8.

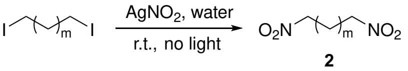

2

2a: *m* = 1, **2b**: *m* = 2, **2c**: *m* = 3, **2d**: *m* = 4, **2e**: *m* = 5, **2f**: *m* = 6, **2g**: *m* = 7, **2h**: *m* = 8.

α,ω-Dinitroalkanes (2a–2h).[2] The parent α,ω-diiodoalkane (10 mmol) and $AgNO_2$ (6.16 g, 40 mmol) was added into deionized water (40 mL). The mixture was stirred overnight under room temperature with the exclusion of light. After the reaction, residual solid was filtered, and ethyl acetate extracted the solution. The crude product was purified by column chromatography (SiO_2, Hexanes:EtOAc 4:1 v/v). The products were colorless liquids or solids with the yields around 40%.

1,3-Dinitropropane (2a). ^{1}H NMR (400 MHz, $CDCl_3$) δ 4.57 (t, *J* = 6.4 Hz, 4H), 2.67 (p, *J* = 6.4 Hz, 2H). ^{13}C NMR (101 MHz, $CDCl_3$) δ 71.6, 24.4.

1,4-Dinitrobutane (2b). ^{1}H NMR (400 MHz, $CDCl_3$) δ 4.55 – 4.39 (m, 4H), 2.20 – 2.06 (m, 4H). ^{13}C NMR (101 MHz, $CDCl_3$) δ 74.4, 24.1.

1,5-Dinitropentane (2c). ^{1}H NMR (400 MHz, $CDCl_3$) δ 4.40 (t, *J* = 6.8 Hz, 4H), 2.12 – 2.01 (m, 4H), 1.56 – 1.44 (m, 2H). ^{13}C NMR (101 MHz, $CDCl_3$) δ 75.0, 26.5, 23.2.

1,6-Dinitrohexane (2d). ^{1}H NMR (400 MHz, $CDCl_3$) δ 4.38 (t, *J* = 6.9 Hz, 4H), 2.01 (t, *J* = 7.0 Hz, 4H),

1.44 (p, J = 3.7 Hz, 4H). ^{13}C NMR (101 MHz, $CDCl_3$) δ 75.4, 27.0, 25.7.

1,7-Dinitroheptane (2e). ^{1}H NMR (400 MHz, $CDCl_3$) δ 4.38 (t, J = 7.0 Hz, 4H), 2.01 (p, J = 6.7 Hz, 4H), 1.44 – 1.37 (m, 6H). ^{13}C NMR (101 MHz, $CDCl_3$) δ 75.6, 28.2, 27.2, 26.0.

1,8-Dinitrooctane (2f). ^{1}H NMR (400 MHz, $CDCl_3$) δ 4.38 (t, J = 7.0 Hz, 4H), 2.00 (t, J = 7.0 Hz, 4H), 1.44 – 1.32 (m, 8H). ^{13}C NMR (101 MHz, $CDCl_3$) δ 75.7, 28.6, 27.4, 26.2.

1,9-Dinitrononane (2g). ^{1}H NMR (400 MHz, $CDCl_3$) δ 4.38 (t, J = 7.0 Hz, 4H), 2.00 (p, J = 7.0 Hz, 4H), 1.43 – 1.25 (m, 10H). ^{13}C NMR (101 MHz, $CDCl_3$) δ 75.8, 29.0, 28.7, 27.4, 26.2.

1,10-Dinitrodecane (2h). ^{1}H NMR (400 MHz, $CDCl_3$) δ 4.38 (t, J = 7.0 Hz, 4H), 2.00 (p, J = 7.0 Hz, 4H), 1.42 – 1.23 (m, 12H). ^{13}C NMR (101 MHz, $CDCl_3$) δ 75.8, 29.2, 28.9, 27, 26.3.

<u>CAUTION</u>: α,ω-Dinitroalkanes are potentially explosive. Store them in a cool safety cabinet for flammables. Keep them away from hot surfaces, flames, and sparks. Use with caution.

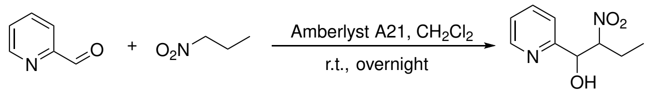

4 **5** **6**

2-Nitro-1-(pyridin-2-yl)butan-1-ol (6).[3] 2-Pyridinecarboxaldehyde (0.19 mL, 2 mmol), 1-nitropropane (0.2 mL, 2.2 mmol), and Amberlyst A21 resin (0.4 g) were mixed in CH_2Cl_2 (5 mL). The mixture was stirred overnight, after which the resin was filtered off. The solvent was removed under reduced pressure and the crude product was purified by column chromatography (SiO_2, Hexanes:EtOAc 1:1 v/v), yielding the product as a light-yellow dense oil (21%). ^{1}H NMR (400 MHz, CD_3CN) δ 8.59 (dt, J = 4.9, 1.3 Hz, 1H), 7.85 (td, J = 7.7, 1.8 Hz, 1H), 72 (d, J = 7.8 Hz, 1H), 7.37 (ddd, J = 7.7, 4.8, 1.2 Hz, 1H), 5.05 (dd, J = 7.9, 6.2 Hz, 1H), 4.85 (ddd, J = 10.5, 7.9, 3.8 Hz, 1H), 4.52 (d, J = 6.2 Hz, 1H), 1.99 – 1.89 (m, 1H), 1.56 (dqd, J = 14.9, 7.4, 3.8 Hz, 1H), 0.89 (t, J = 7.4 Hz, 3H). ^{13}C NMR (101 MHz, CD_3CN) δ 159.0, 150.0, 138.1, 124.5, 123.2, 95.5, 75.6, 24.2, 10.3. HRMS (ESI FT-ICR, m/z): $[M+Na]^+$ calcd. for $C_9H_{12}N_2O_3Na^+$: 219.0740; found: 219.0736.

3,6,10,13-tetranitro-1,8(2,6)-dipyridinacyclotetradecaphane-2,7,9,14-tetraol (lowellane 7). Lowellane **7** was collected from the reaction between compounds **1** and **2b** as a precipitate, washed several times by ethyl ether and dried under vacuum. ^{1}H NMR (400 MHz, DMSO-d_6) δ 8.00 – 7.75 (m, 2H), 76 – 7.31 (m, 4H), 6.46 – 6.27 (m, 1H), 6.06 – 5.78 (m, 2H), 5.53 – 4.96 (m, 6H), 4.90 – 4.72 (m, 3H), 2.06 – 0.79 (m, 8H). ^{13}C NMR (101 MHz, DMSO-d_6) δ 160.6, 158.6, 158.5, 157.6, 138.9, 124.3, 123.4, 120.3, 92.8, 92.5, 92.3, 91.8, 91.6, 90.7, 90.5, 75.5, 75.0, 75.0, 74.9, 26.7, 20.9. HRMS (ESI FT-ICR, m/z): $[M+Na]^+$ calcd. for $C_{22}H_{26}N_6O_{12}Na^+$: 589.1501; found: 589.1493.

3,7,11,15-tetranitro-1,9(2,6)-dipyridinacyclohexadecaphane-2,8,10,16-tetraol (lowellane 8). Lowellane **8** was collected from the reaction between compounds **1** and **2c** as a precipitate, washed several times by ethyl ether and dried *in vacuo*. ^{1}H NMR (400 MHz, DMSO-d_6) δ 7.90 – 7.75 (m, 2H), 72 – 7.28 (m, 4H), 6.46 – 6.03 (m, 4H), 5.38 – 4.61 (m, 8H), 2.06 – 0.58 (m, 12H). ^{13}C NMR (101 MHz, DMSO-d_6) δ 158.8, 139.0, 122.6, 93.5, 75.9, 29.0, 22.6. HRMS (ESI FT-ICR, m/z): $[M+Na]^+$ calcd. for $C_{24}H_{30}N_6O_{12}Na^+$: 617.1814; found: 617.1788.

3,6,10,14-tetranitro-1,8(2,6)-dipyridinacyclopentadecaphane-2,7,9,15-tetraol (lowellane 9). 2,6-Pyridinedicarboxaldehyde (0.0675 g, 0.5 mmol), 1,4-dinitrobutane (0.037 g, 0.25 mmol), 1,5-dinitropentane (0.0405 g, 0.25 mmol), and triethylamine (7 µL, 0.05 mmol) were dissolved in CH_3CN (0.5 mL). The solution was kept at rt for 48 h without stirring. The resulting precipitate was collected by filtration, washed by ethyl ether, and dried *in vacuo*. Lowellanes **7**, **8**, and **9** were observed by ESI-MS. HRMS (ESI FT-ICR, m/z): $[M+Na]^+$ calcd. for $C_{23}H_{28}N_6O_{12}Na^+$: 603.1660; found: 603.1660.

3,8,12,17-tetranitro-1,10(2,6)-dipyridinacyclooctadecaphane-2,9,11,18-tetraol (lowellane 10). 2,6-Pyridinedicarboxaldehyde (0.101 g, 0.5 mmol), 1,6-dinitrohexane (0.132 g, 0.75 mmol), and triethylamine (10.5 µL, 0.075 mmol) were dissolved in CH_3CN (0.5 mL). The solution was kept at 8 ºC for >1 month without stirring. The resulting precipitate was collected by filtration, washed by ethyl ether, and dried *in vacuo*. ^{1}H NMR (400 MHz, DMSO-d_6) δ 7.87 (q, J 8.2 Hz, 2H), 7.44 (t, J 7.9 Hz, 4H), 6.43 – 6.29 (m, 2H), 6.29 – 6.09 (m, 2H), 5.26 – 4.72 (m, 8H), 1.76 (dt, J 26.2, 15.7 Hz, 4H),

1.50 – 0.77 (m, 12H). ^{13}C NMR (101 MHz, DMSO-d_6) δ 159.1, 158.9, 138.8, 123.2, 120.4, 93.0, 92.6, 91.5, 75.5, 74.8, 30.2, 29.5, 25.9. HRMS (ESI FT-ICR, m/z): $[M+Na]^+$ calcd. for: $C_{26}H_{34}N_6O_{12}Na^+$: 645.2118; found: 645.2118.

NMR spectra of components

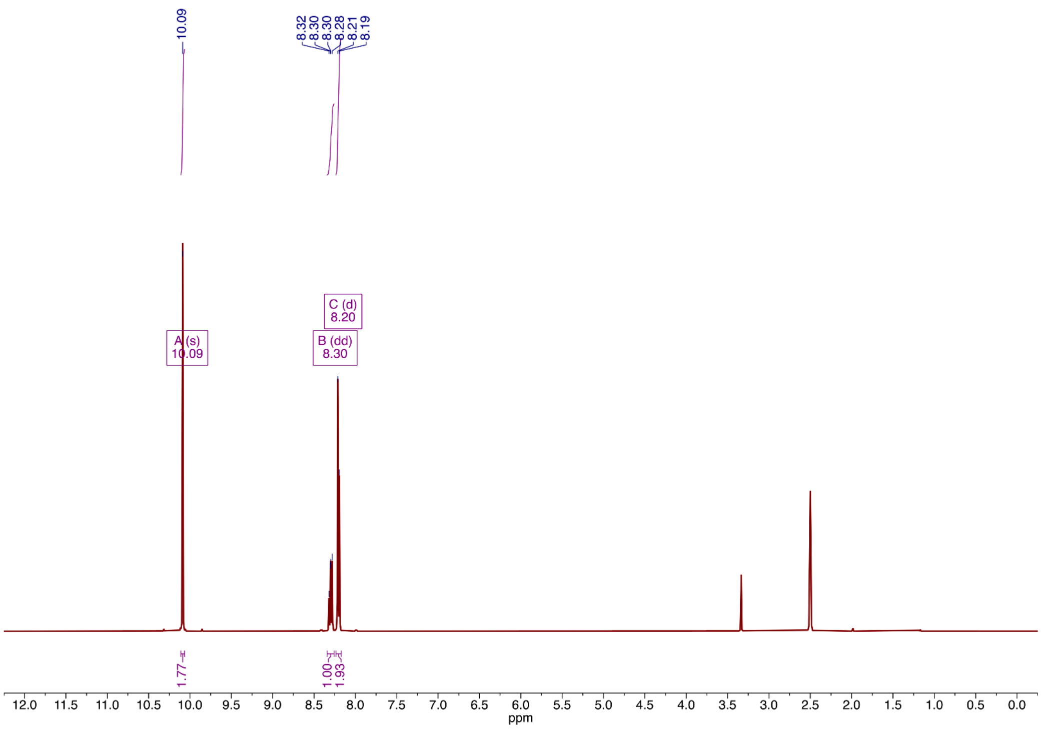

Figure S1: *^1H NMR spectrum of compound **1** in DMSO-d_6.*

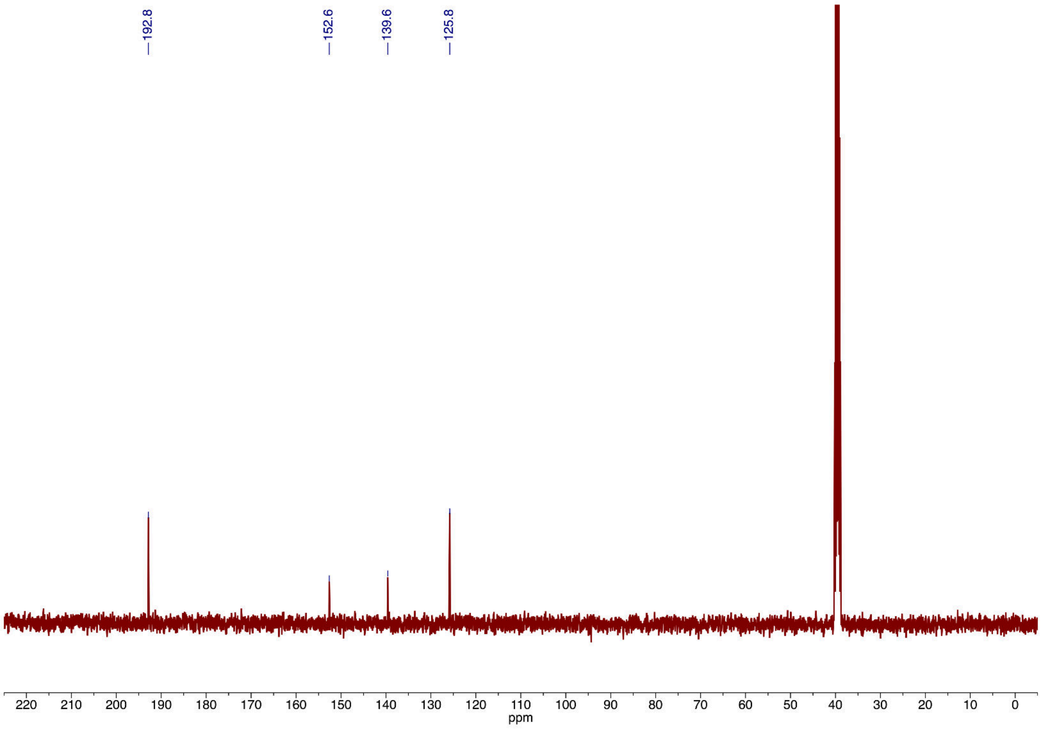

Figure S2: *^{13}C NMR spectrum of compound **1** in DMSO-d_6.*

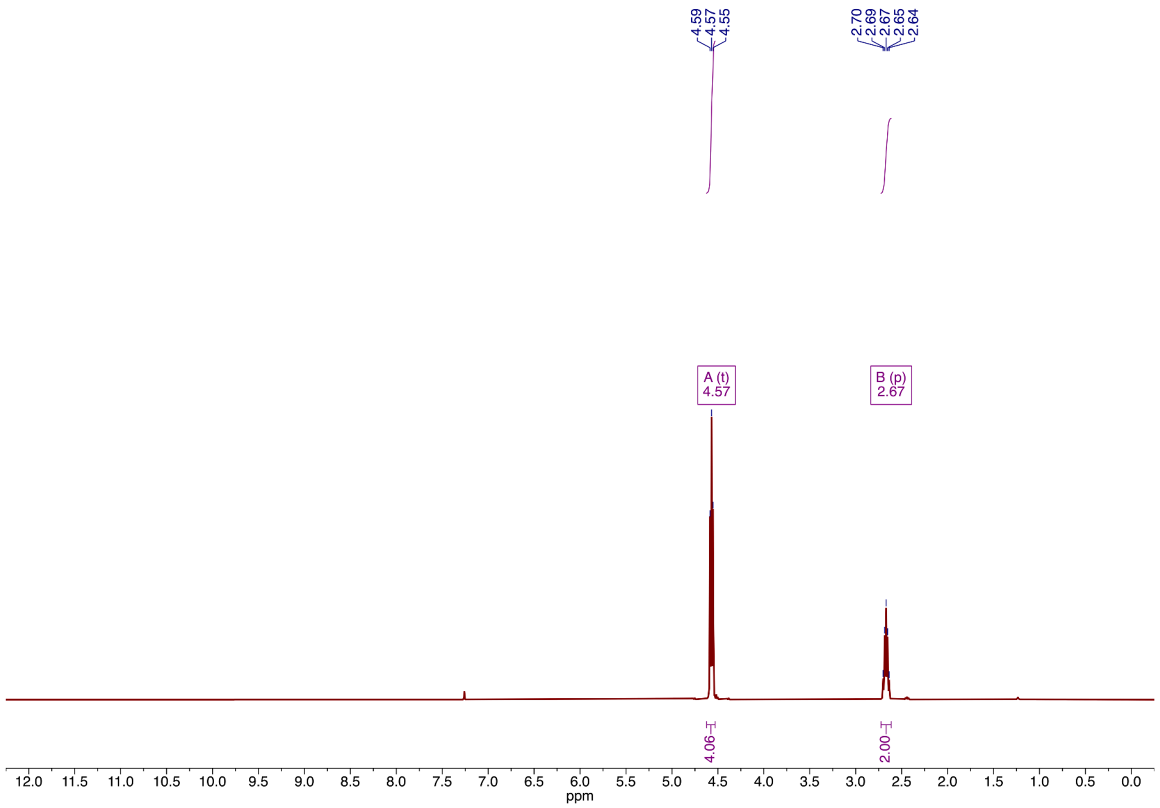

Figure S3: *^{1}H NMR spectrum of compound **2a** in $CDCl_3$.*

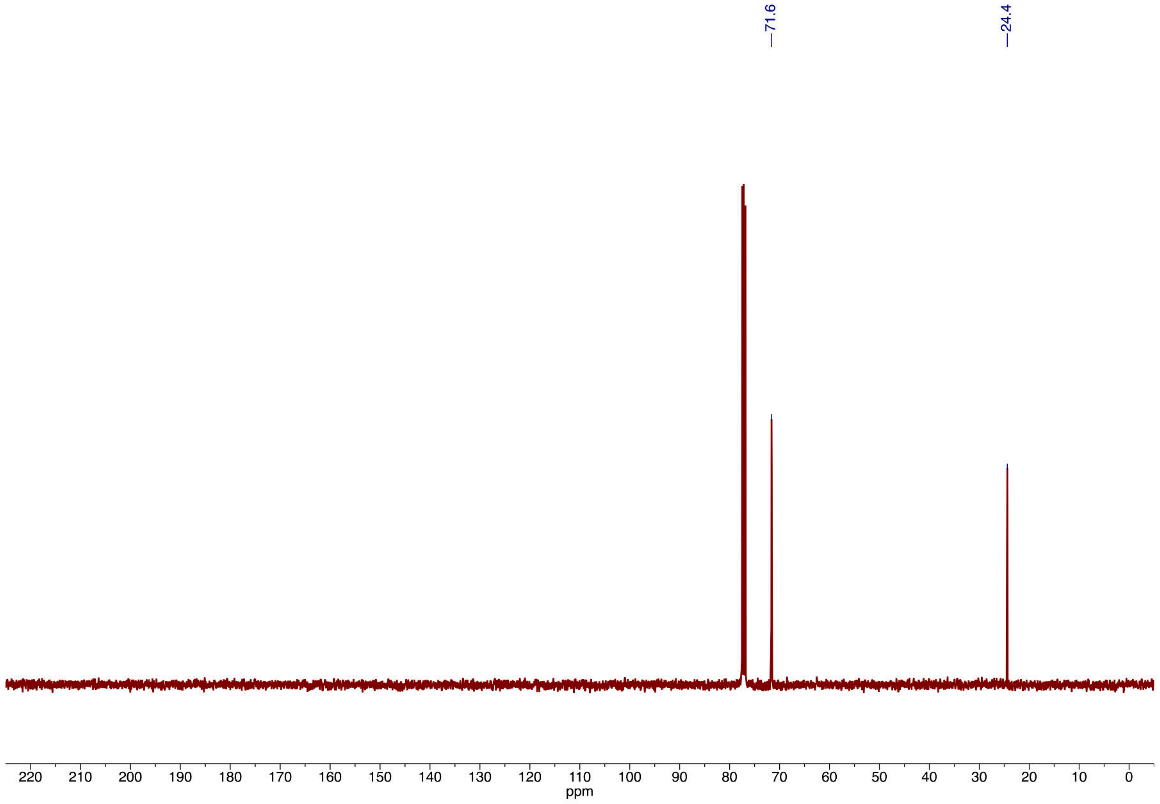

Figure S4: *^{13}C NMR spectrum of compound **2a** in $CDCl_3$.*

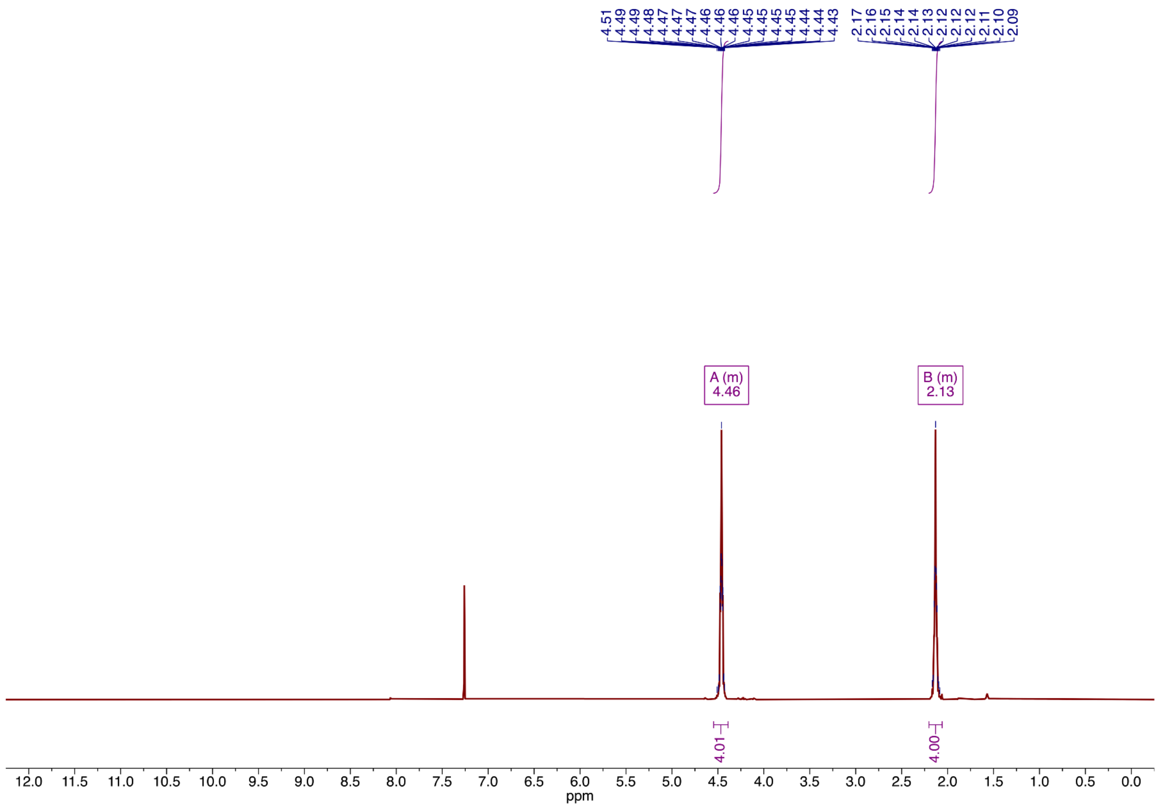

Figure S5: *^{1}H NMR spectrum of compound **2b** in $CDCl_3$.*

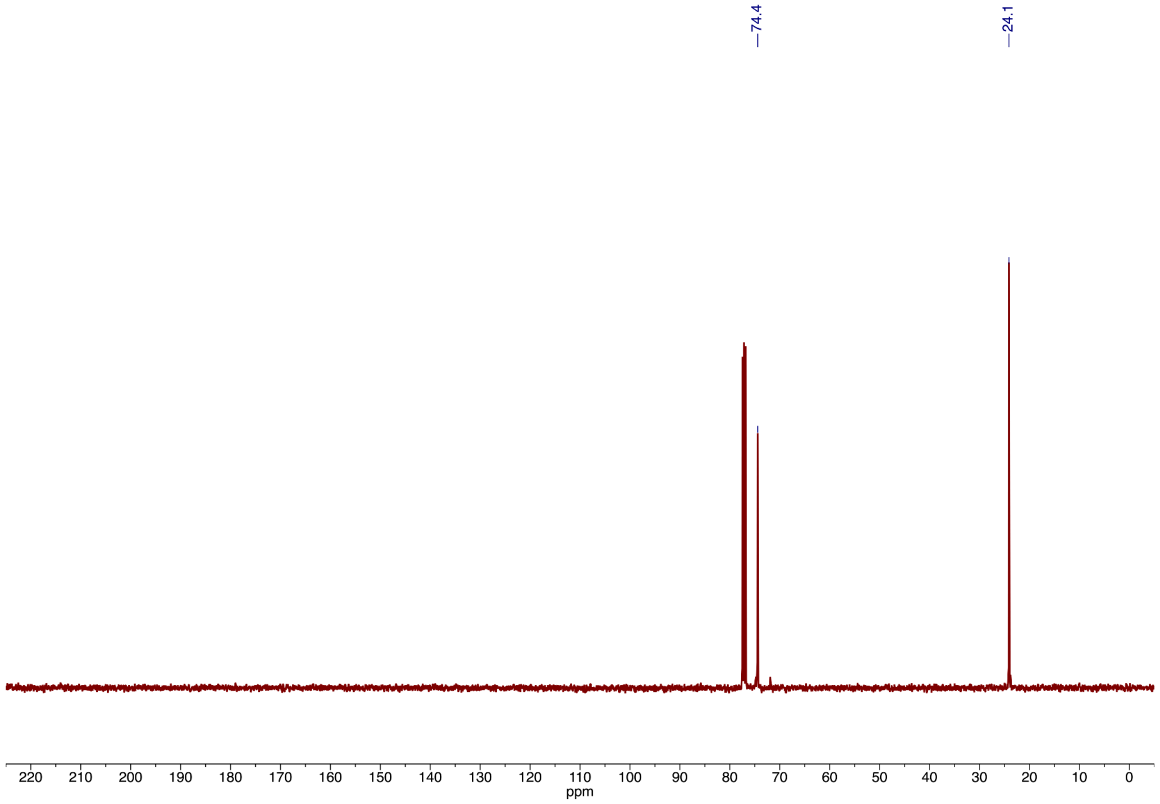

Figure S6: *^{13}C NMR spectrum of compound **2b** in $CDCl_3$.*

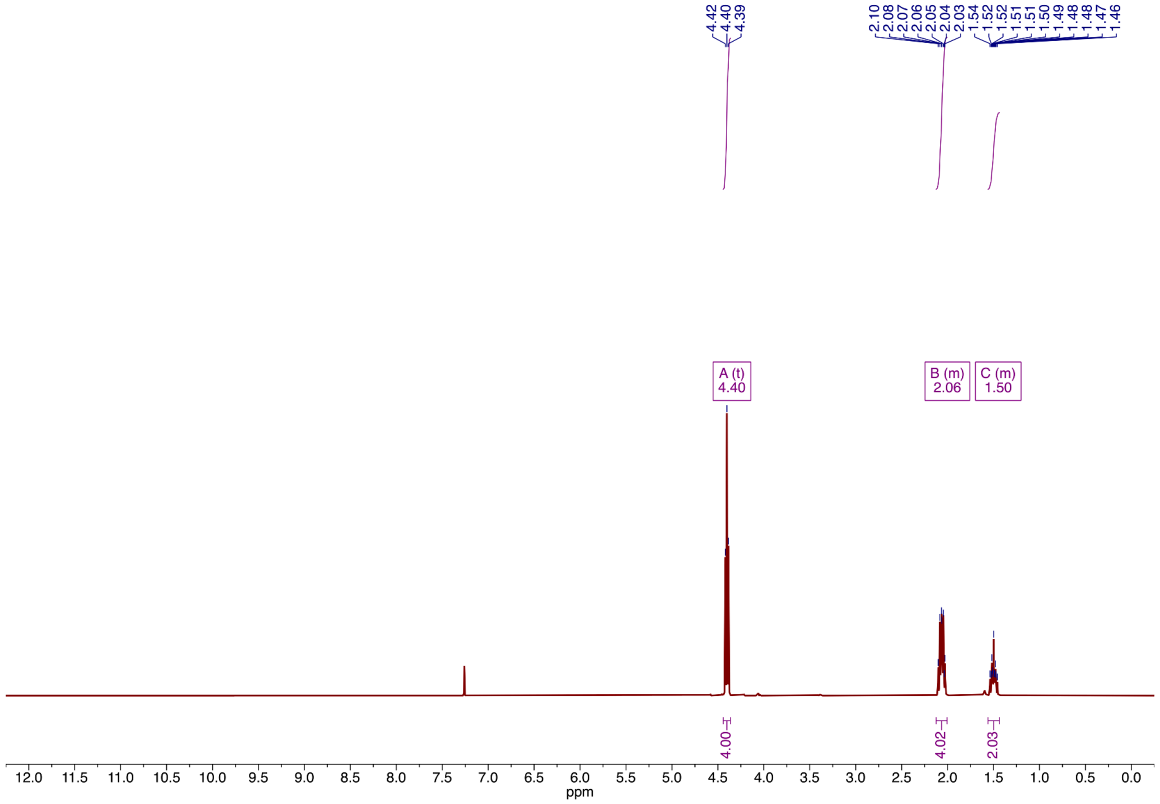

Figure S7: *^{1}H NMR spectrum of compound **2c** in $CDCl_3$.*

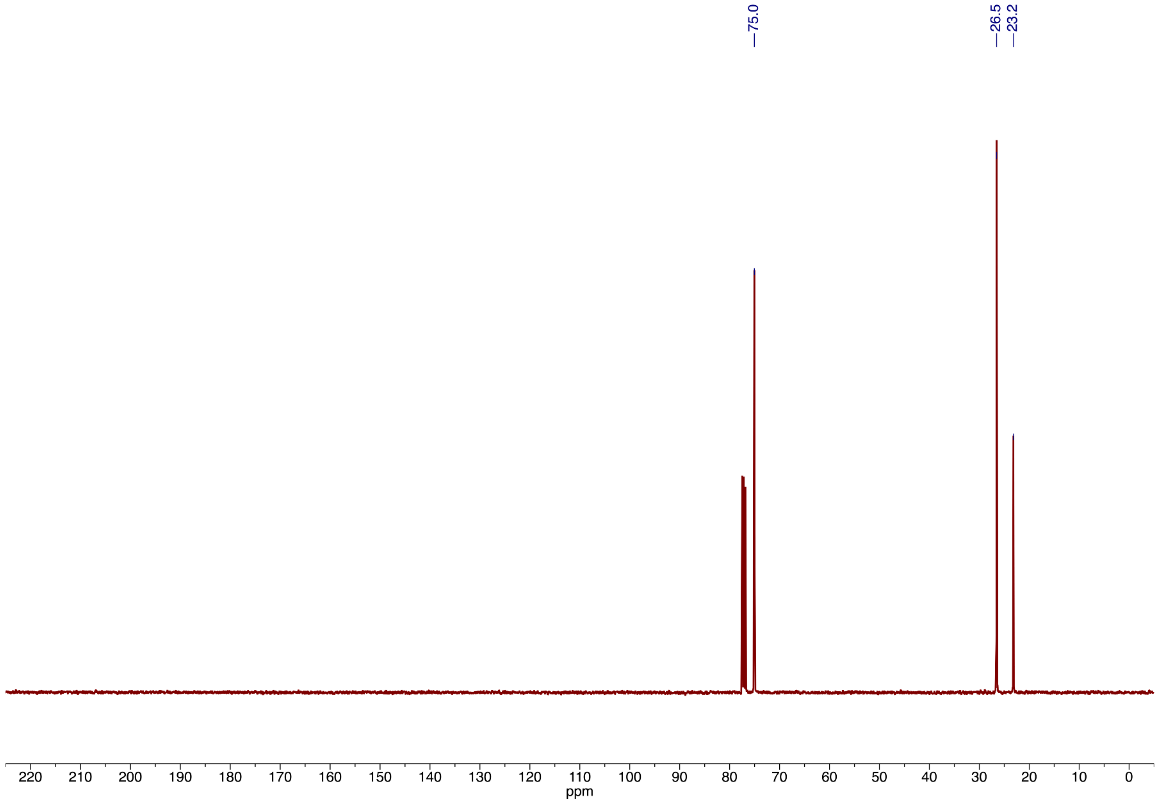

Figure S8: *^{13}C NMR spectrum of compound **2c** in $CDCl_3$.*

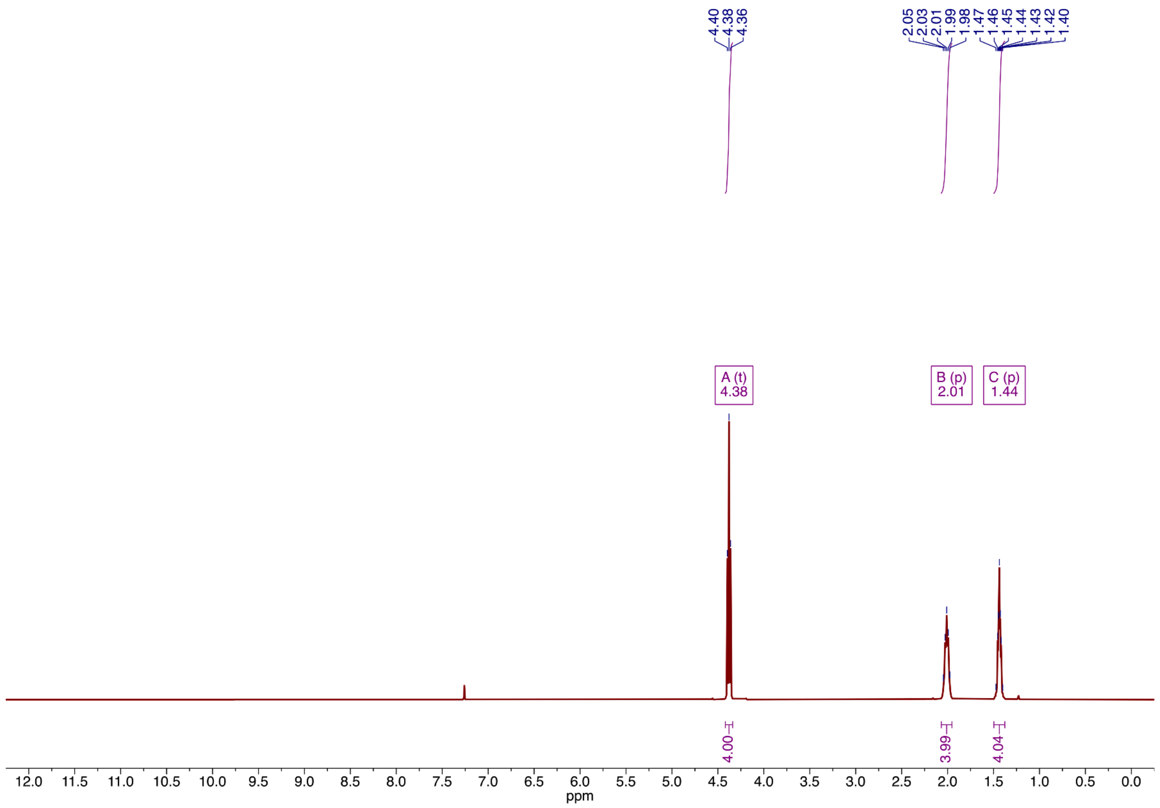

Figure S9: *^{1}H NMR spectrum of compound **2d** in $CDCl_3$.*

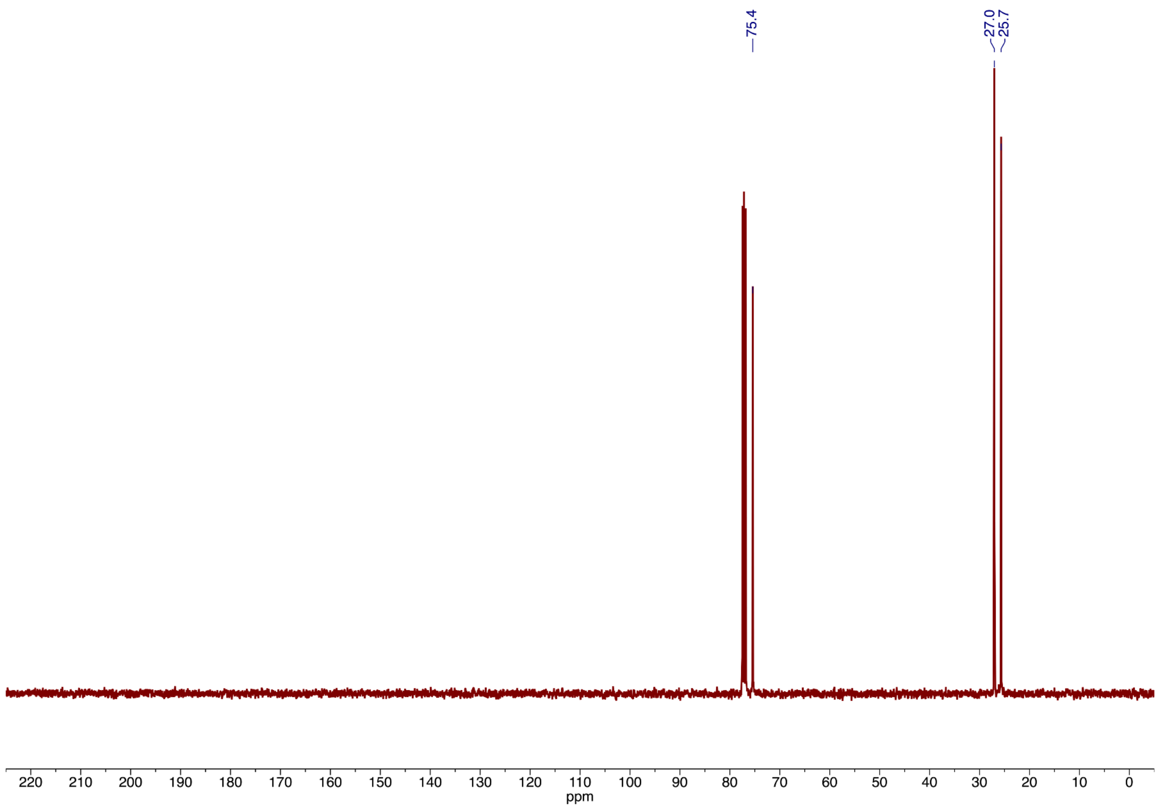

Figure S10: *^{13}C NMR spectrum of compound **2d** in $CDCl_3$.*

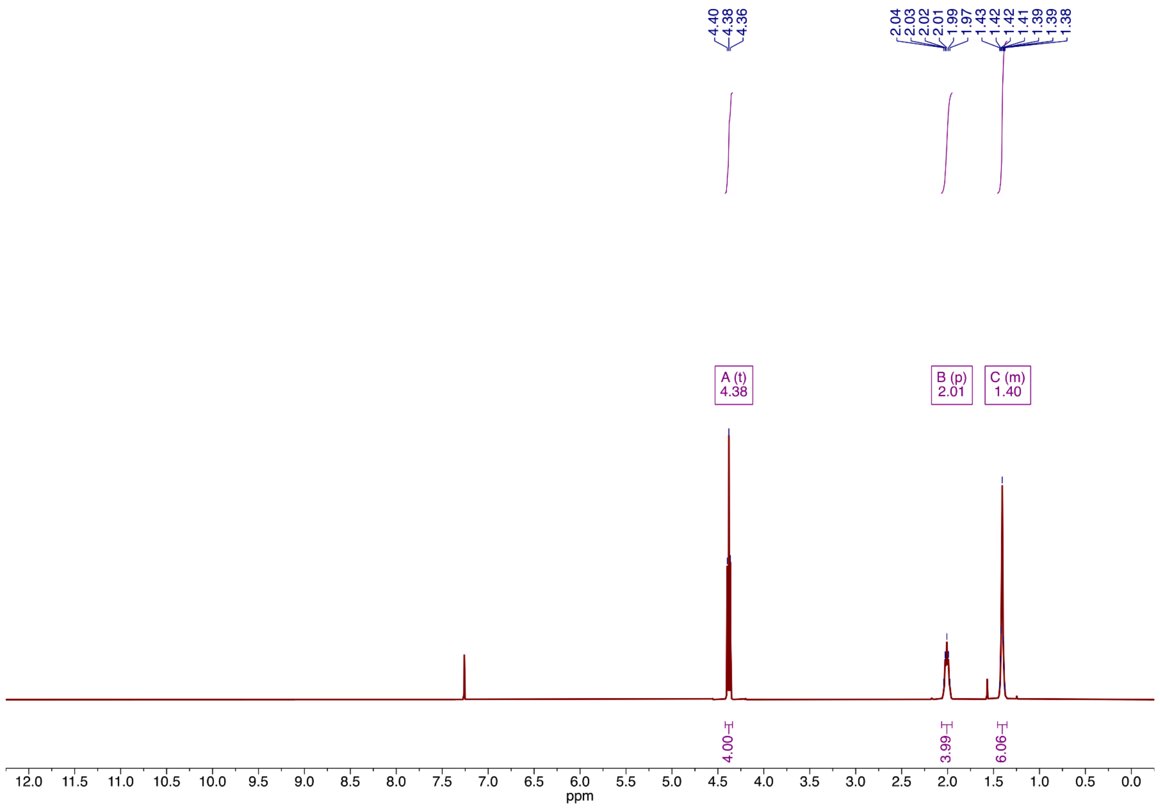

Figure S11: *¹H NMR spectrum of compound* ***2e*** *in $CDCl_3$.*

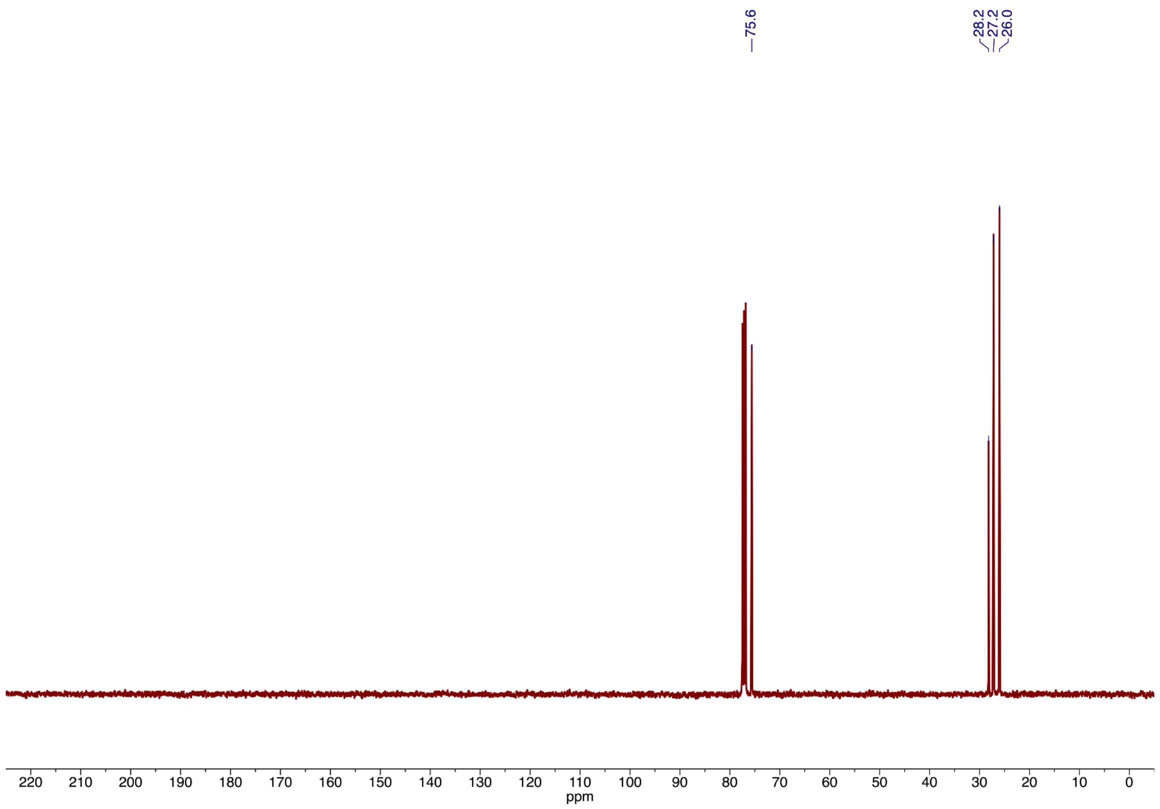

Figure S12: *¹³C NMR spectrum of compound* ***2e*** *in $CDCl_3$.*

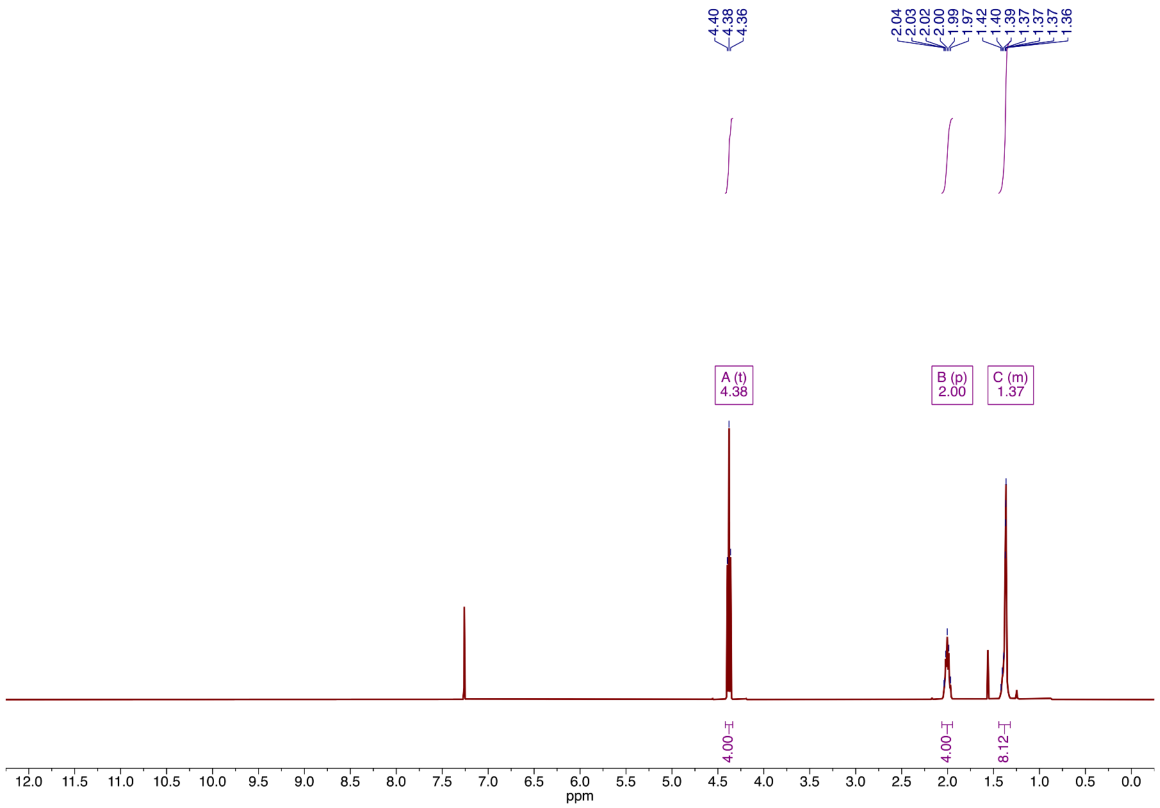

Figure S13: *¹H NMR spectrum of compound* ***2f*** *in* $CDCl_3$.

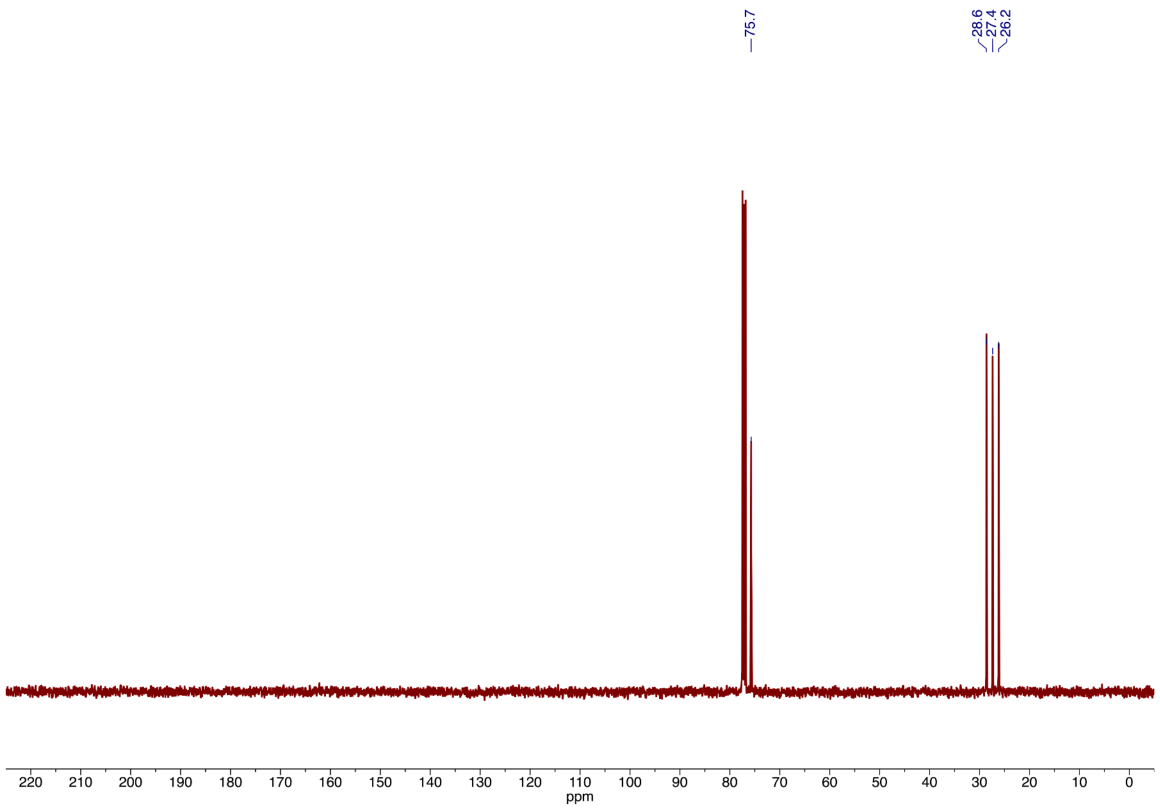

Figure S14: *¹³C NMR spectrum of compound* ***2f*** *in* $CDCl_3$.

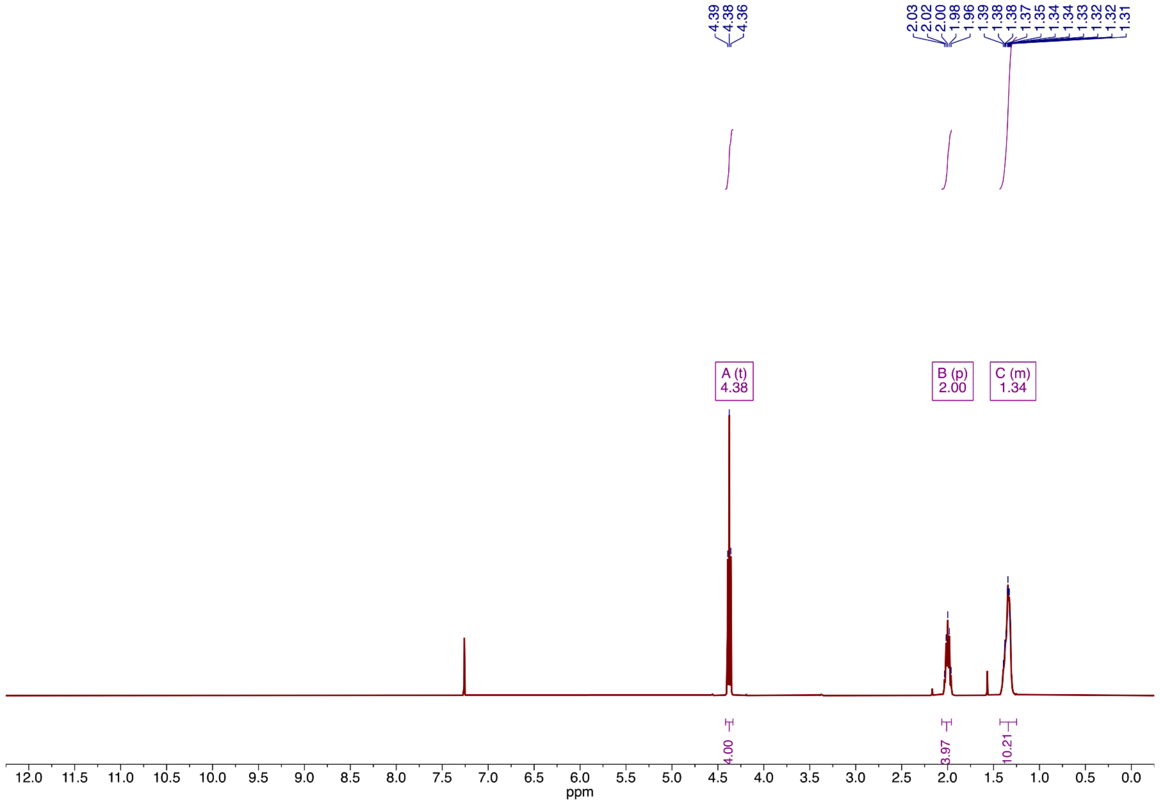

Figure S15: *^{1}H NMR spectrum of compound **2g** in $CDCl_3$.*

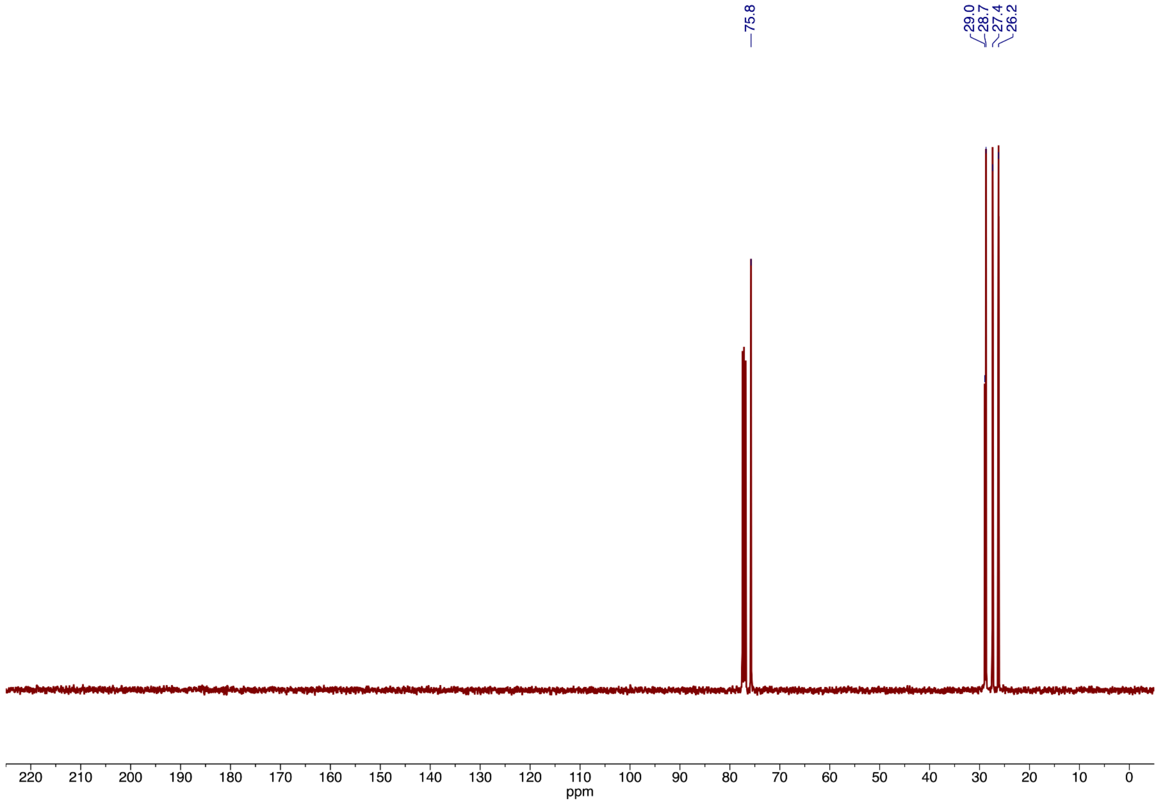

Figure S16: *^{13}C NMR spectrum of compound **2g** in $CDCl_3$.*

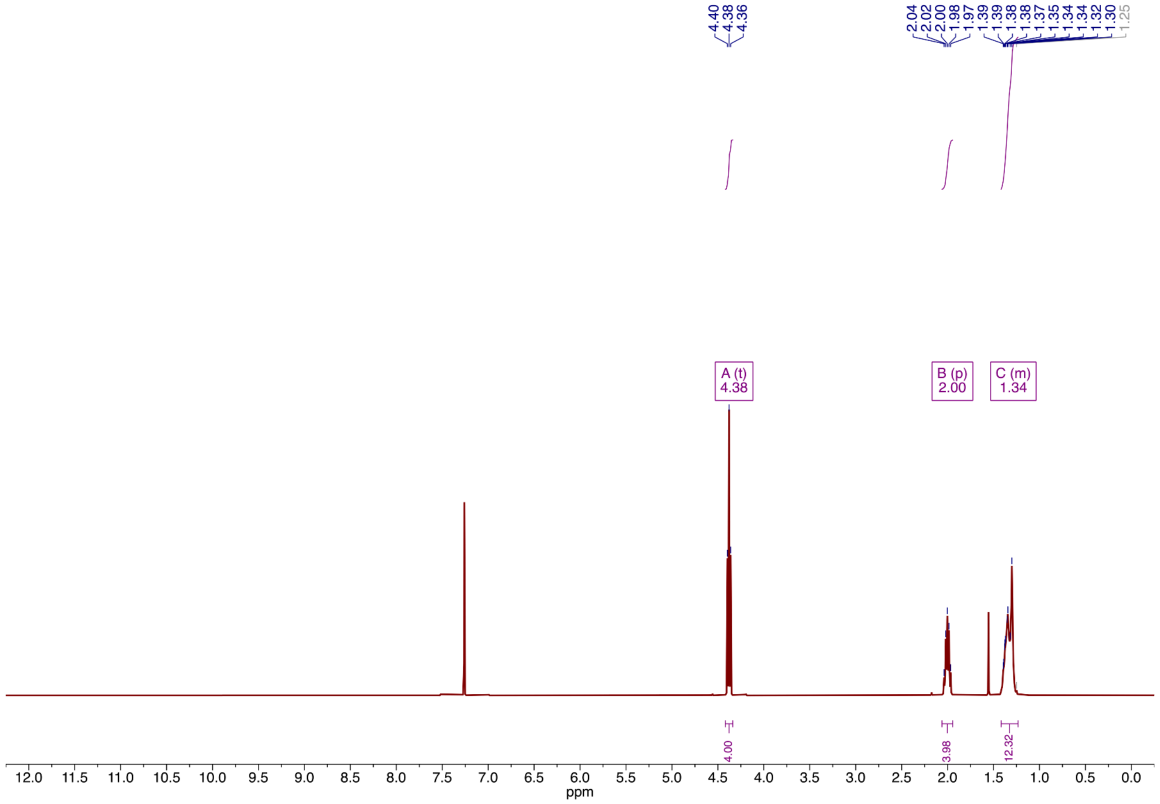

Figure S17: *^{1}H NMR spectrum of compound* ***2h*** *in $CDCl_3$.*

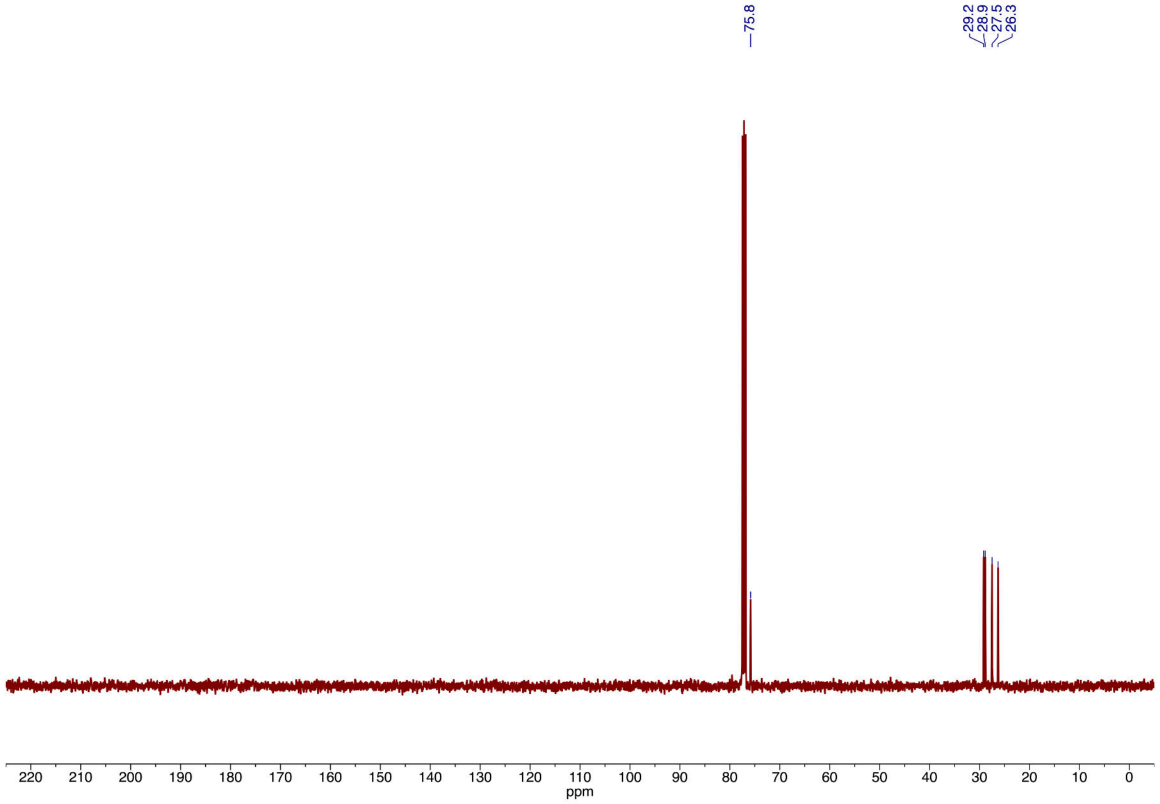

Figure S18: *^{13}C NMR spectrum of compound* ***2h*** *in $CDCl_3$.*

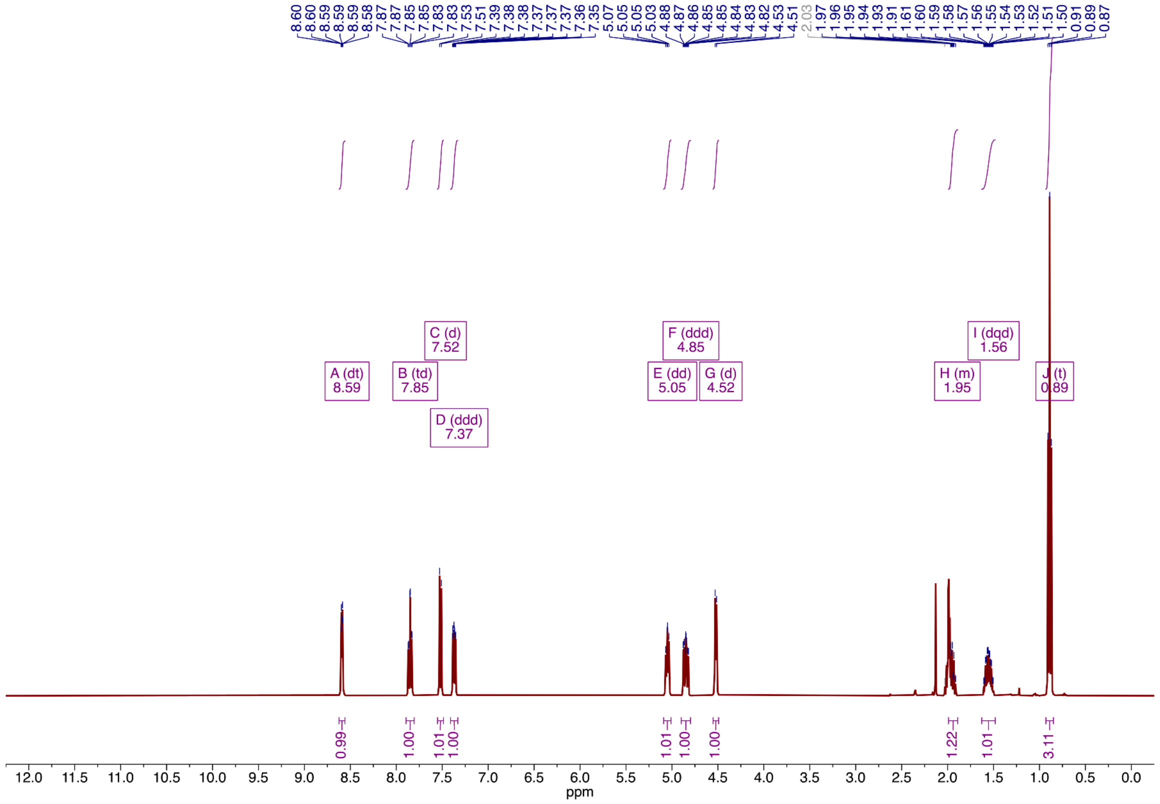

Figure S19: *^{1}H NMR spectrum of compound **6** in CD_3CN.*

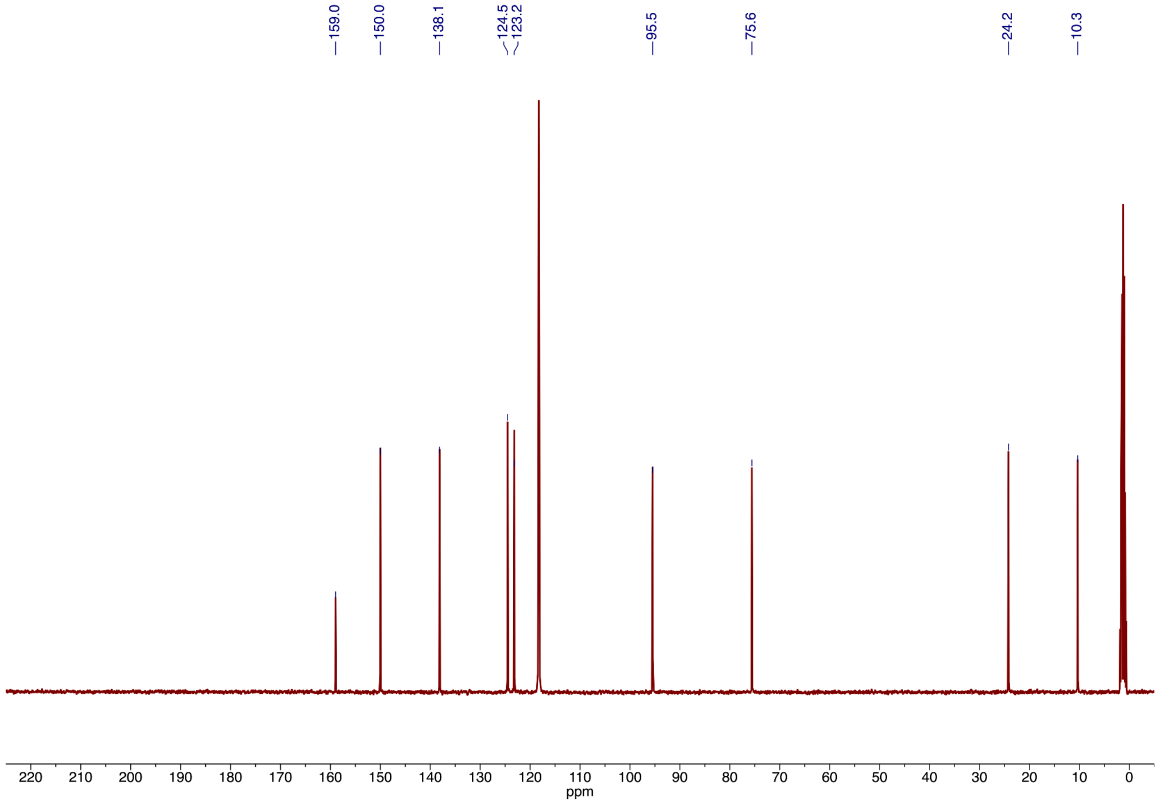

Figure S20: *^{13}C NMR spectrum of compound **6** in CD_3CN.*

DOSY NMR and GPC data of dynamers

NMR spectra

All dynamer samples were prepared from compounds **1** and **2** in CD_3CN (1.5 M; 10 mol% Et_3N).

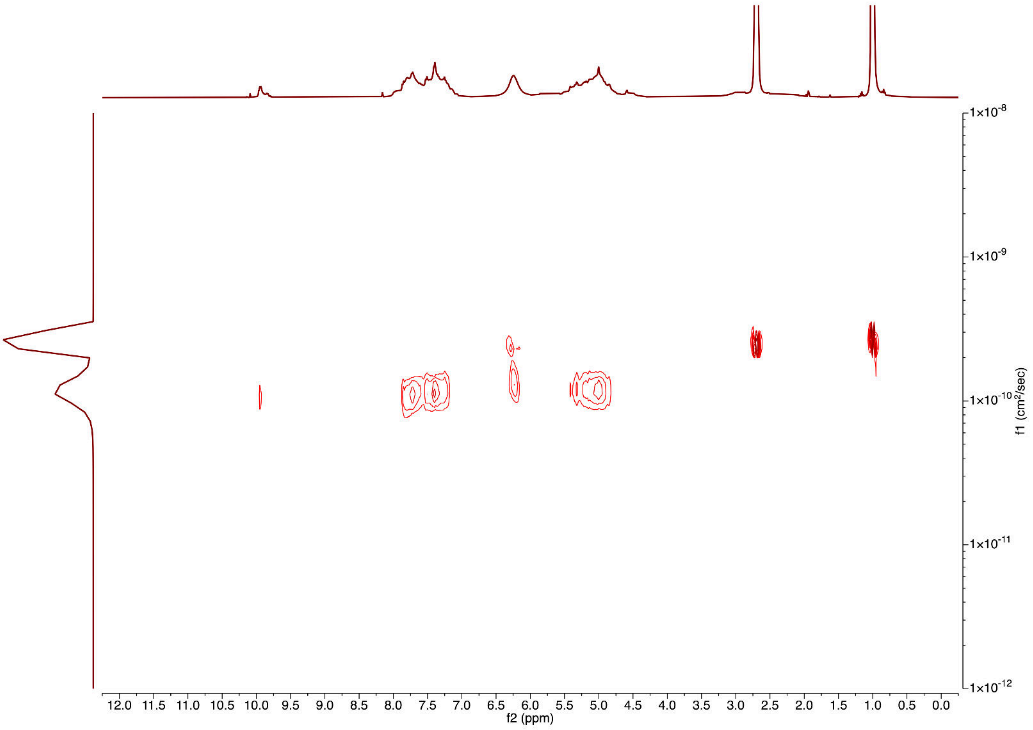

Figure S21: *Pseudo-2D DOSY plot of dynamer **3a** (1.5 M, CD_3CN).*

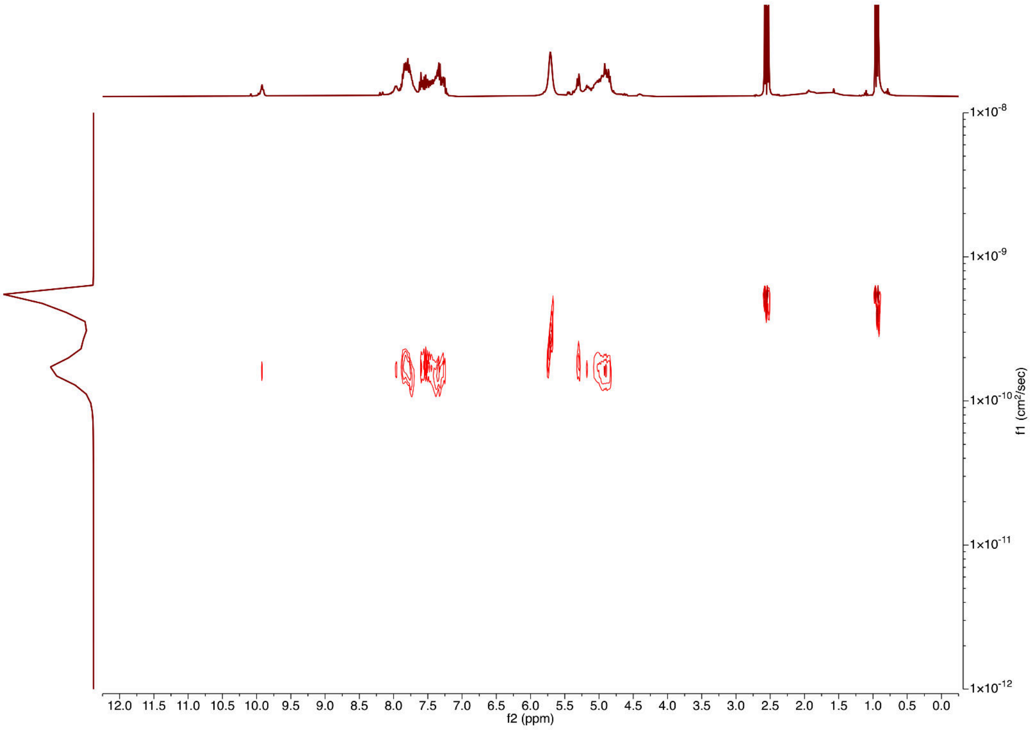

Figure S22: *Pseudo-2D DOSY plot of dynamer **3b** (1.5 M, CD_3CN).*

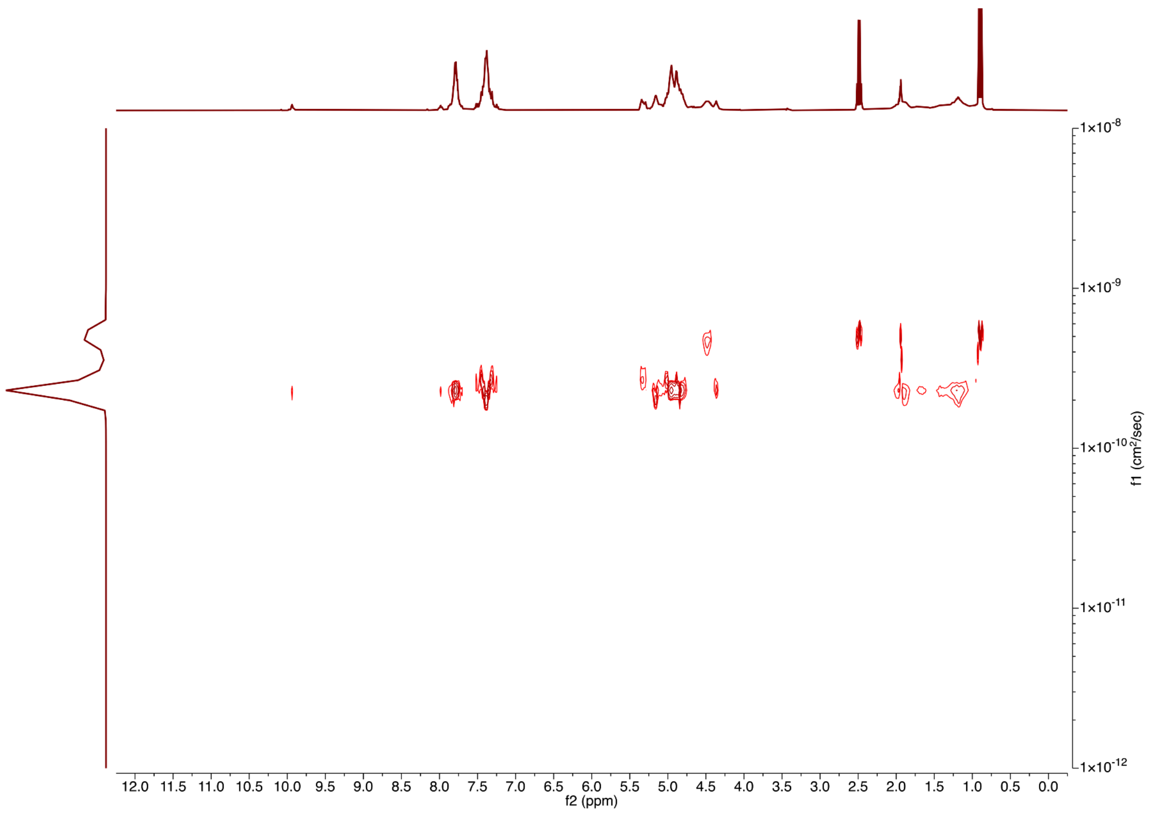

Figure S23: *Pseudo-2D DOSY plot of dynamer* ***3c*** *(1.5 M, CD_3CN).*

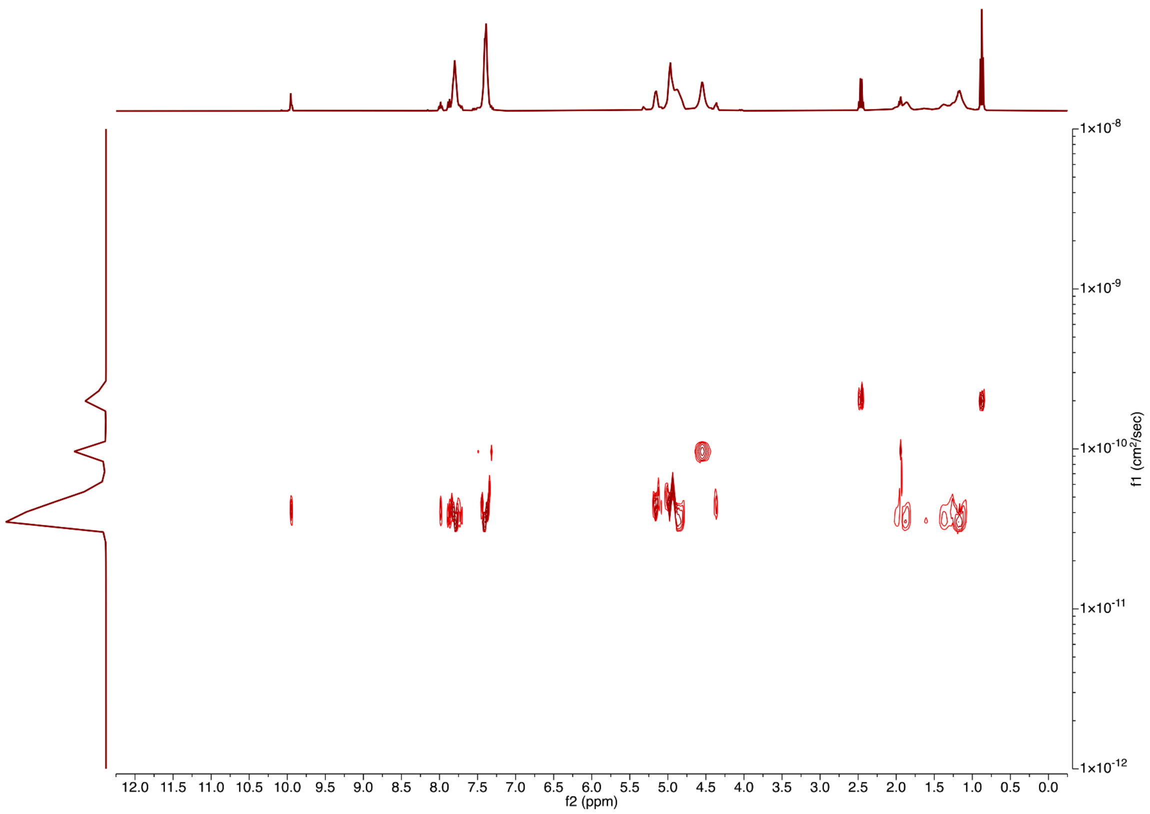

Figure S24: *Pseudo-2D DOSY plot of dynamer* ***3d*** *(1.5 M, CD_3CN).*

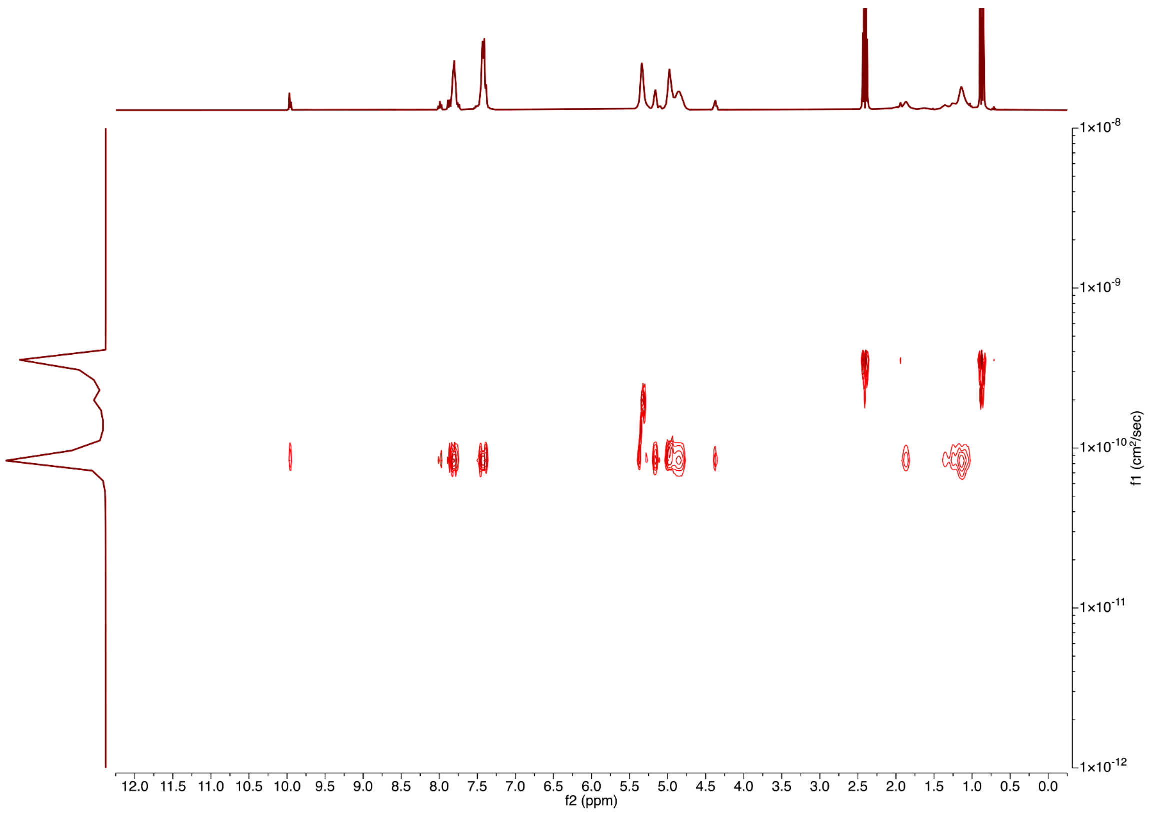

Figure S25: *Pseudo-2D DOSY plot of dynamer* ***3e*** *(1.5 M, CD_3CN).*

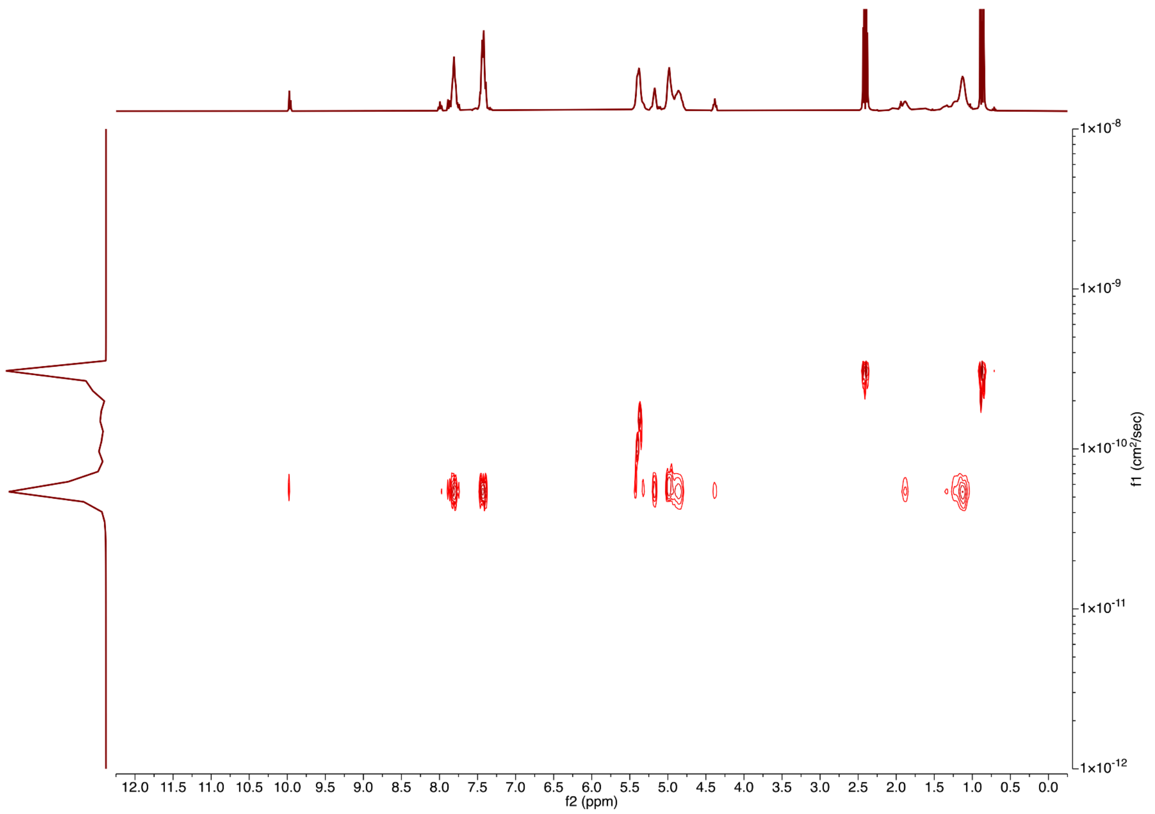

Figure S26: *Pseudo-2D DOSY plot of dynamer* ***3f*** *(1.5 M, CD_3CN).*

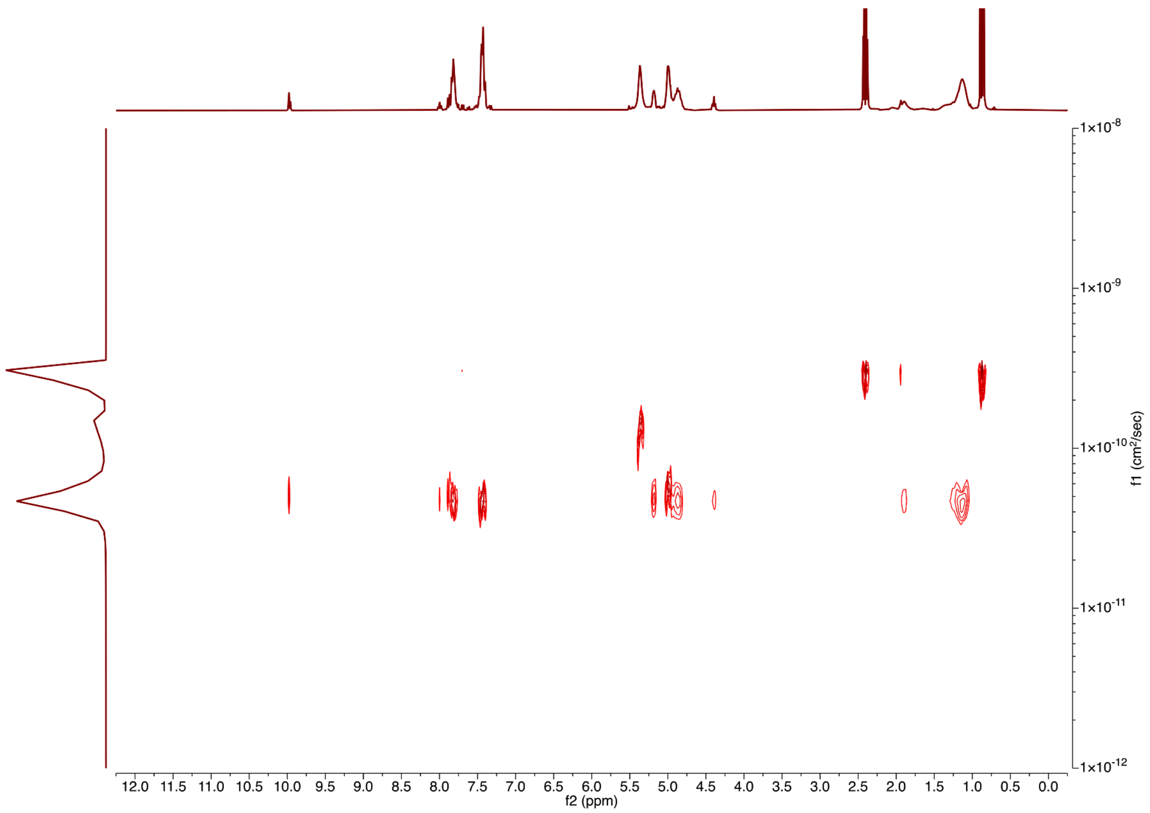

Figure S27: *Pseudo-2D DOSY plot of dynamer **3g** (1.5 M, CD_3CN).*

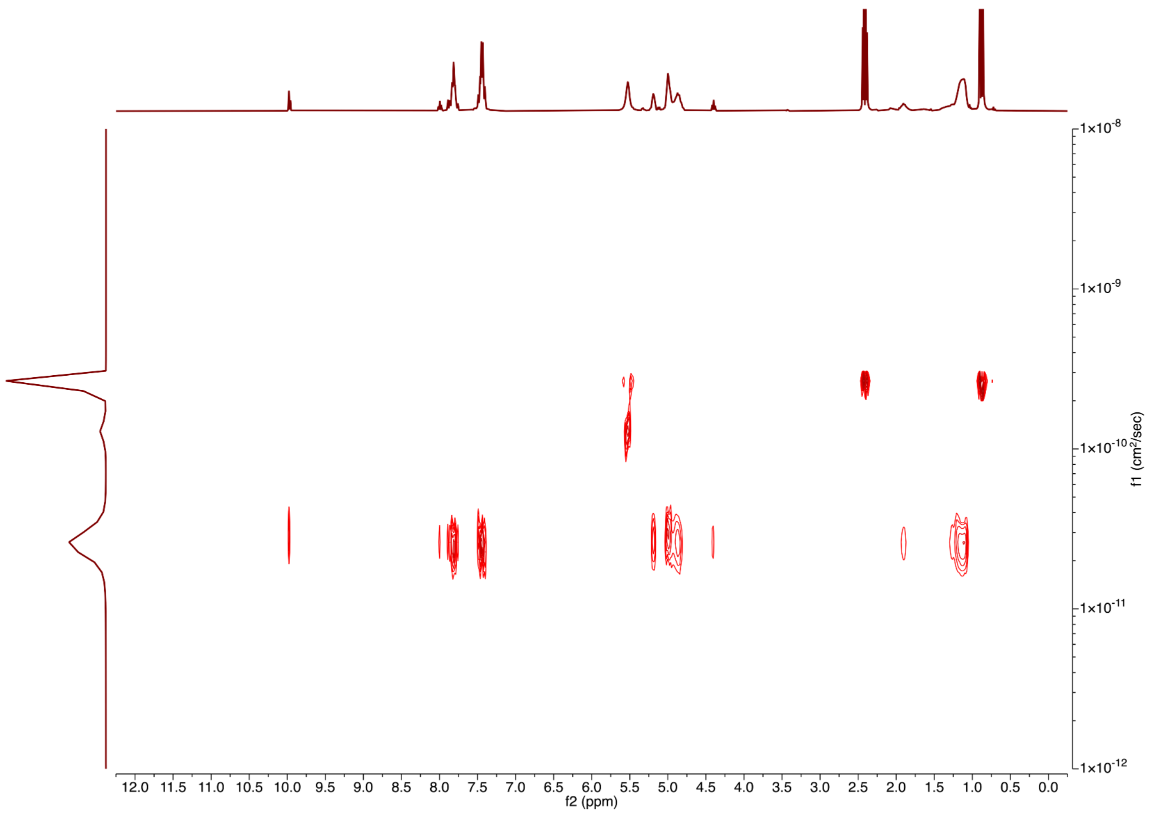

Figure S28: *Pseudo-2D DOSY plot of dynamer **3h** (1.5 M, CD_3CN).*

GPC chromatograms

All dynamer samples were prepared from compounds **1** and **2** in CD_3CN (1.5 M; 10 mol% Et_3N).

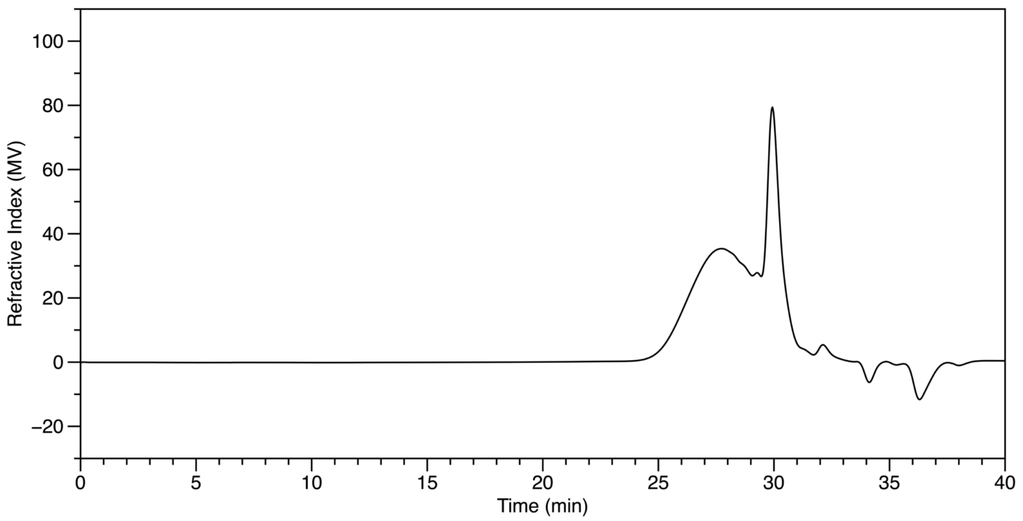

Figure S29: *GPC profile of dynamer **3a**.*

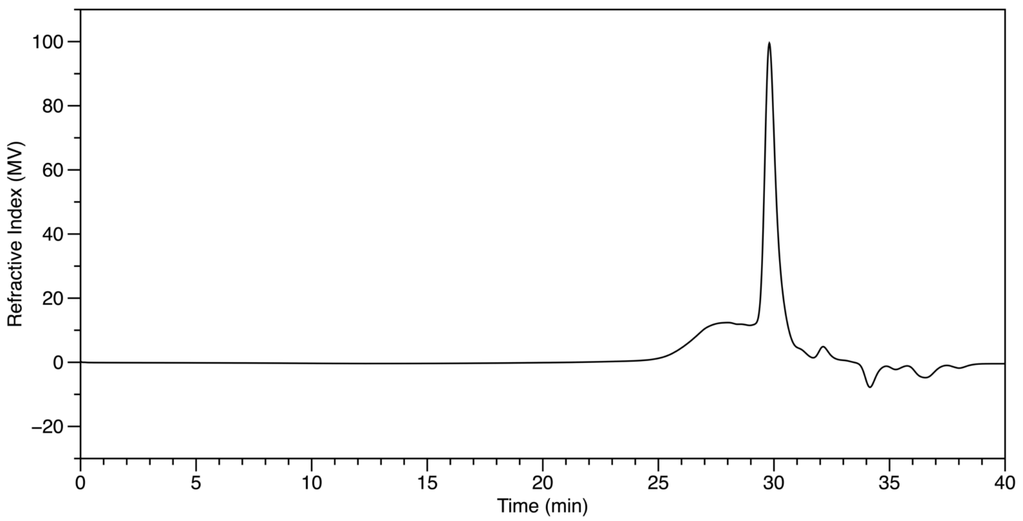

Figure S30: *GPC profile of dynamer **3b**.*

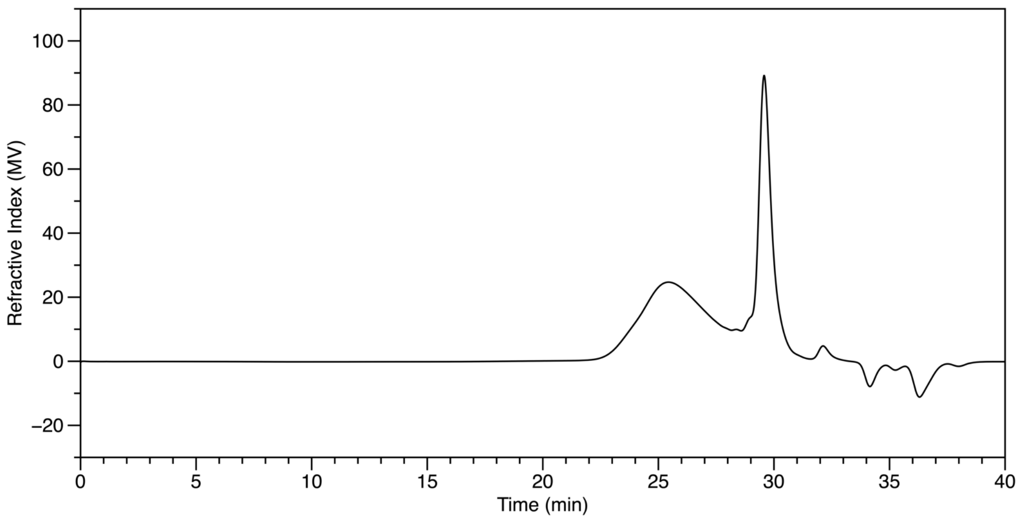

Figure S31: *GPC profile of dynamer **3c**.*

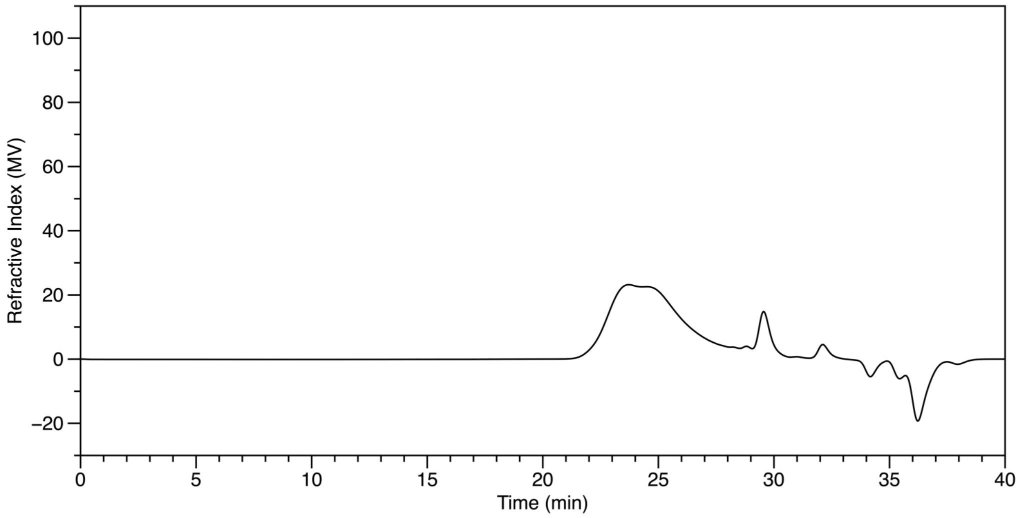

Figure S32: *GPC profile of dynamer **3d**.*

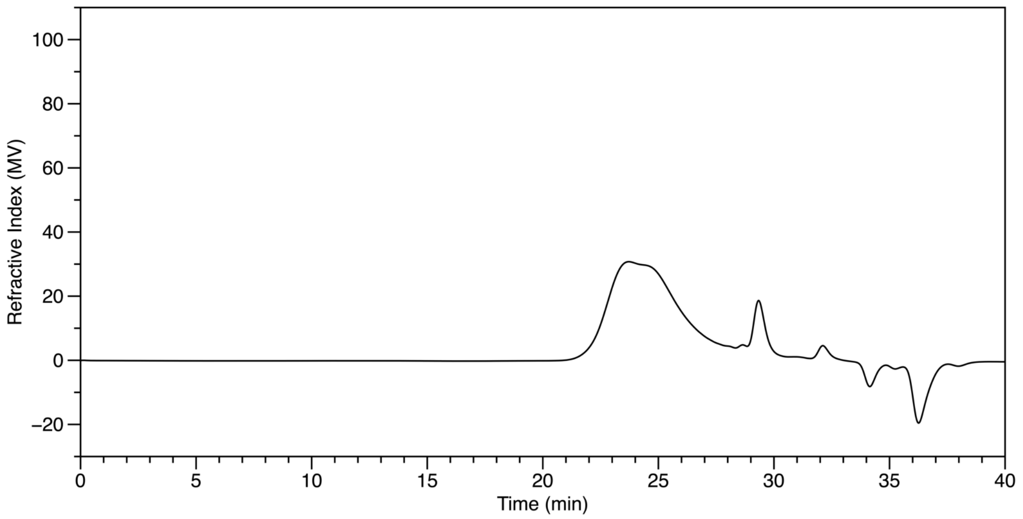

Figure S33: *GPC profile of dynamer **3e**.*

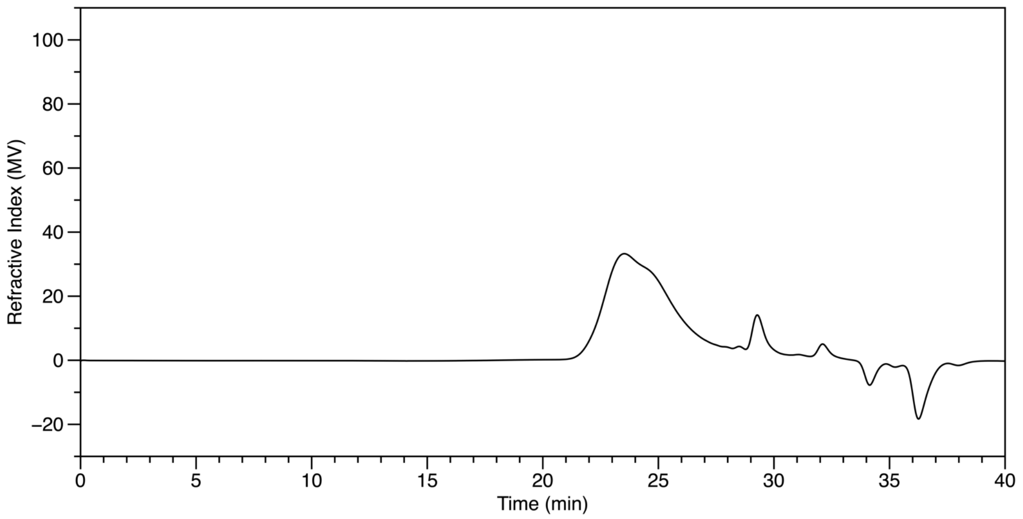

Figure S34: *GPC profile of dynamer **3f**.*

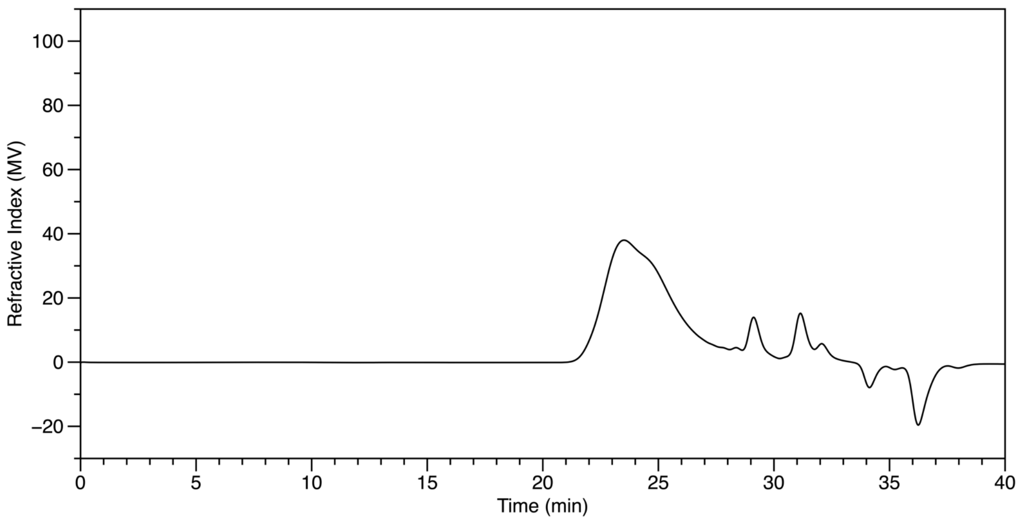

Figure S35: *GPC profile of dynamer **3g**.*

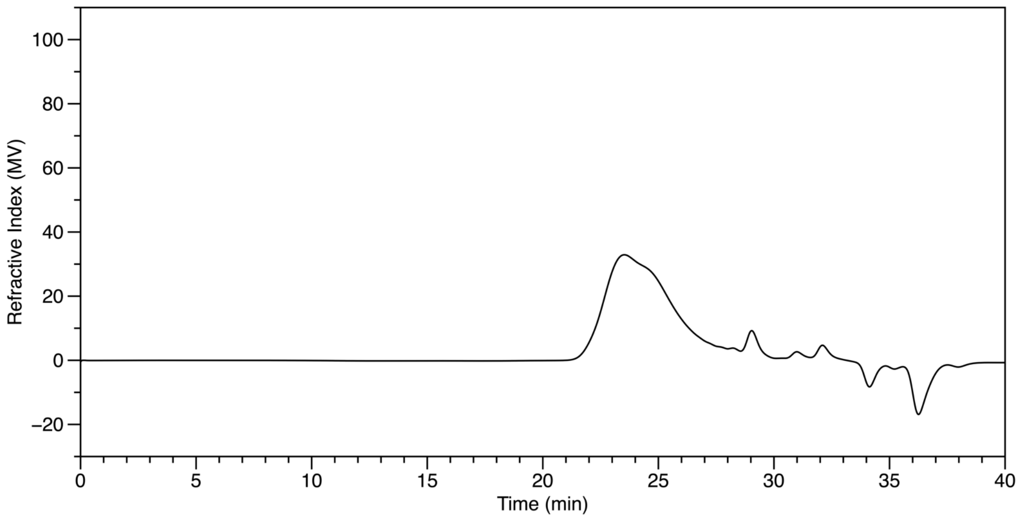

Figure S36: *GPC profile of dynamer **3h**.*

NMR, MS, and GPC data of lowellane macrocycles

NMR spectra

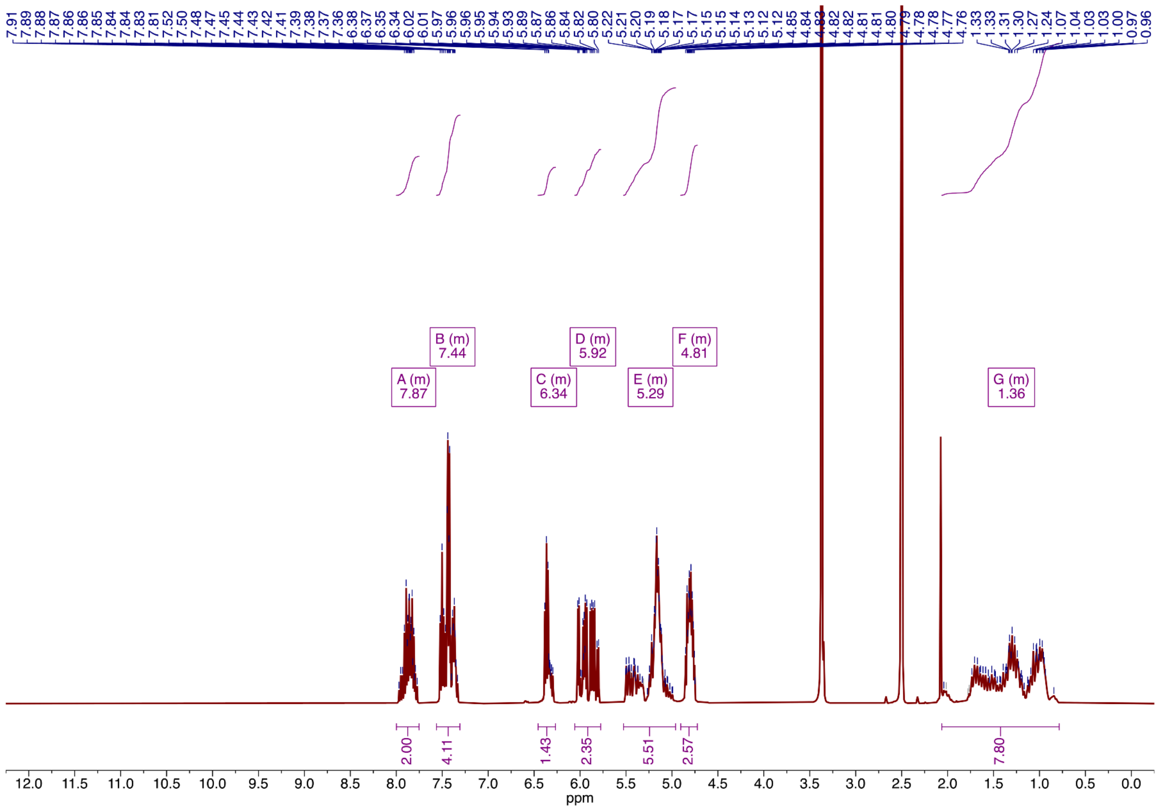

Figure S37: ^{1}*H NMR spectrum of lowellane **7** in DMSO-d$_6$.*

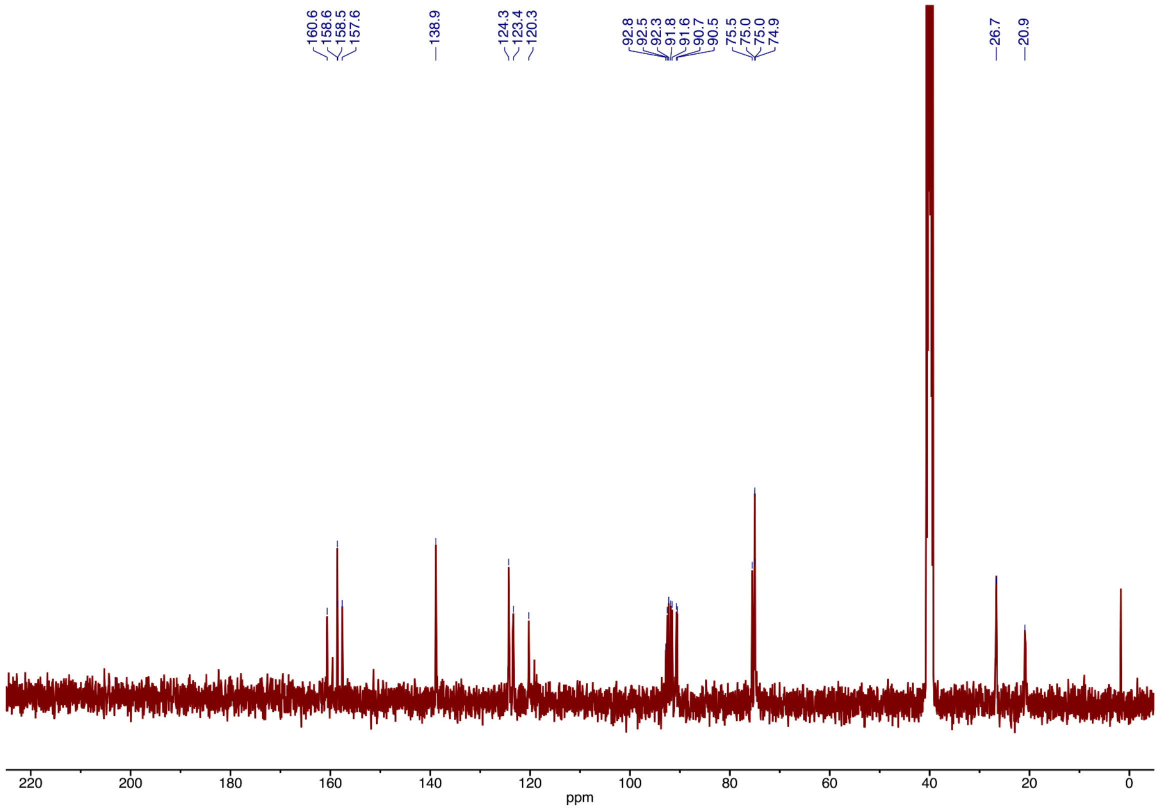

Figure S38: ^{13}*C NMR spectrum of lowellane **7** in DMSO-d$_6$.*

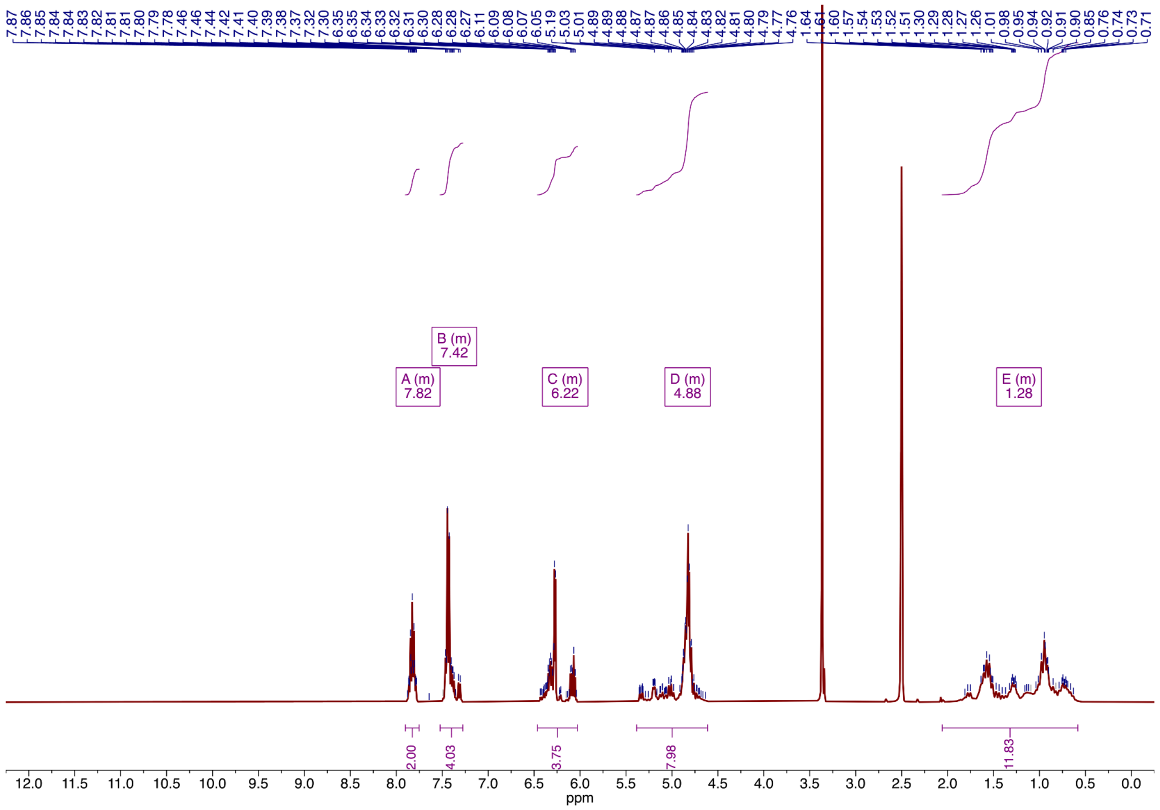

Figure S39: ^1H NMR spectrum of lowellane **8** in DMSO-d_6.

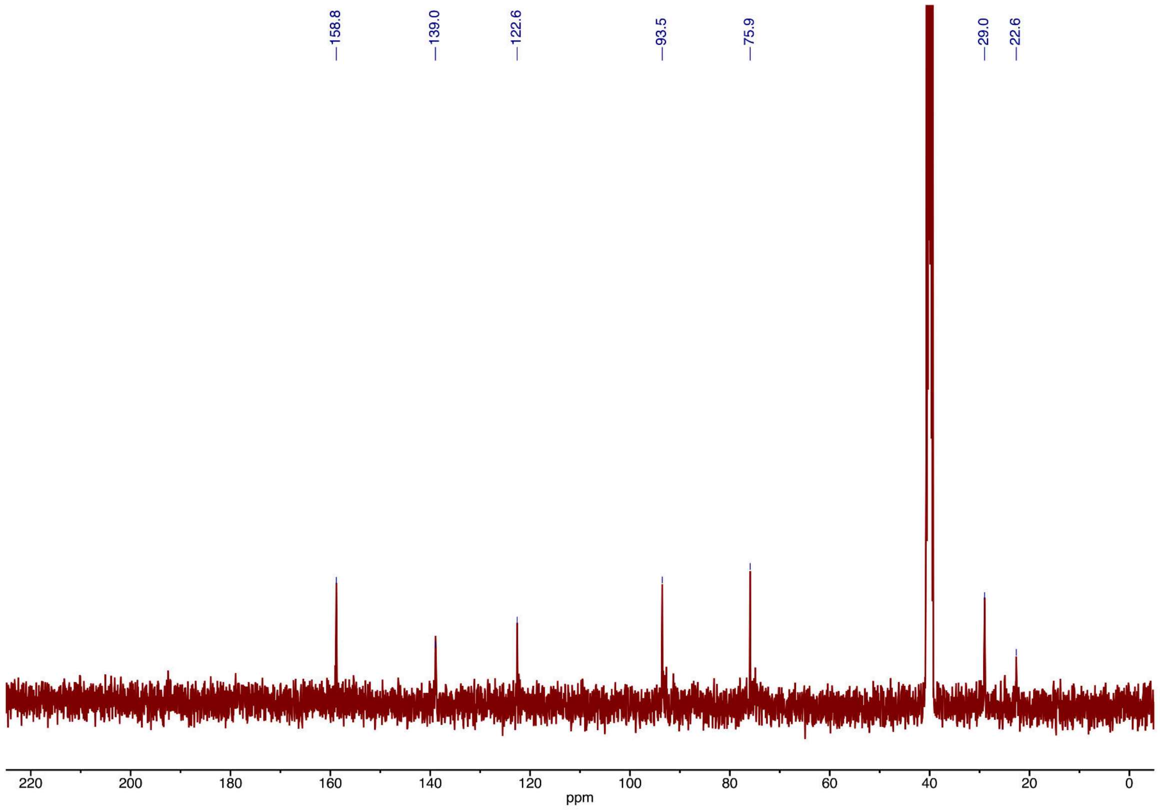

Figure S40: ^{13}C NMR spectrum of lowellane **8** in DMSO-d_6.

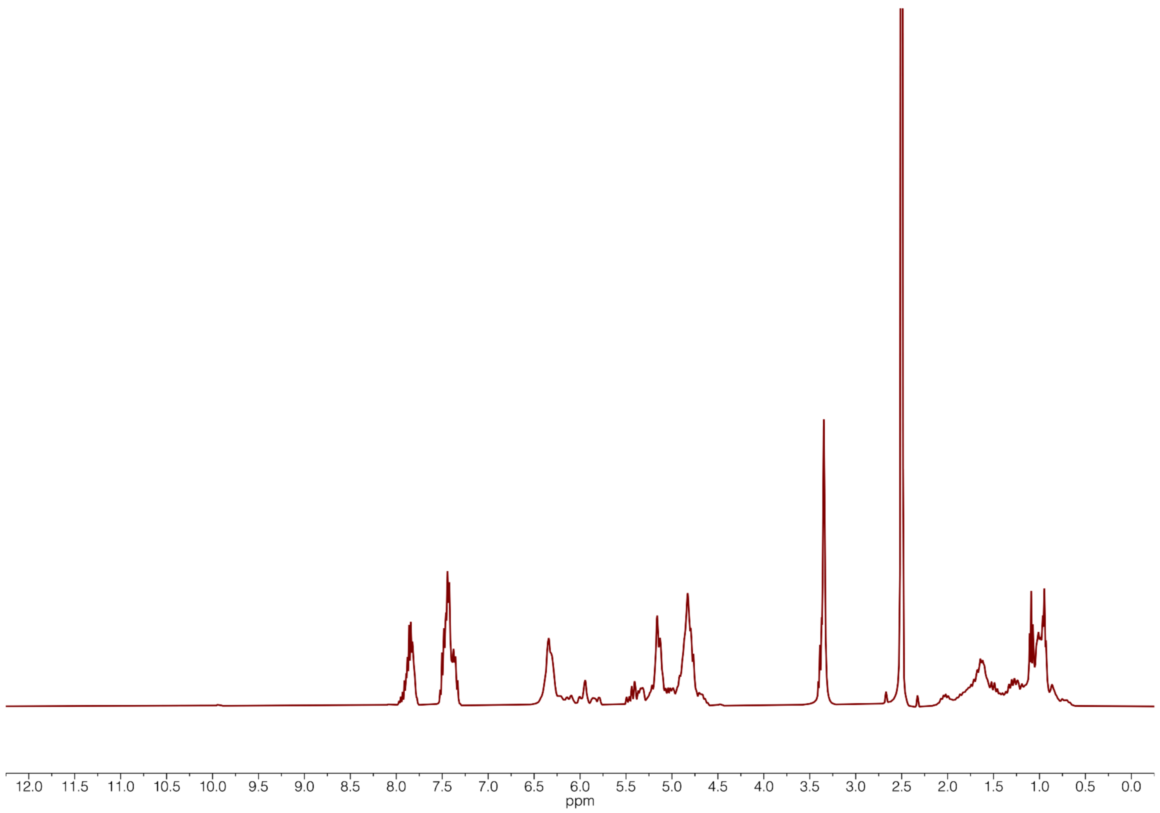

Figure S41: *^{1}H NMR spectrum of the mixture of lowellanes **7**, **8** and **9** in DMSO-d$_6$.*

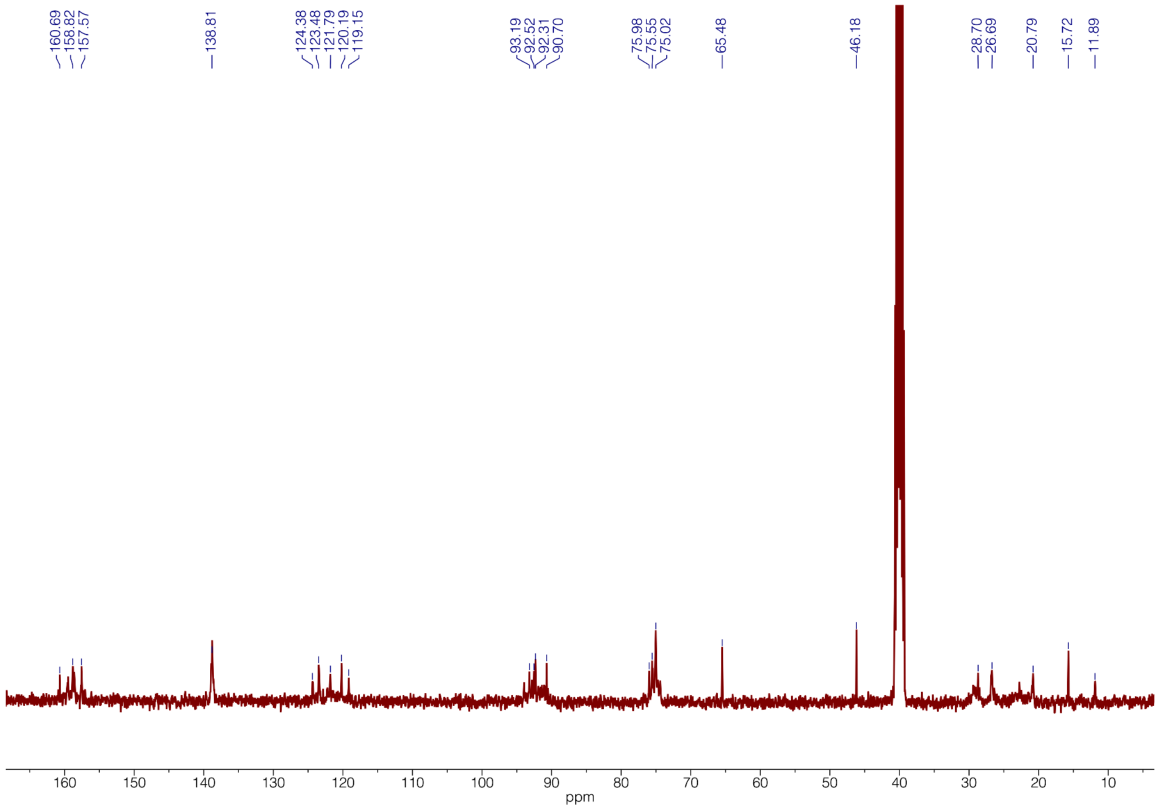

Figure S42: *^{13}C NMR spectrum of the mixture of lowellanes **7**, **8** and **9** in DMSO-d$_6$.*

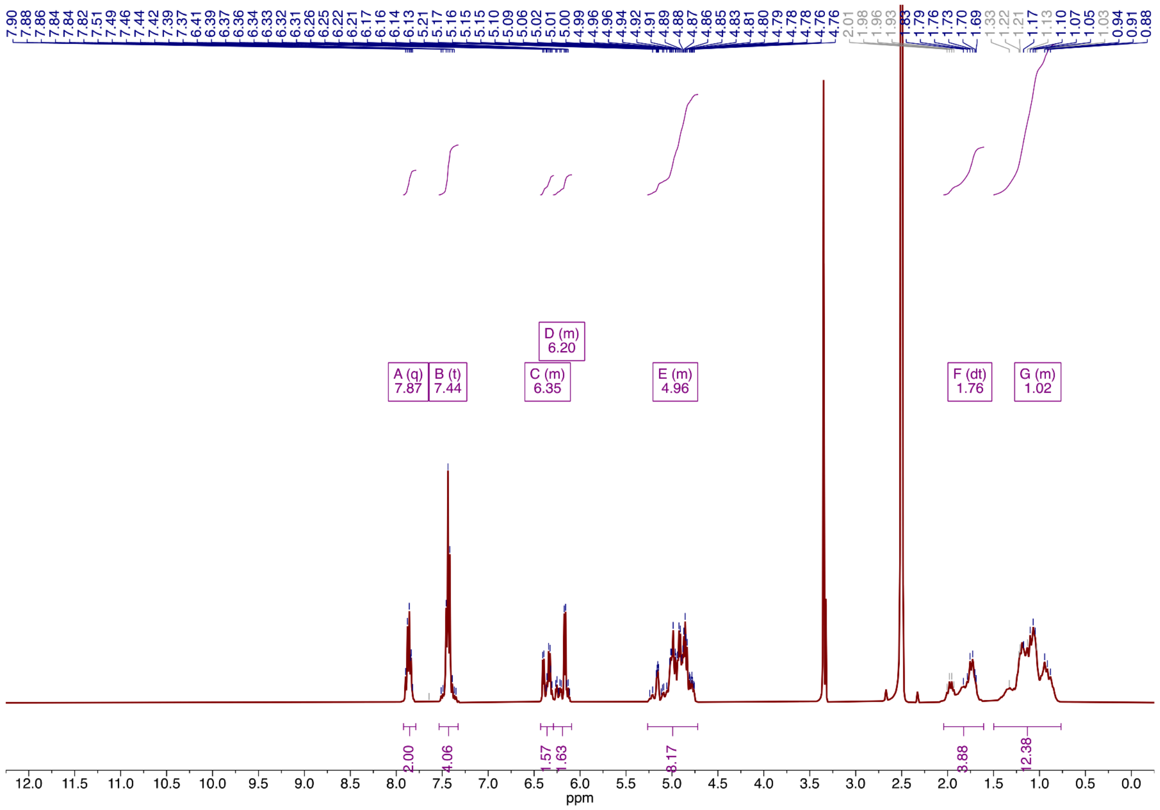

Figure S43: *^{1}H NMR spectrum of lowellane* ***10*** *in DMSO-d_6.*

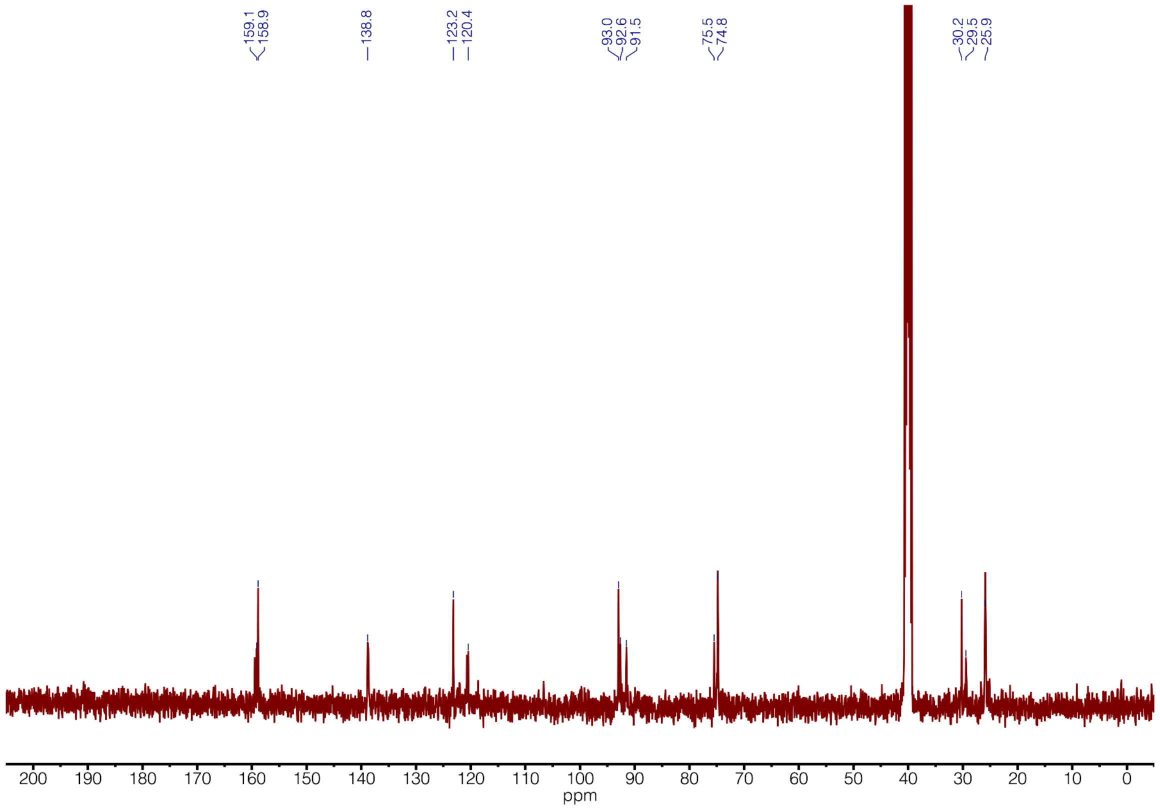

Figure S44: *^{13}C NMR spectrum of lowellane* ***10*** *in DMSO-d_6.*

MS spectra

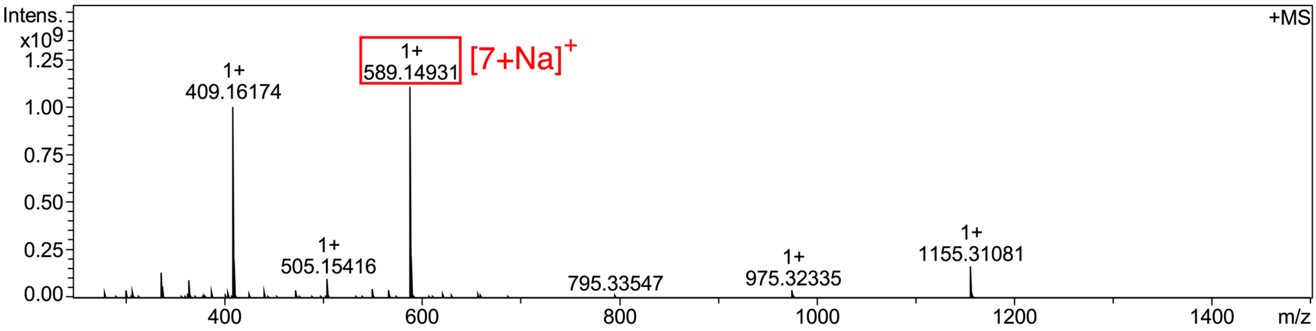

Figure S45: *HRMS spectrum of lowellane **7**.*

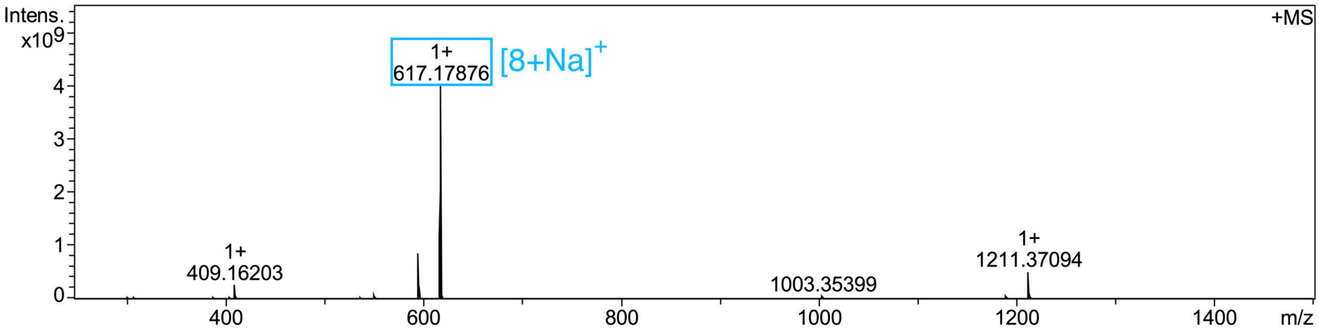

Figure S46: *HRMS spectrum of lowellane **8**.*

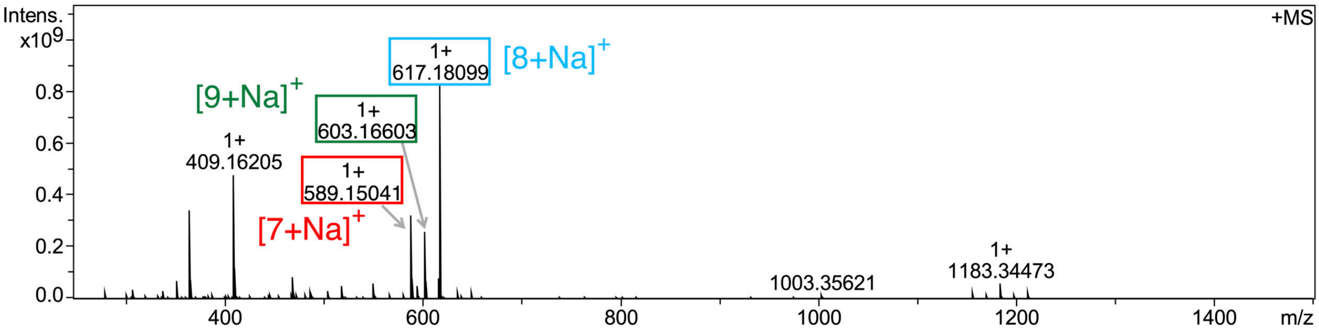

Figure S47: *HRMS spectrum of mixture of lowellanes **7**, **8**, and **9**.*

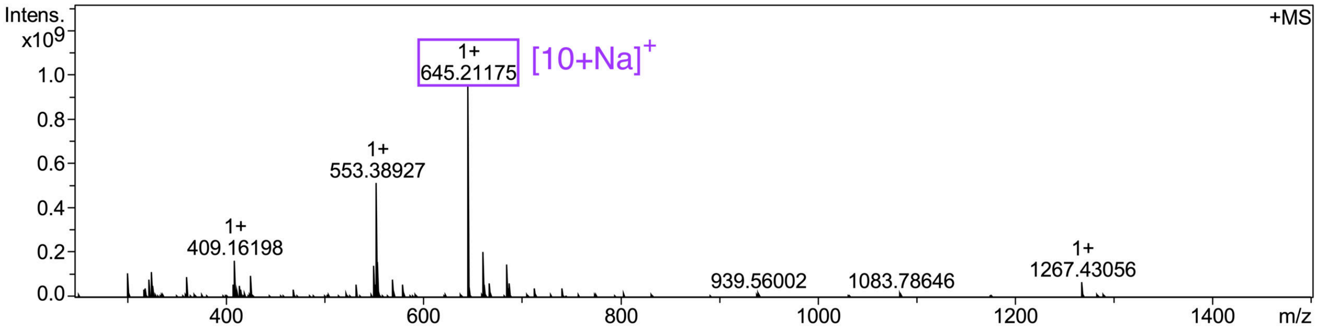

Figure S48: *HRMS spectrum of lowellane **10**.*

GPC profile of reaction between compounds 1 and 2b

2,6-Pyridinedicarboxaldehyde (**1**, 0.135 g, 1 mmol) and 1,4-dinitrobutane (**2b**, 0.148g, 1 mmol) were dissolved in CH_3CN (1 mL), after which NEt_3 (14 µL, 0.1 mmol) was added. Aliquots (100 µL) of the solution were extracted periodically, the solvent was replaced by THF, and the samples were analyzed by GPC.

Table S1: *GPC analysis of dynamer **3b** formation.*

Reaction time (h)	M_n (g/mol)	M_w (g/mol)	M_z (g/mol)	*Đ*
1	3700	4500	5500	1.2
2	3500	4200	4900	1.2
3	3700	4400	5300	1.2
4	3500	4100	4900	1.2
5	3100	3600	4100	1.1
6	3100	3600	4100	1.1
9	2800	3000	3300	1.9

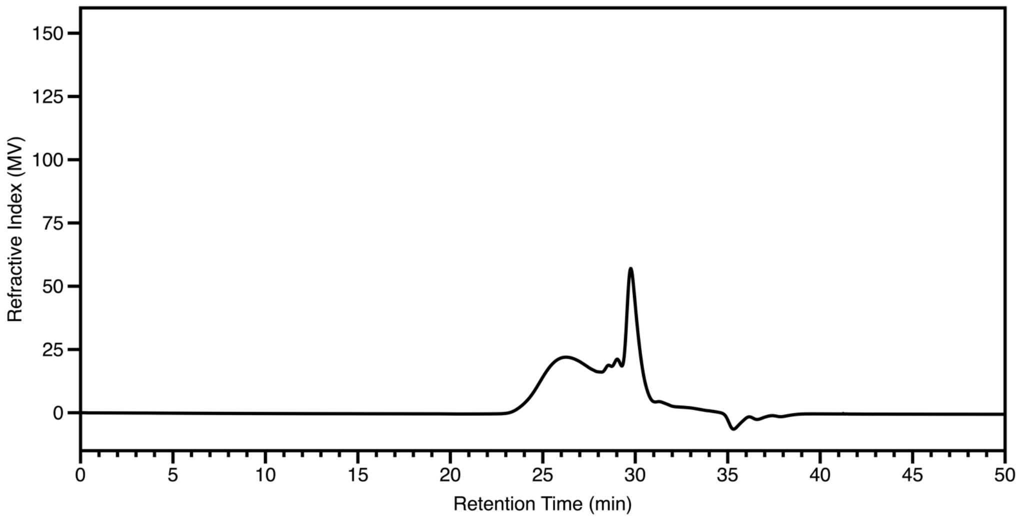

Figure S49: *GPC profile of reaction between **1** and **2b** after 1 h (0.5 M, 10 mol% Et_3N).*

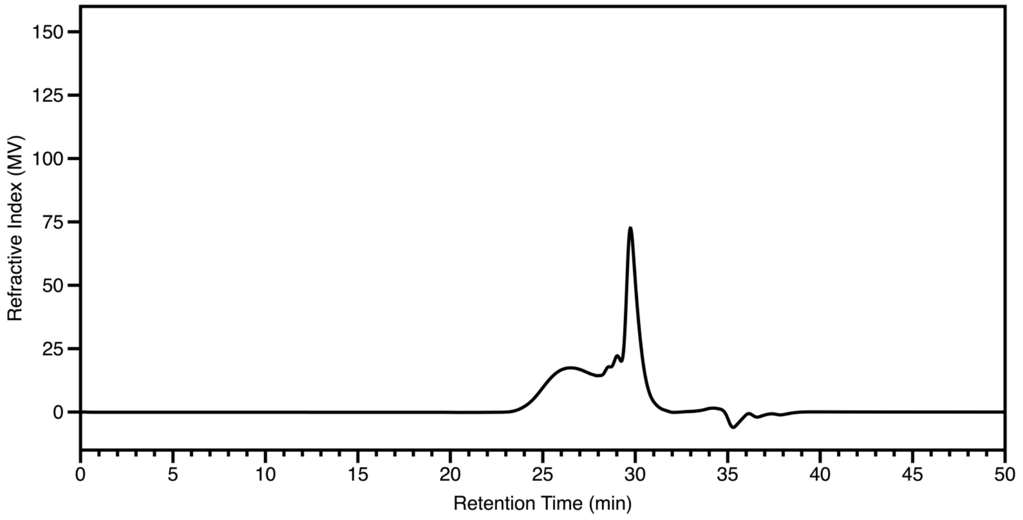

Figure S50: *GPC profile of reaction between **1** and **2b** after 2 h (0.5 M, 10 mol% Et_3N).*

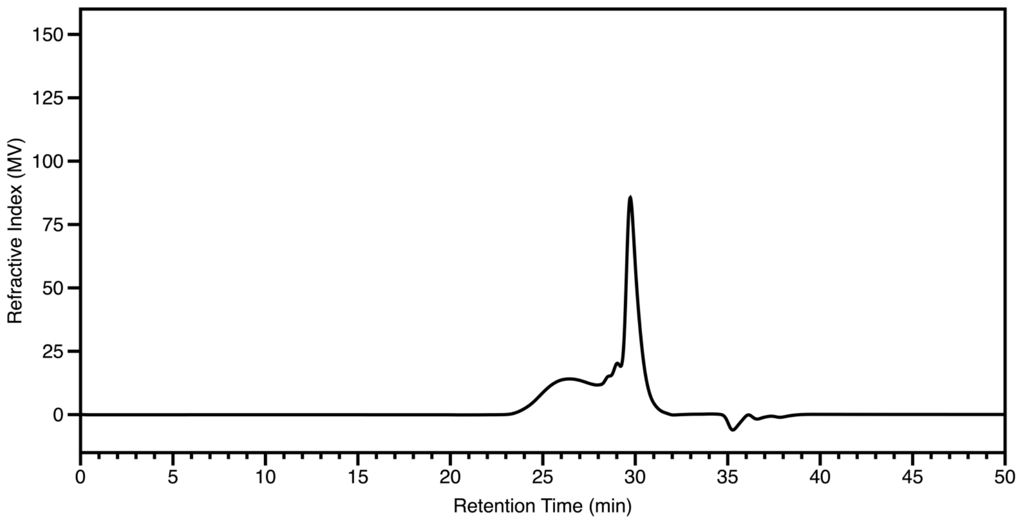

Figure S51: *GPC profile of reaction between **1** and **2b** after 3 h (0.5 M, 10 mol% Et_3N).*

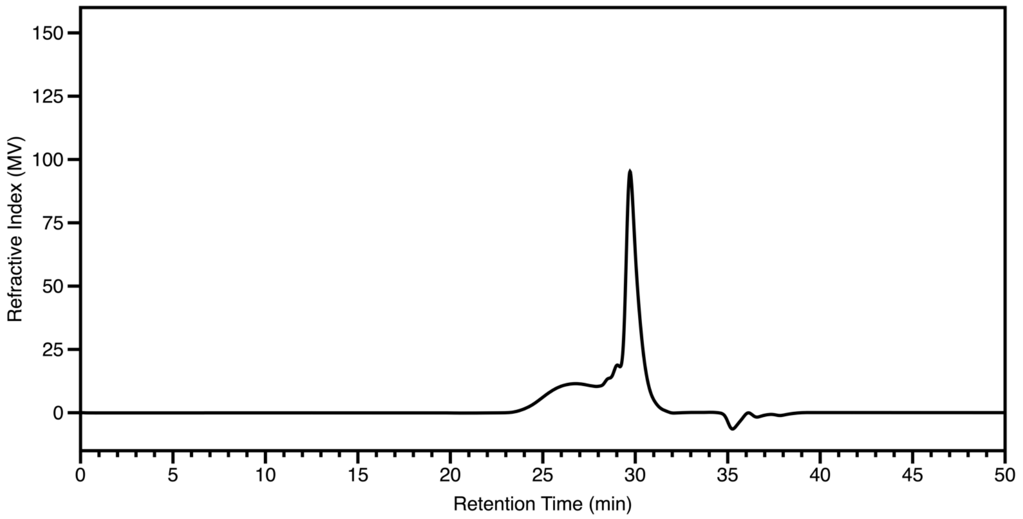

Figure S52: *GPC profile of reaction between **1** and **2b** after 4 h (0.5 M, 10 mol% Et_3N).*

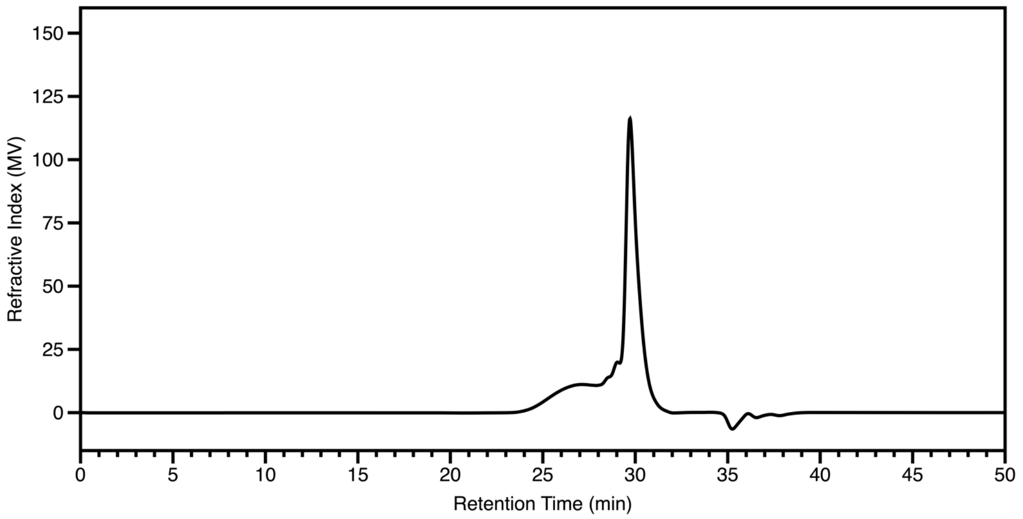

Figure S53: *GPC profile of reaction between **1** and **2b** after 5 h (0.5 M, 10 mol% Et_3N).*

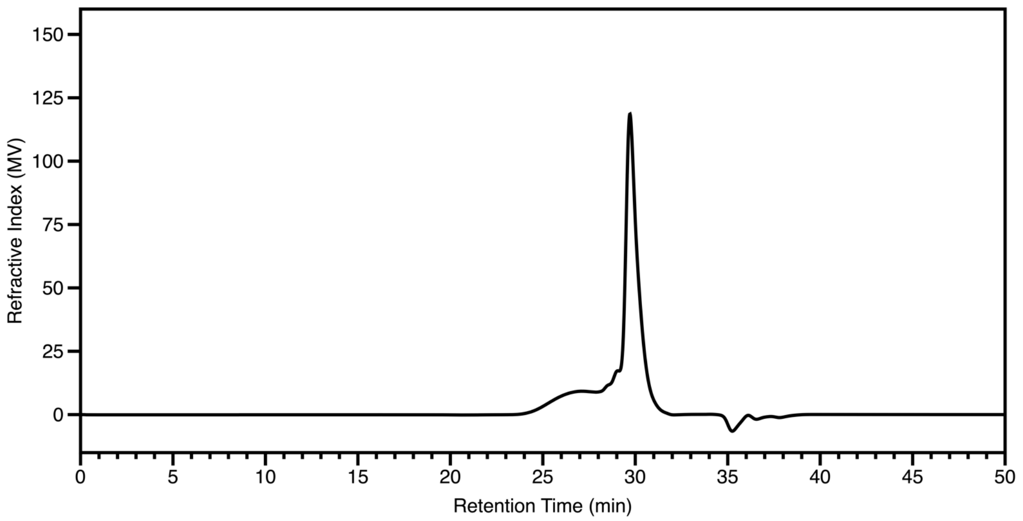

Figure S54: *GPC profile of reaction between **1** and **2b** after 6 h (0.5 M, 10 mol% Et_3N).*

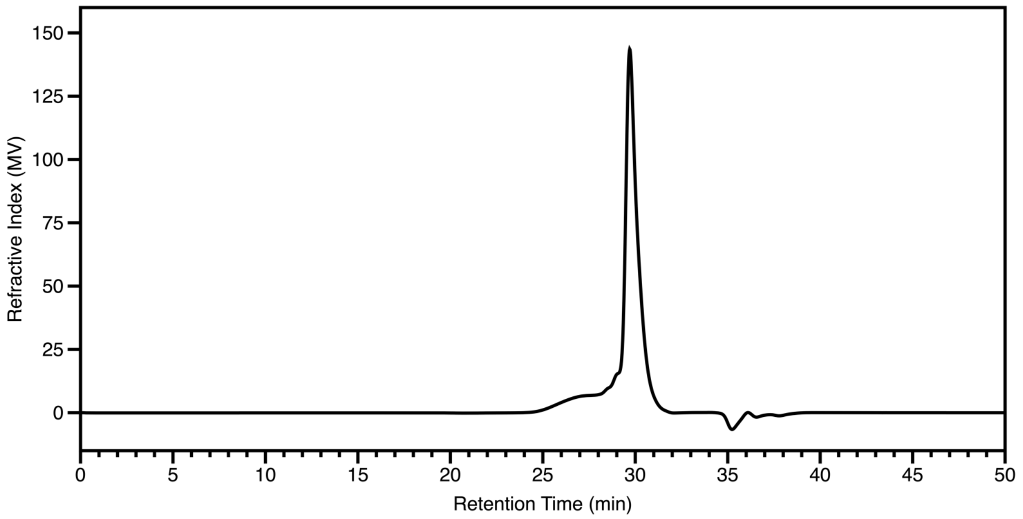

Figure S55: *GPC profile of reaction between **1** and **2b** after 9 h (0.5 M, 10 mol% Et_3N).*

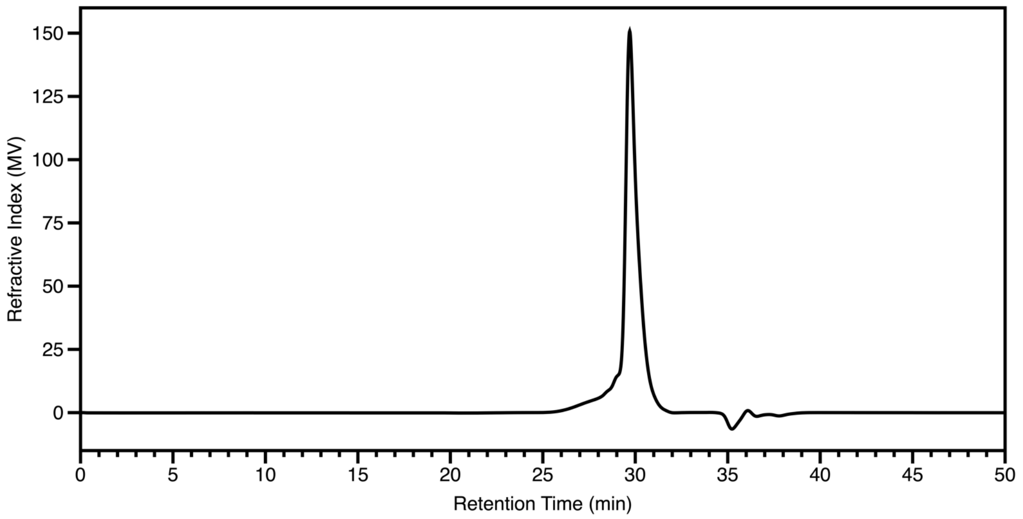

Figure S56: *GPC profile of reaction between **1** and **2b** after 12 h (0.5 M, 10 mol% Et_3N).*

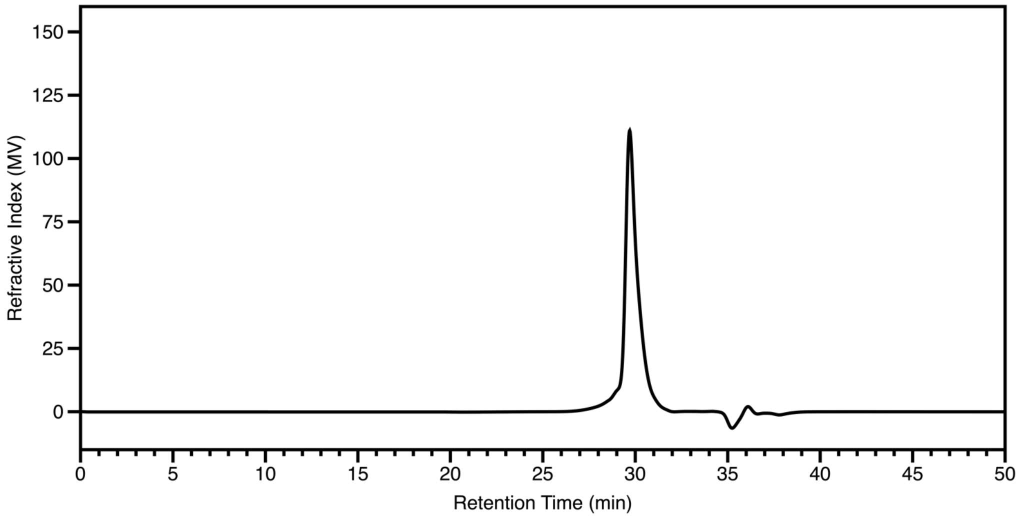

Figure S57: *GPC profile of reaction between* ***1*** *and* ***2b*** *after 24 h (0.5 M, 10 mol% Et_3N).*

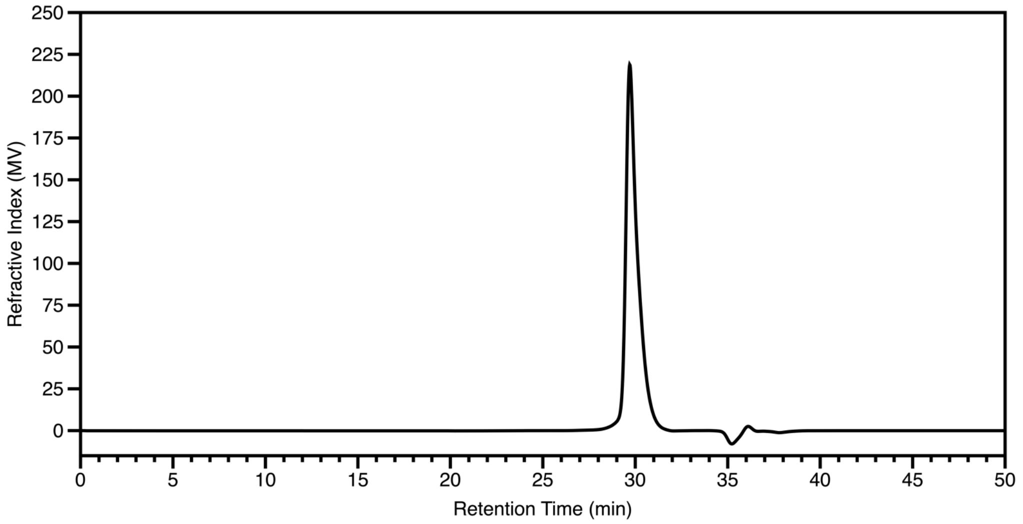

Figure S58: *GPC profile of lowellane* ***7****.*

Reaction kinetics

A study of the reaction kinetics was carried out by adding Et_3N (10 mol%) to a CD_3CN solution of **1** and **2d** (0.5 M) in an NMR tube. The reaction was monitored by NMR with a 3 min time interval within the first hour, followed by an hourly interval from 1 h to 12 h.

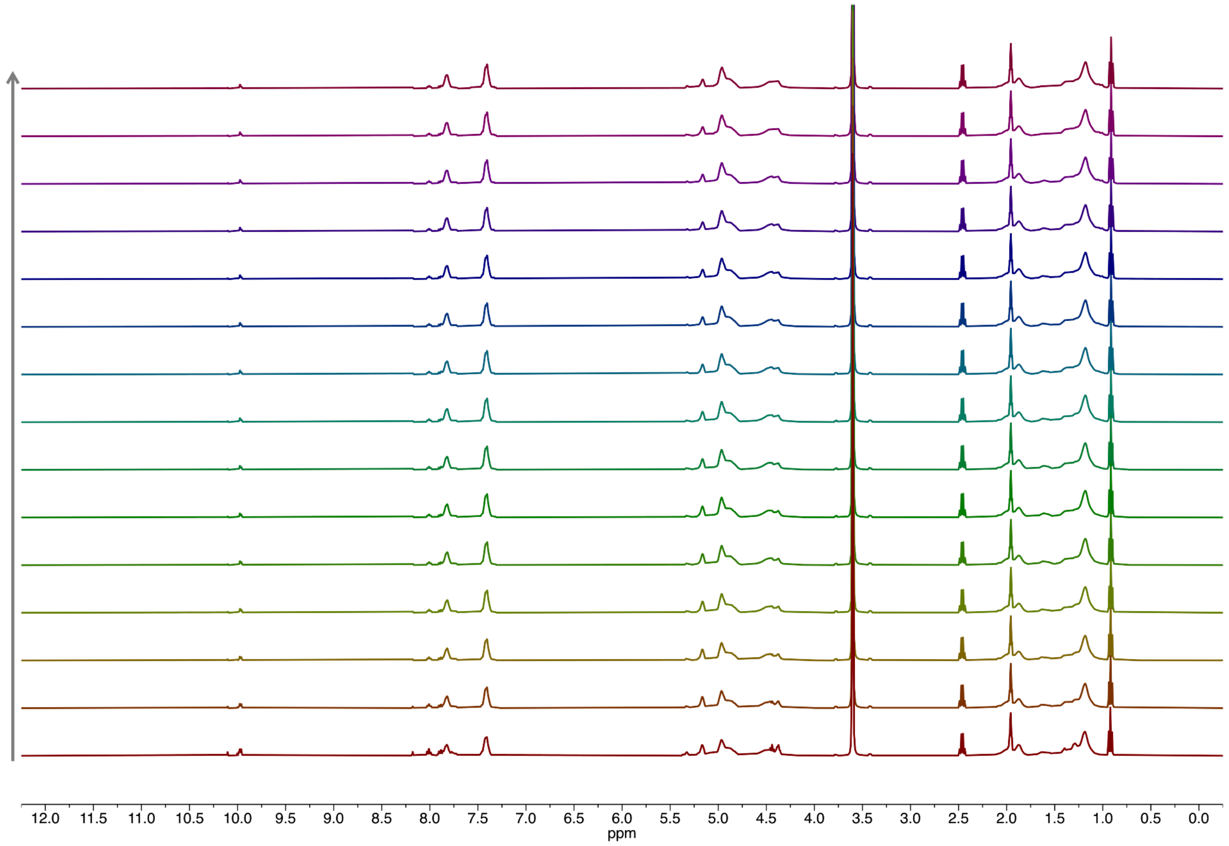

Figure S59: *^{1}H NMR spectra of reaction between compounds **1** and **2d** from 1 h to 12 h. (1 h intervals; 0.5 M, 10 mol% Et_3N; 1,4-dioaxane as internal standard; CD_3CN).*

2,6-Pyridinedicarboxaldehyde (**1**, 0.135 g, 1 mmol) and 1,6-dinitrohexane (**2d**, 0.176 g, 1 mmol) were dissolved in CH_3CN (2 mL), to which solution triethylamine (14 µL, 0.1 mmol) was added. Aliquots (100 µL) of the solution were extracted every 6 min during the first hour, the solvent replaced by THF, and the resulting solutions analyzed by GPC.

Table S2: *GPC data of dynamer **3d** formation.*

Reaction Time (min)	M_n (g/mol)	M_w (g/mol)	M_z (g/mol)	*Đ*
6	800	900	900	1.0
12	1100	1200	1200	1.0
18	2000	2300	2600	1.1
24	1800	2000	2200	1.1
30	2200	2700	3300	1.2
36	2400	3100	3900	1.3
42	3100	4300	6200	1.4
48	3500	5100	7700	1.5
54	4000	6400	10300	1.6
60	4100	6400	10000	1.6

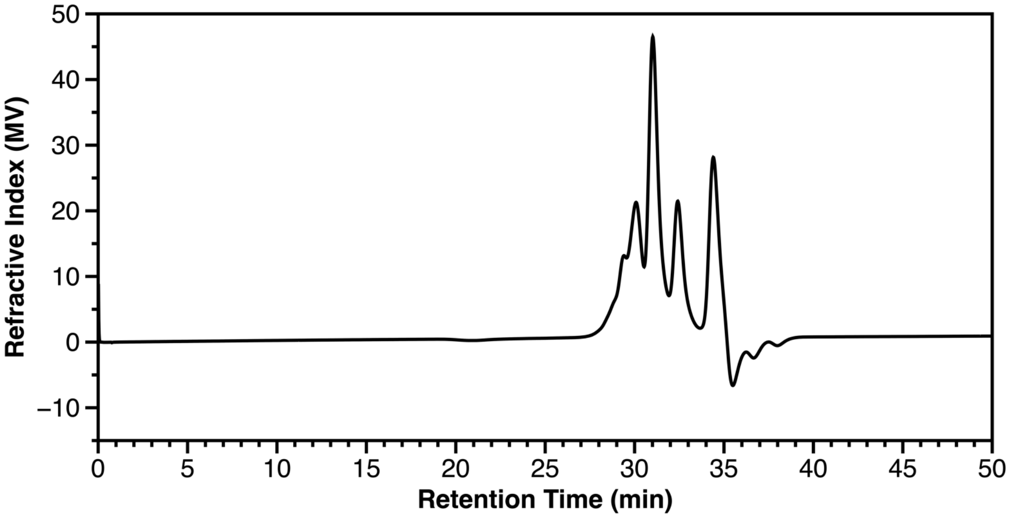

Figure S60: *GPC profile of reaction between compounds* ***1*** *and* ***2d*** *after 6 min.*

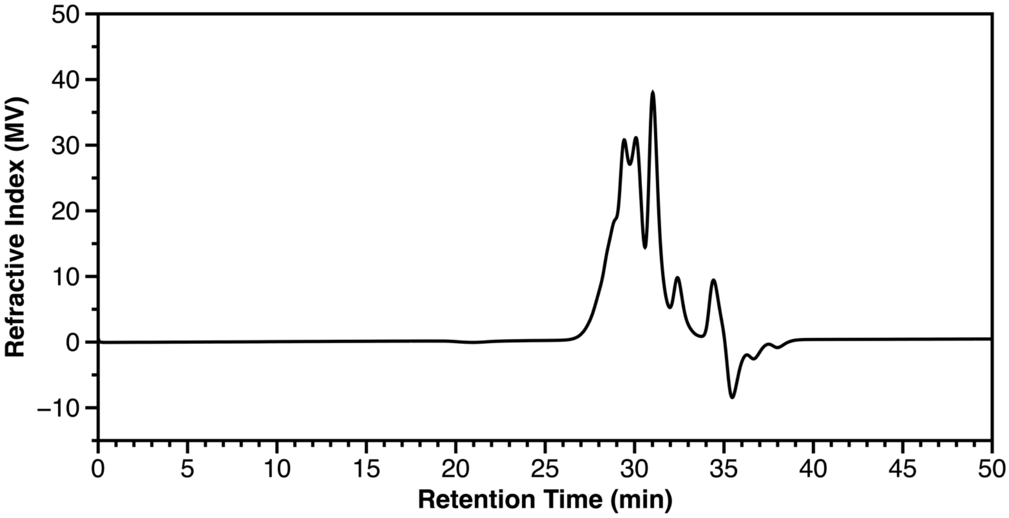

Figure S61: *GPC profile of reaction between compounds* ***1*** *and* ***2d*** *after 12 min.*

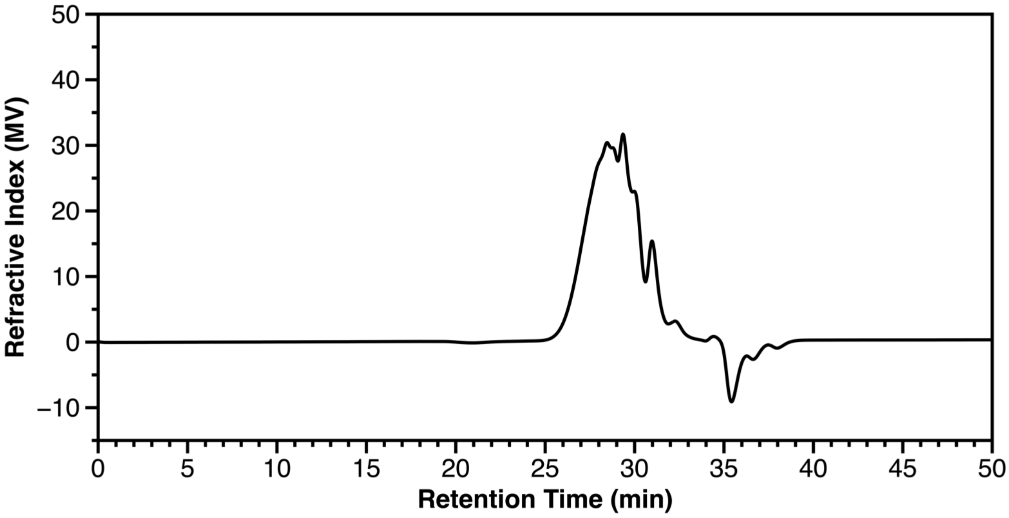

Figure S62: *GPC profile of reaction between compounds* ***1*** *and* ***2d*** *after 18 min.*

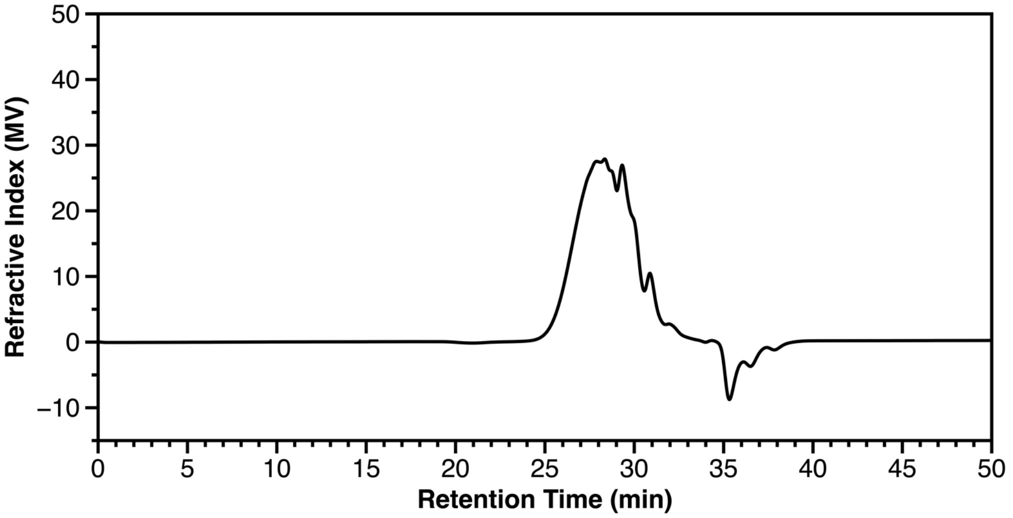

Figure S63: *GPC profile of reaction between compounds **1** and **2d** after 24 min.*

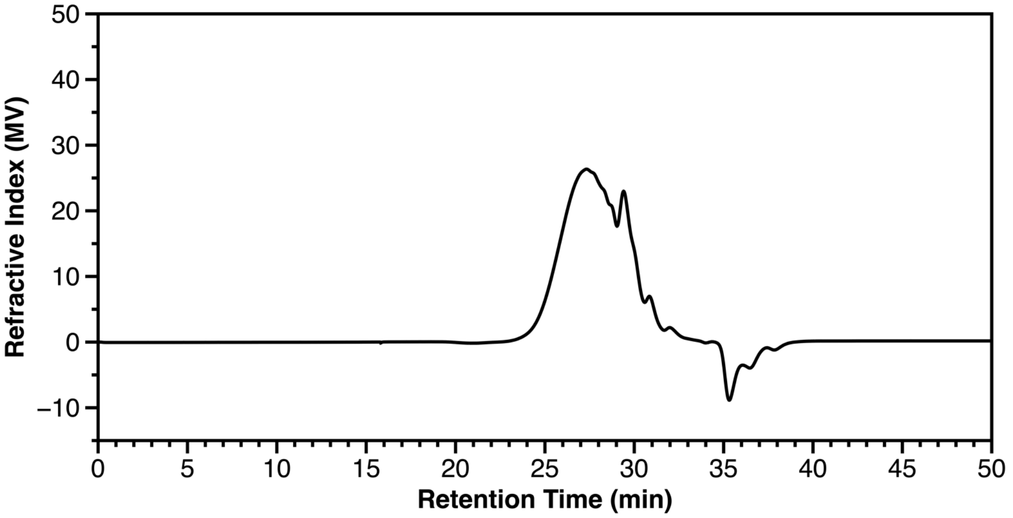

Figure S64: *GPC profile of reaction between compounds **1** and **2d** after 30 min.*

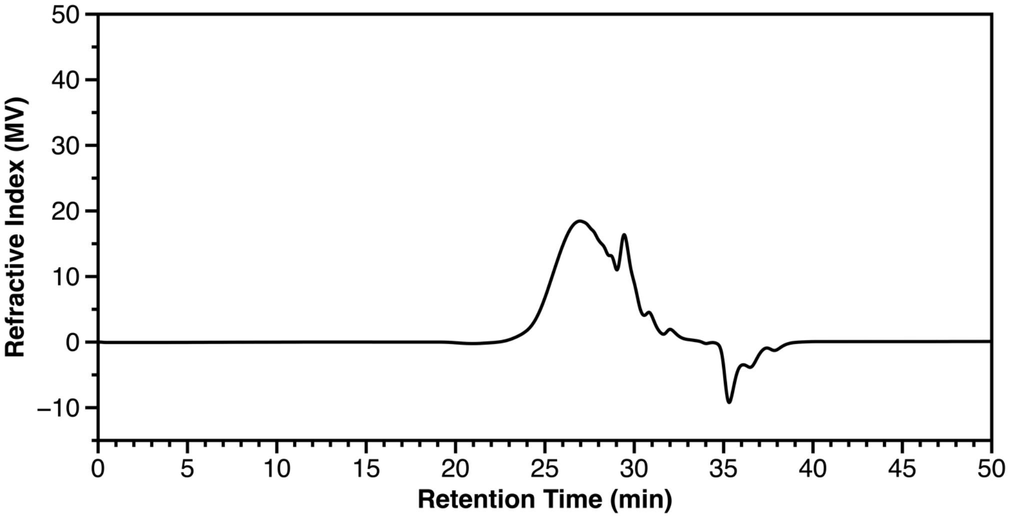

Figure S65: *GPC profile of reaction between compounds **1** and **2d** after 36 min.*

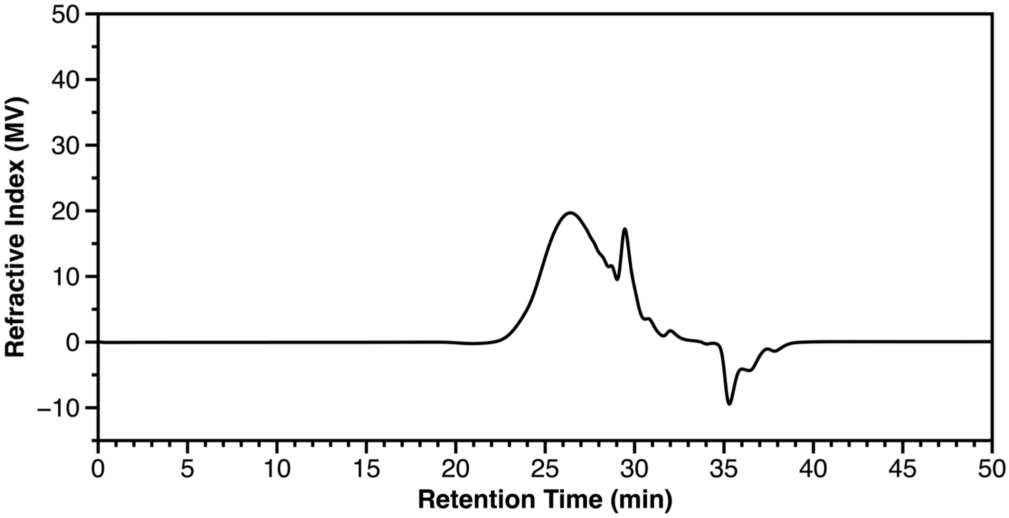

__Figure S66:__ GPC profile of reaction between compounds __1__ and __2d__ after 42 min.

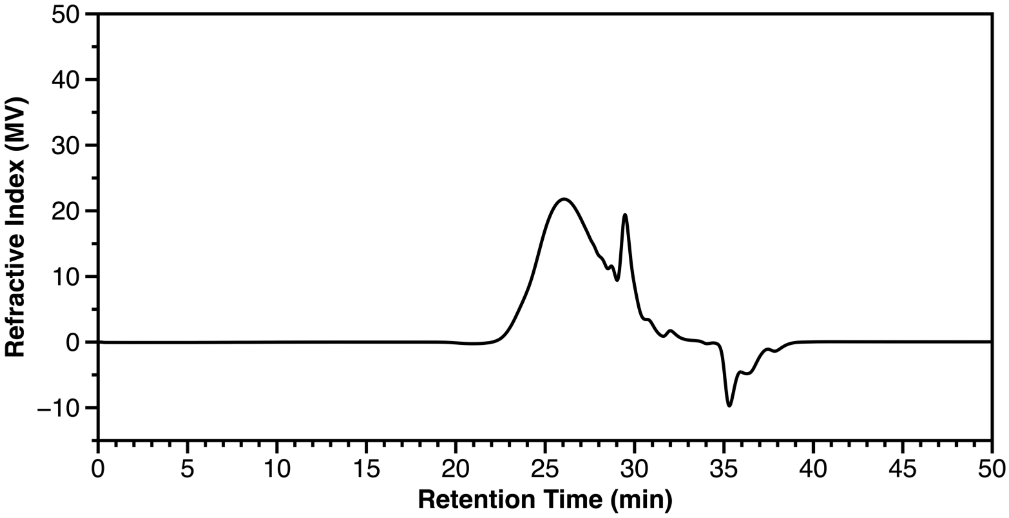

__Figure S67:__ GPC profile of reaction between compounds __1__ and __2d__ after 48 min.

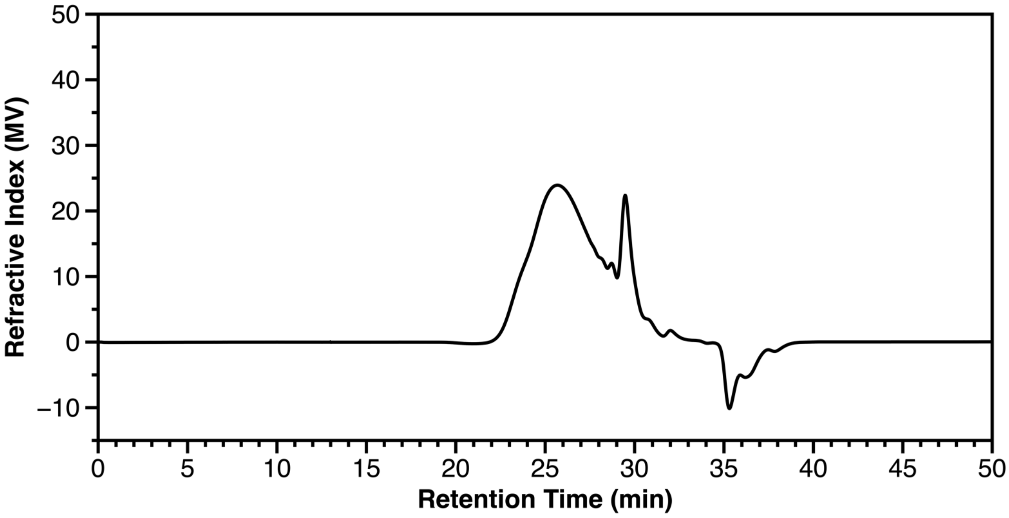

__Figure S68:__ GPC profile of reaction between compounds __1__ and __2d__ after 54 min.

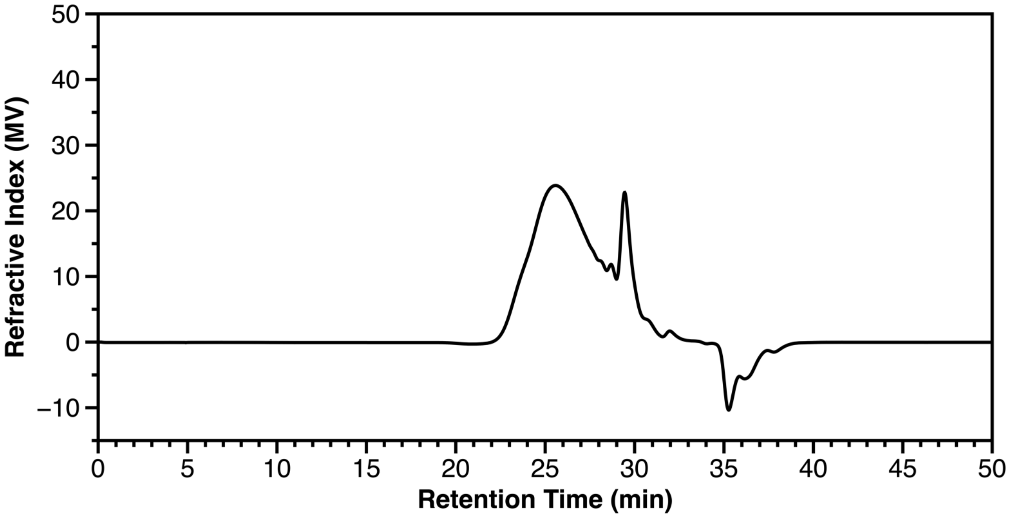

Figure S69: *GPC profile of reaction between compounds* ***1*** *and* ***2d*** *after 60 min.*

Effects of base

Reaction kinetics

Different amounts of Et_3N were added to solutions of compounds **1** and **3d** (0.5 M, CD_3CN) in NMR tubes. The reactions were monitored by NMR with a 3 min time interval for 60 min. All samples were tested by NMR after 12 h.

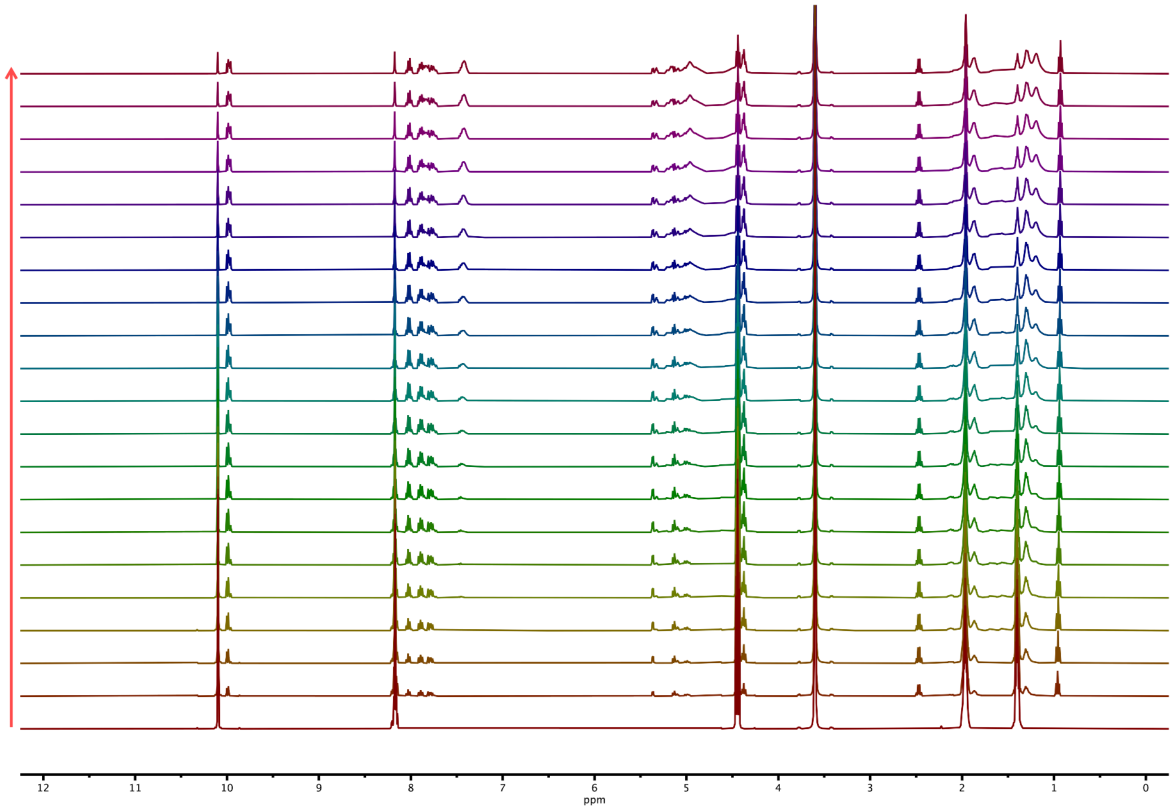

Figure S70: *¹NMR spectra of dynamer* ***3d*** *with 5 mol% Et_3N (0 min-60 min; 0.5 M; CD_3CN).*

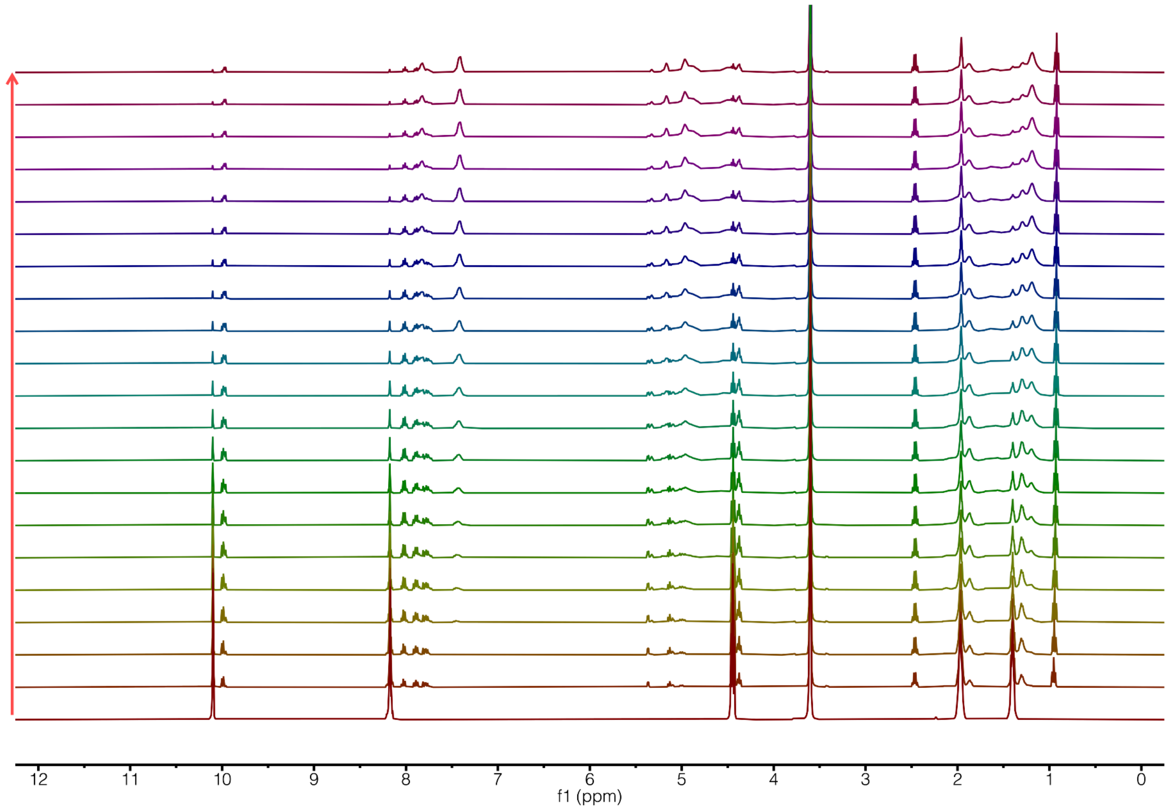

Figure S71: *[1]NMR spectra of dynamer* ***3d*** *with 10 mol% Et_3N (0 min-60 min; 0.5 M; CD_3CN).*

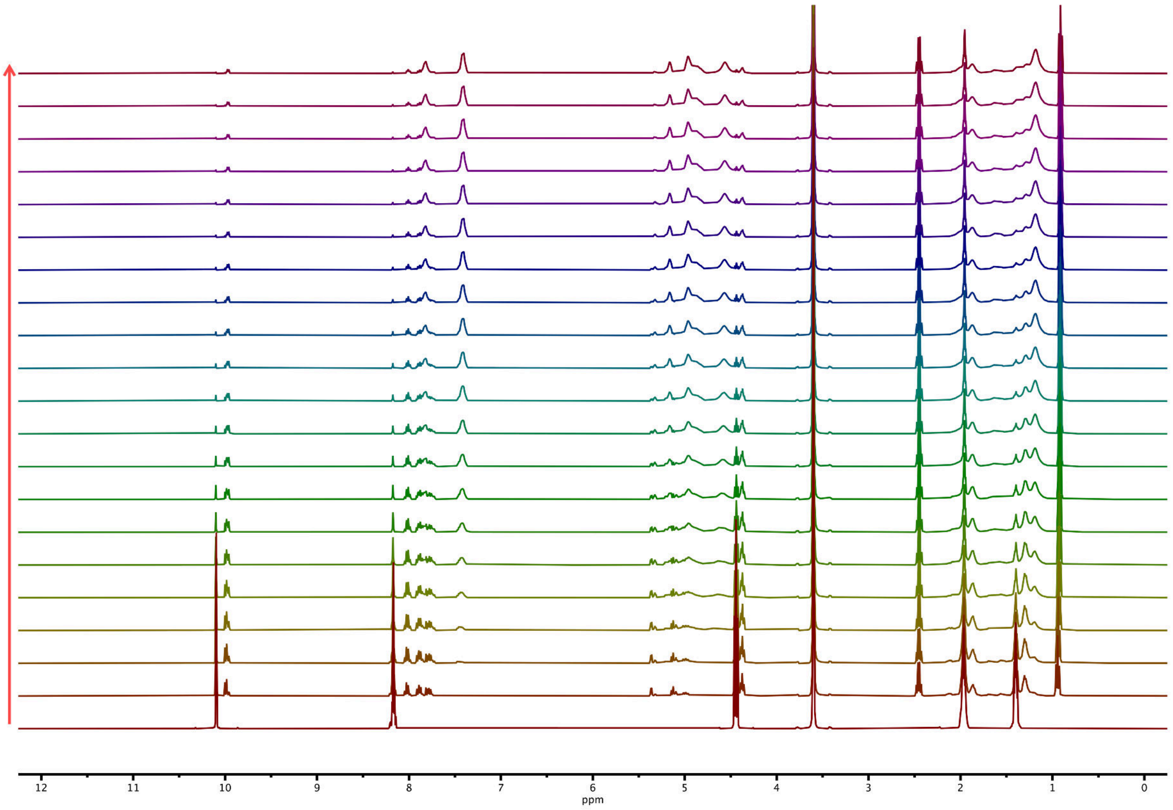

Figure S72: *[1]NMR spectra of dynamer* ***3d*** *with 15 mol% Et_3N (0 min-60 min; 0.5 M; CD_3CN).*

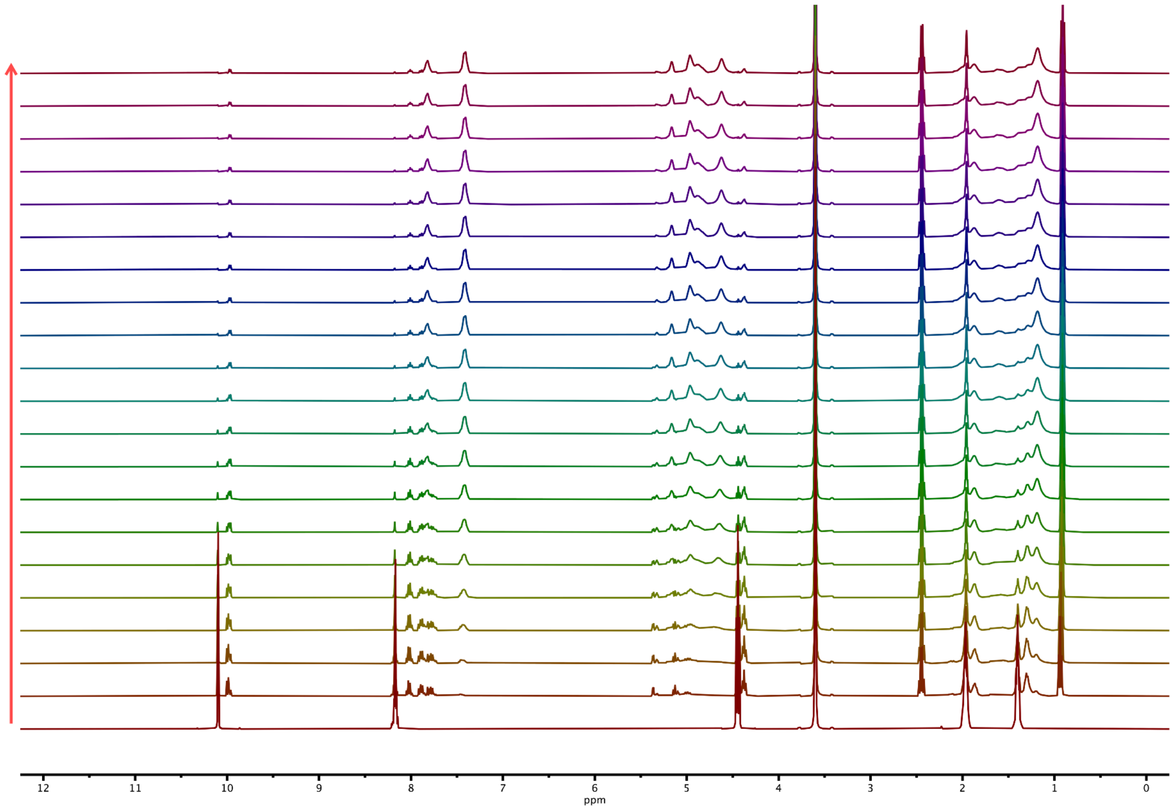

Figure S73: *[1]NMR spectra of dynamer* ***3d*** *with 20 mol% Et_3N (0 min-60 min; 0.5 M; CD_3CN).*

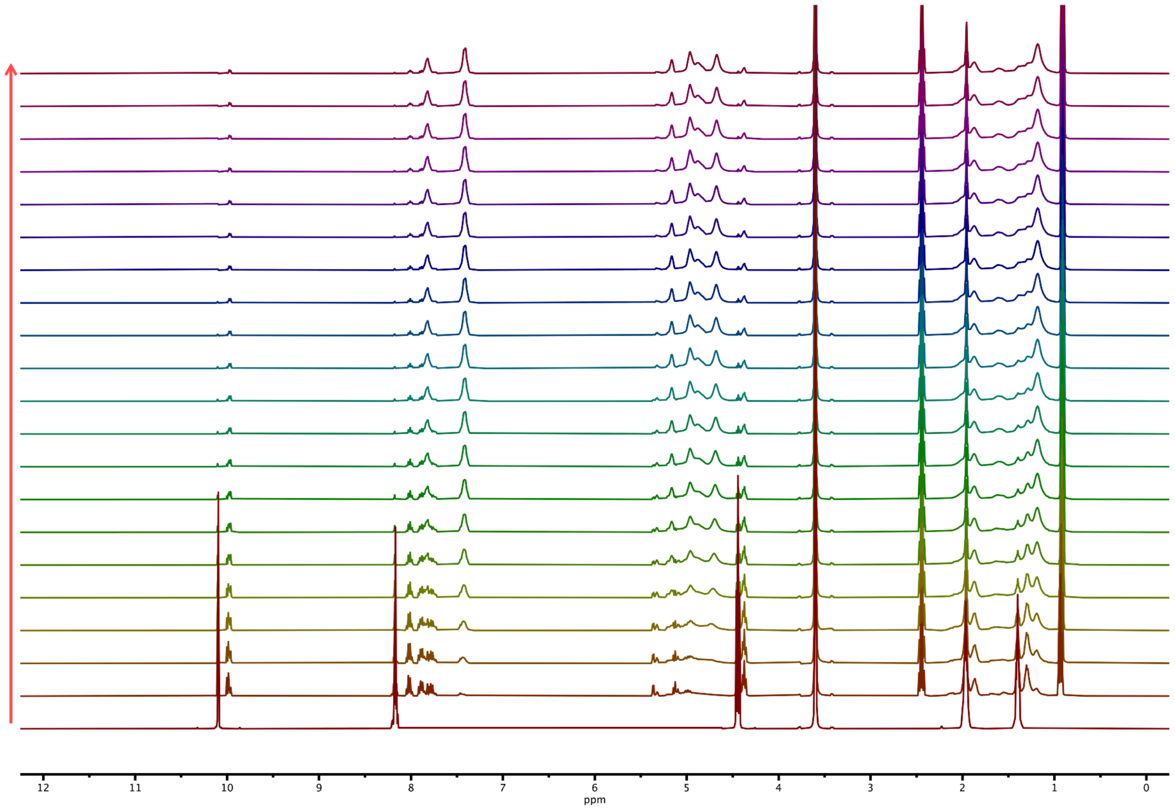

Figure S74: *[1]NMR spectra of dynamer* ***3d*** *with 25 mol% Et_3N (0 min-60 min; 0.5 M; CD_3CN).*

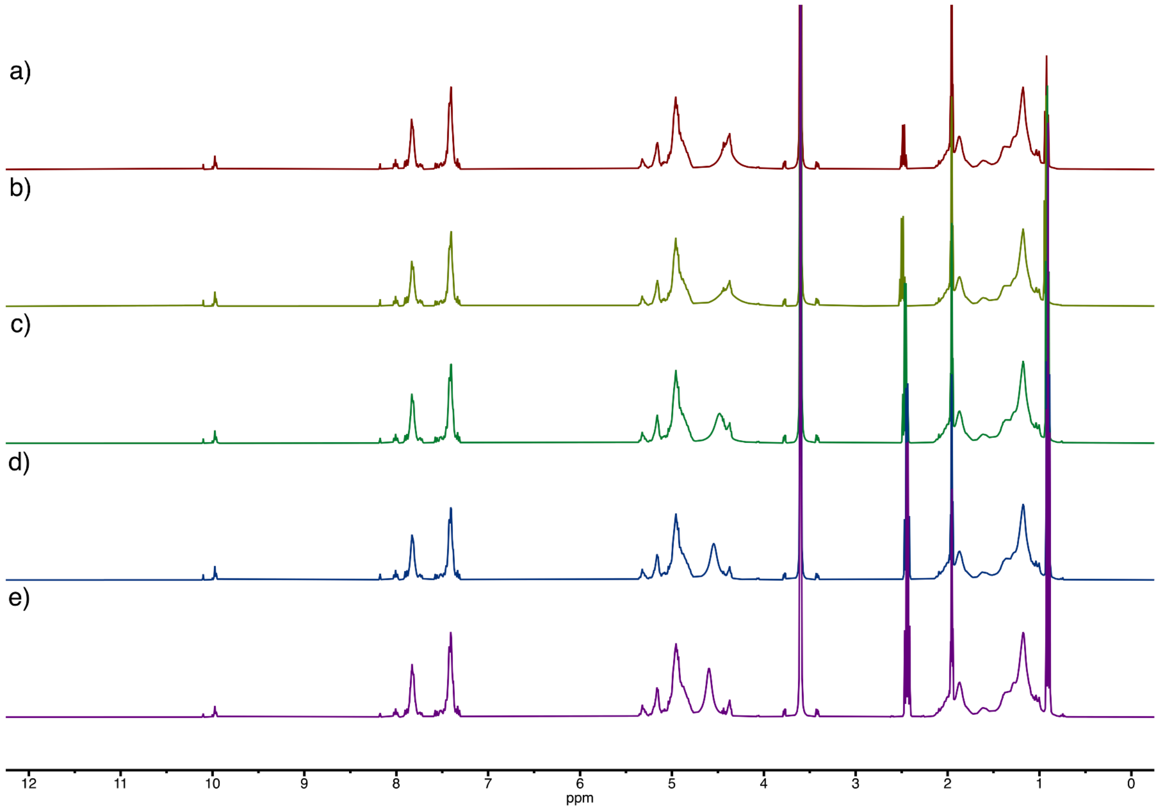

Figure S75: *^{1}NMR spectra of dynamer **3d** with a) 5 mol%; b) 10 mol%; c) 15 mol%; d) 20 mol%; e) 25 mol% Et_3N (after 12 h; 0.5 M; CD_3CN).*

Diffusivity

Table S3: *Effect of base on diffusion coefficient (D).*

**NEt_3 (mol%)**	***D* (cm^2/s)**
5	3.07×10^{-10}
10	2.78×10^{-10}
15	2.97×10^{-10}
20	2.89×10^{-11}
25	2.87×10^{-11}

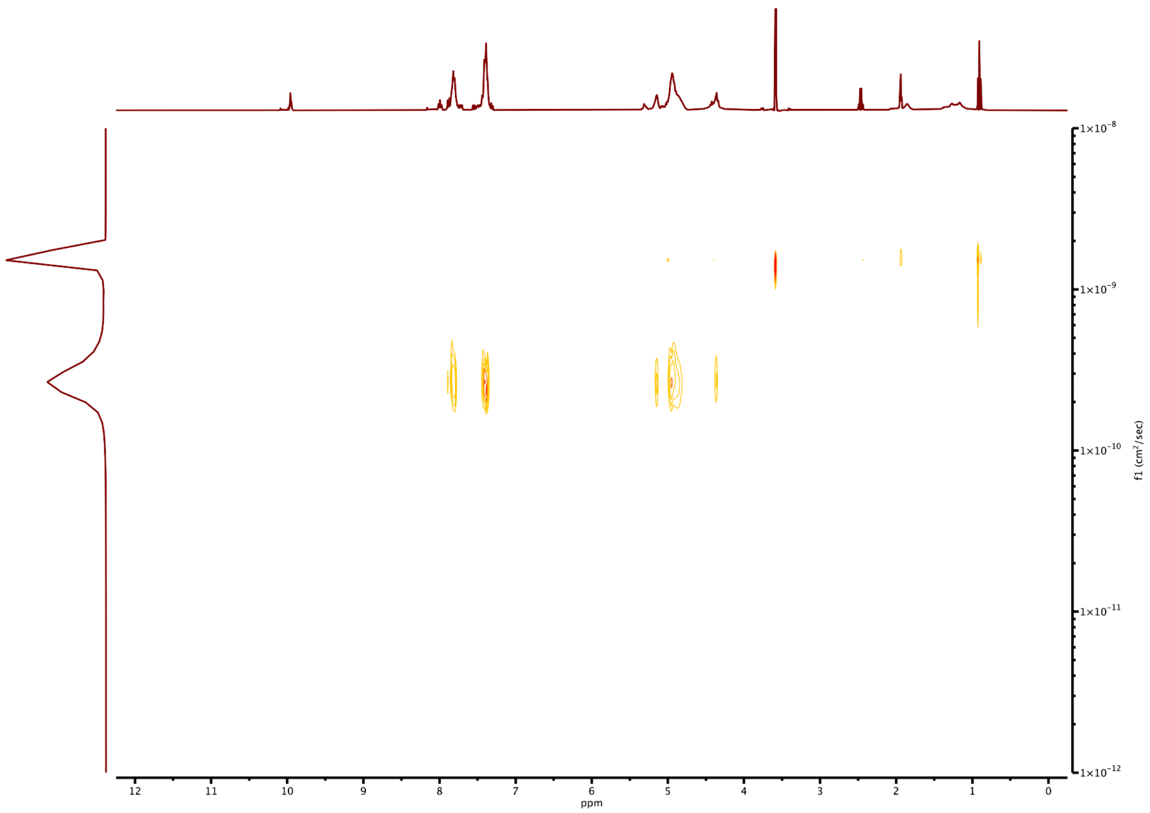

Figure S81: *Pseudo-2D DOSY plot of dynamer **3d** with 5 mol% Et_3N (after 12 h; 0.5 M; CD_3CN).*

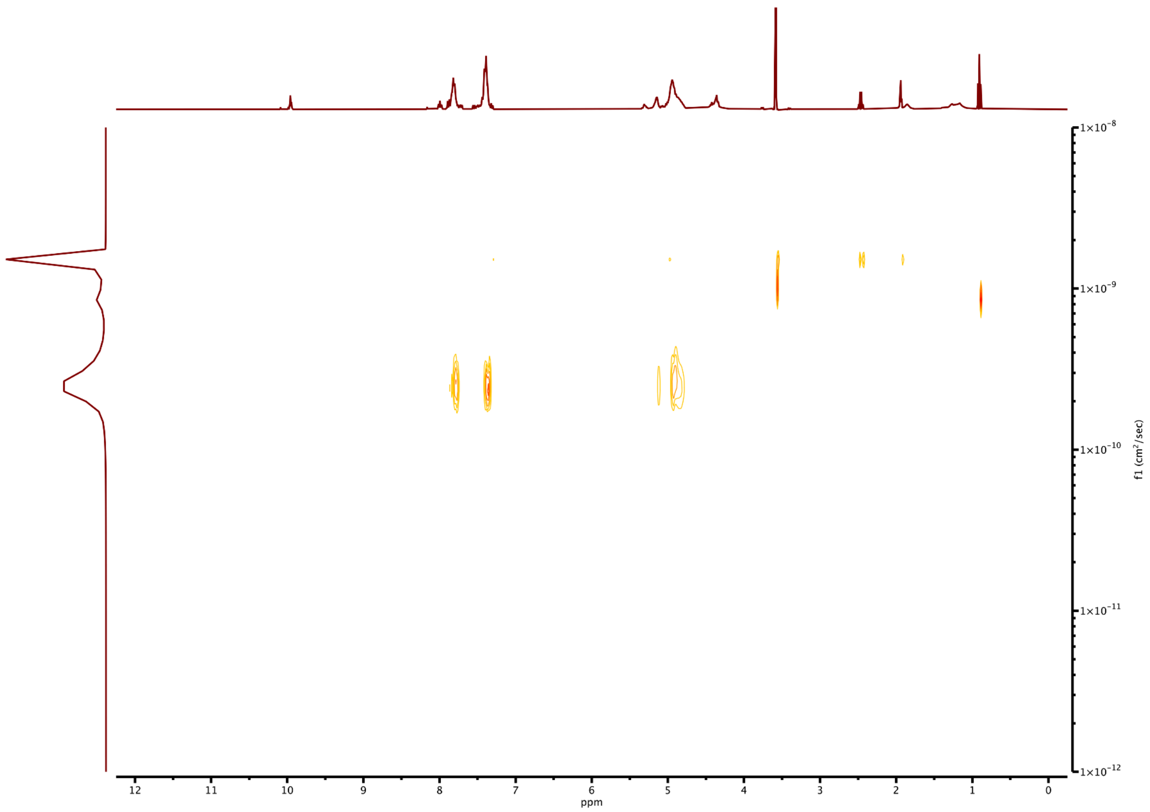

Figure S82: *Pseudo-2D DOSY plot of dynamer **3d** with 10 mol% Et_3N (after 12 h; 0.5 M; CD_3CN).*

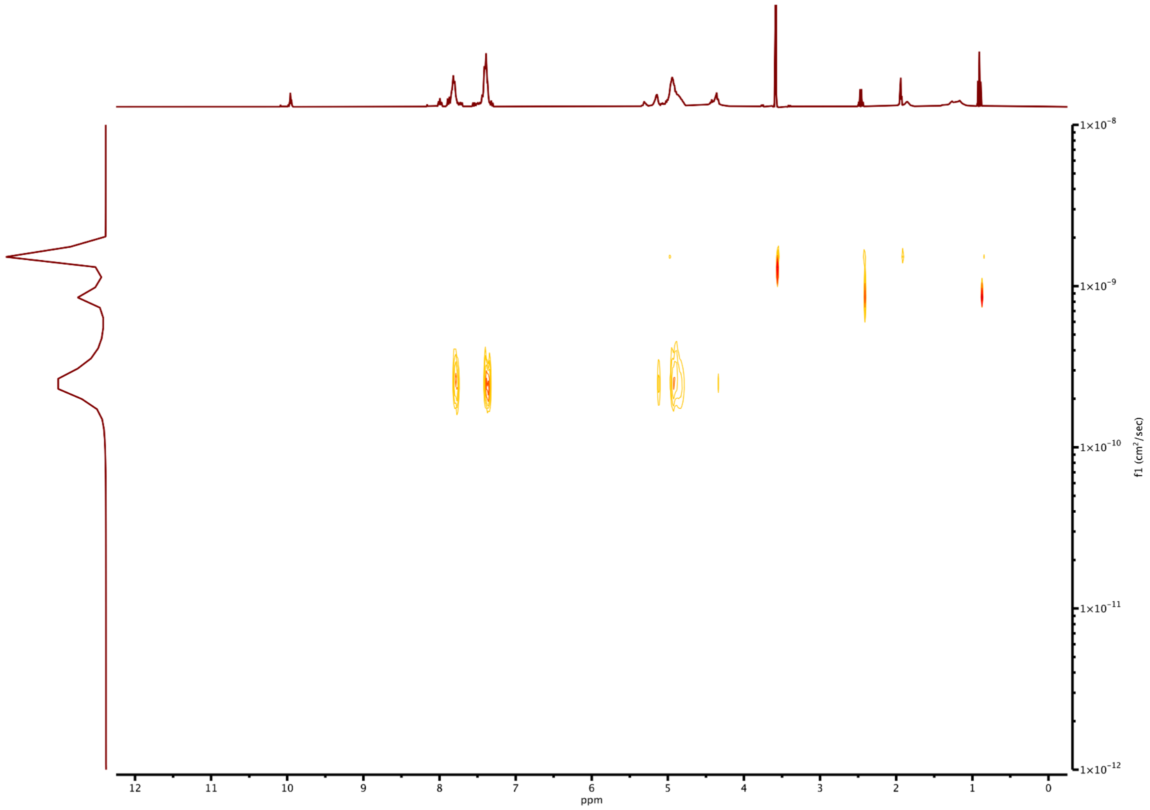

Figure S83: *Pseudo-2D DOSY plot of dynamer **3d** with 15 mol% Et_3N (after 12 h; 0.5 M; CD_3CN).*

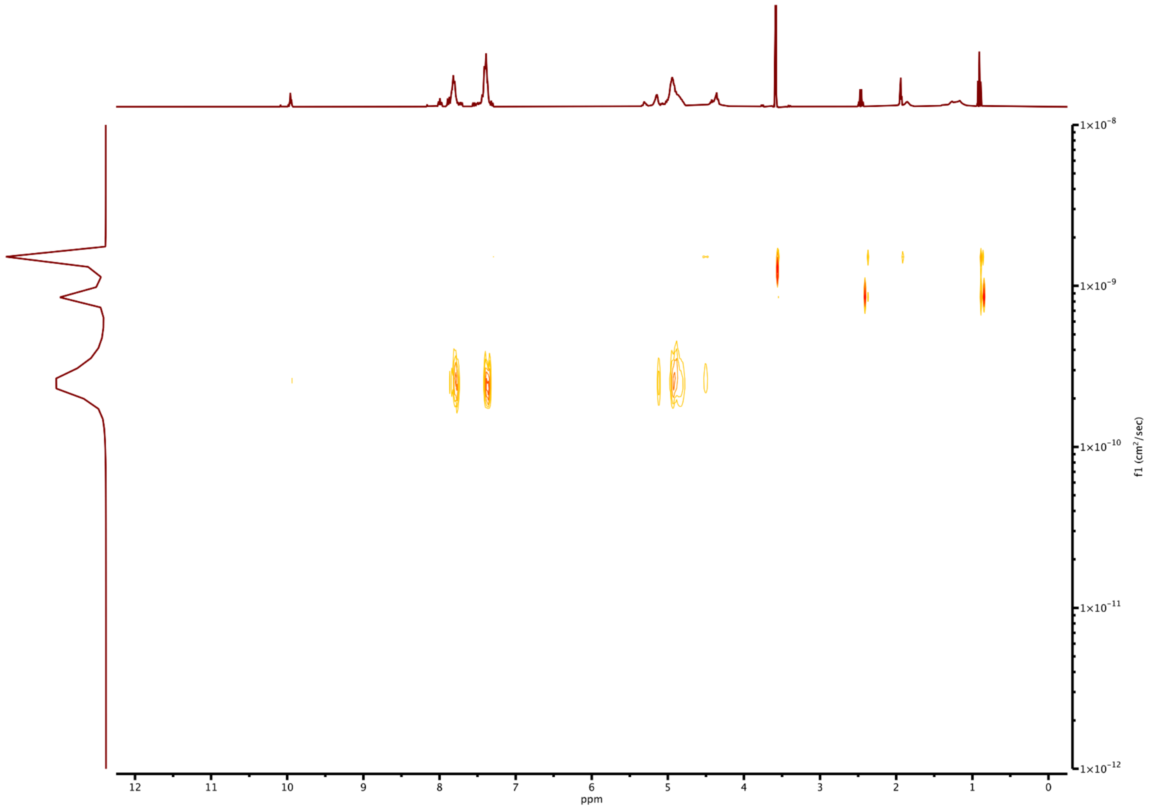

Figure S84: *Pseudo-2D DOSY plot of dynamer **3d** with 20 mol% Et_3N (after 12 h; 0.5 M; CD_3CN).*

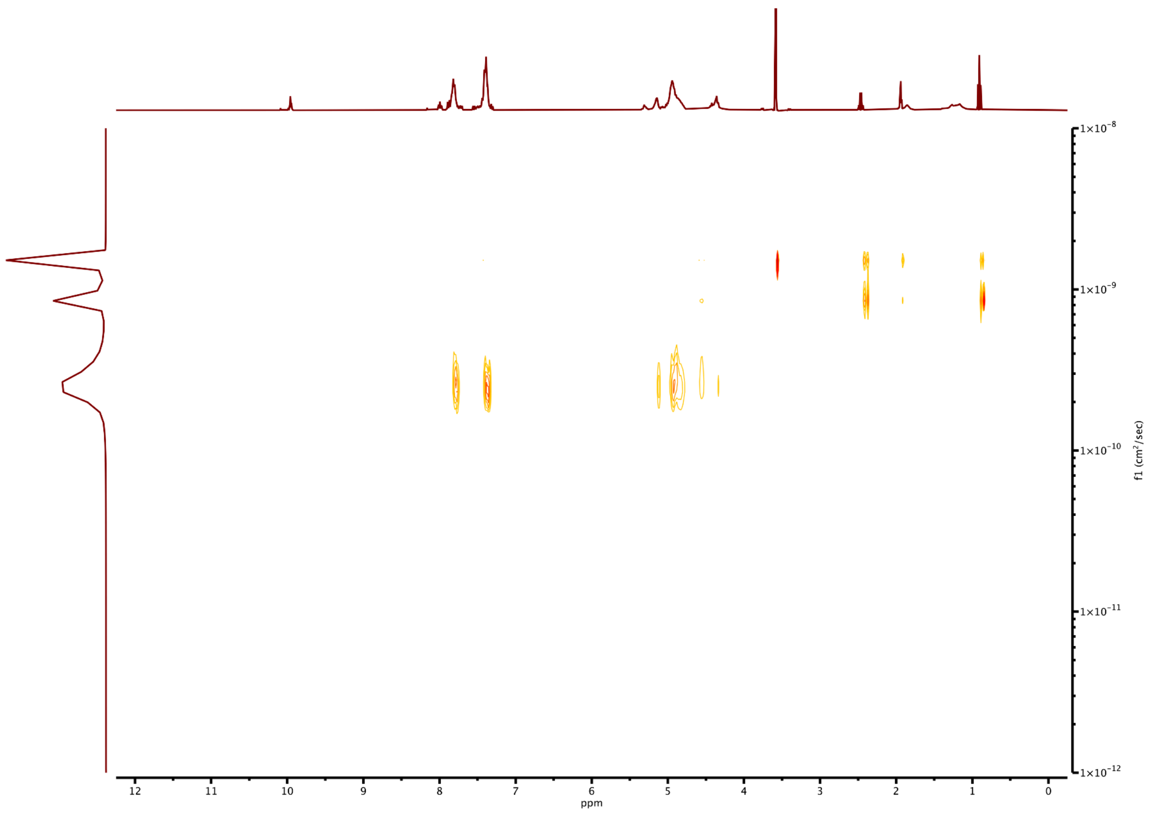

Figure S85: *Pseudo-2D DOSY plot of dynamer* ***3d*** *with 25 mol% Et_3N (after 12 h; 0.5 M; CD_3CN).*

Molecular weight

The solvent of the solutions of dynamer **3d** was replaced by THF and the samples analyzed by GPC.

Table S4: *Effect of base on molecular weight.*

NEt_3 (mol%)	M_n (g/mol)	M_w (g/mol)	M_z (g/mol)	*Đ*
5	7900	12800	19300	1.6
10	9400	16100	25400	1.7
15	9700	17200	28200	1.8
20	9500	16800	27400	1.8
25	10200	19400	33800	1.9

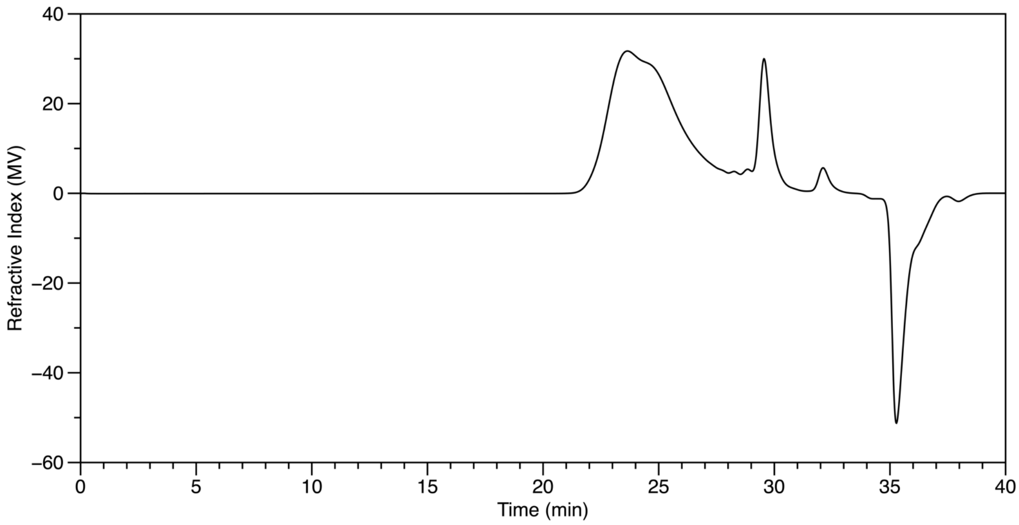

Figure S86: *GPC profile of dynamer* ***3d*** *with 5 mol% of Et_3N.*

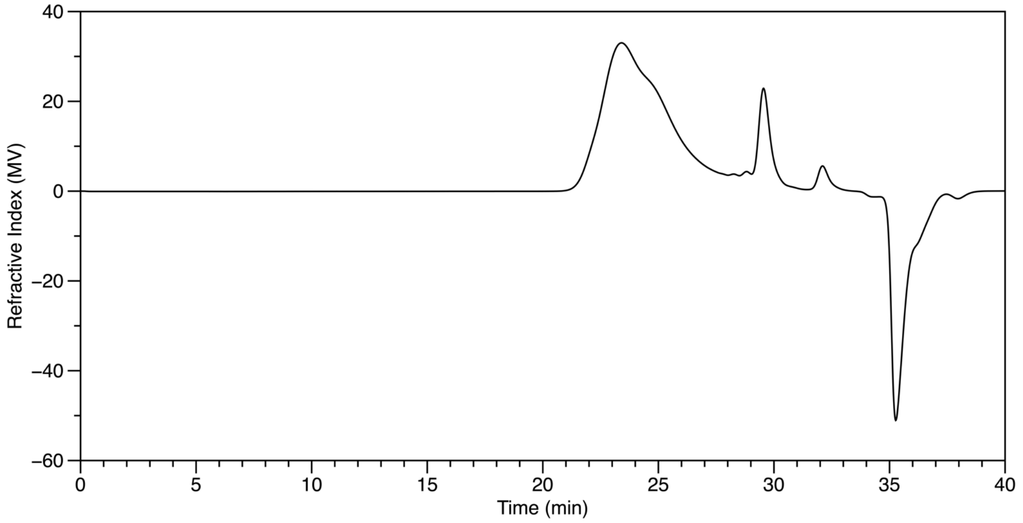

Figure S87: *GPC profile of dynamer* ***3d*** *with 10 mol% of* Et_3N.

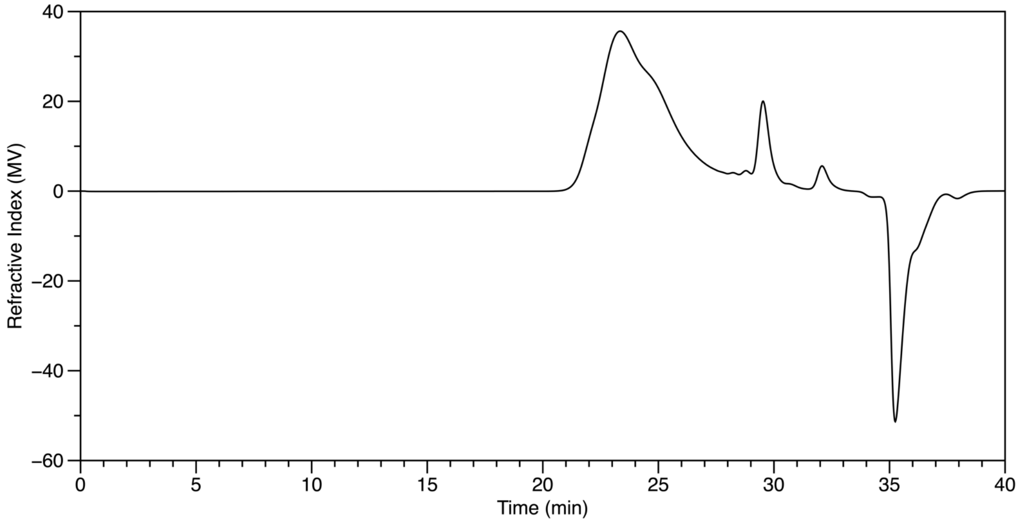

Figure S88: *GPC profile of dynamer* ***3d*** *with 15 mol% of* Et_3N.

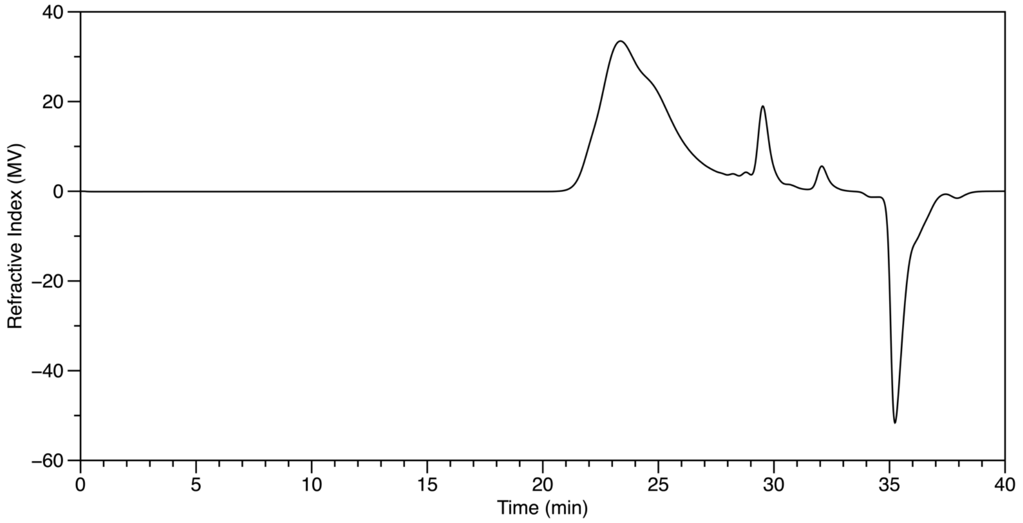

Figure S89: *GPC profile of dynamer* ***3d*** *with 20 mol% of* Et_3N.

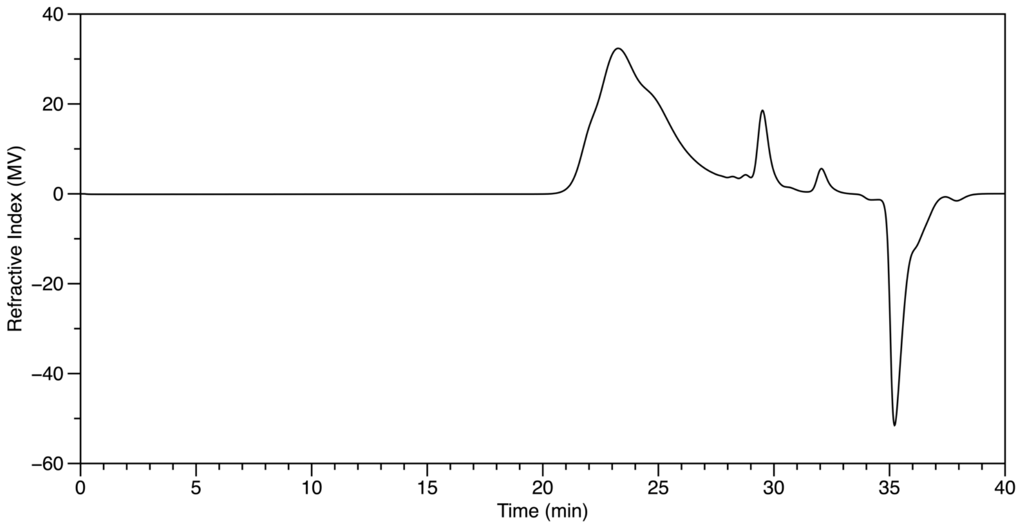

Figure S90: *GPC profile of dynamer **3d** with 25 mol% of Et_3N.*

Effects of feed concentration

Solutions of compounds **1** and **2d** at feed concentrations of 0.125 M–2.5 M (10 mol% Et_3N) in CD_3CN were analyzed by NMR.

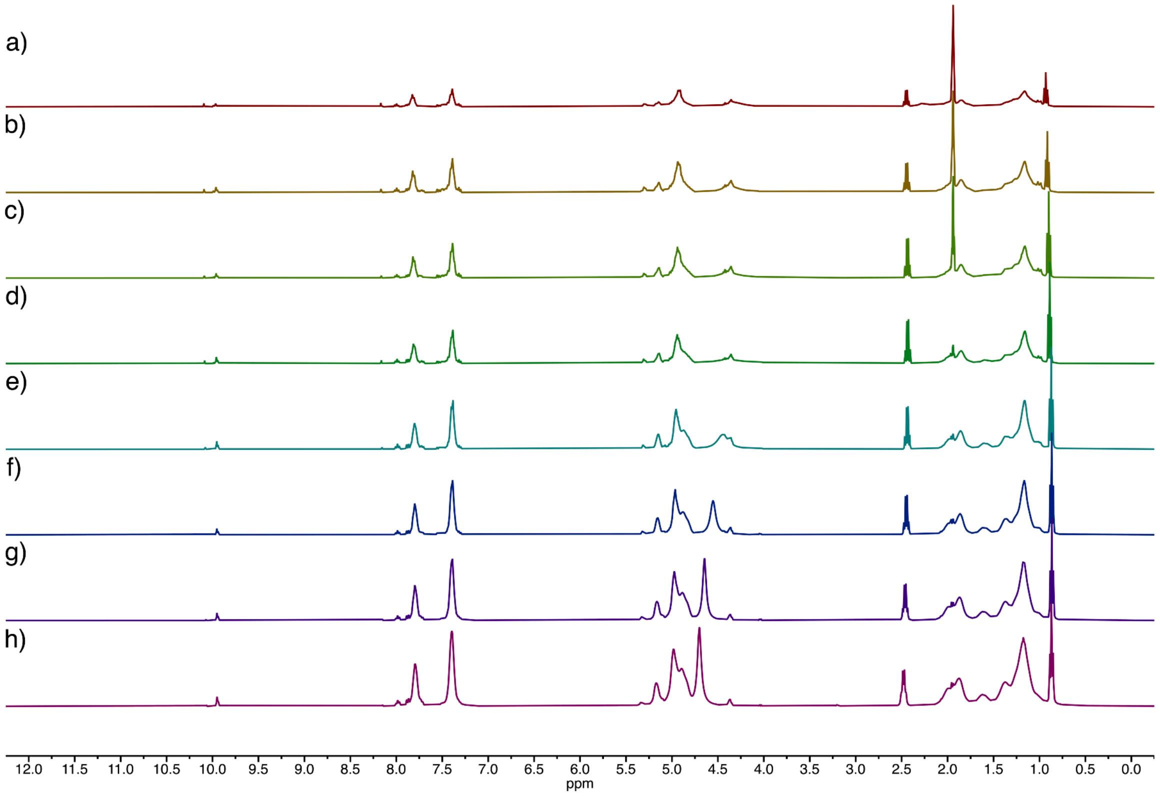

Figure S91: *[1]NMR spectra of dynamer **3a** with a) 0.125 M; b) 0.25 M; c) 0.375 M; d) 0.5 M; e) 1 M; f) 1.5 M; g) 2 M ; h) 2.5 M feed concentration (10 mol% Et_3N; 400 MHz; CD_3CN).*

Diffusivity

Table S5: *Effect of feed concentration on diffusion coefficient (D).*

Feed concentration (M)	*D* (cm²/s)
0.125	5.5×10^{-10}
0.25	5.5×10^{-10}
0.375	3.6×10^{-10}
0.50	2.3×10^{-10}
1.0	8.3×10^{-11}
1.5	3.5×10^{-11}
2.0	1.7×10^{-11}
2.5	1.1×10^{-11}

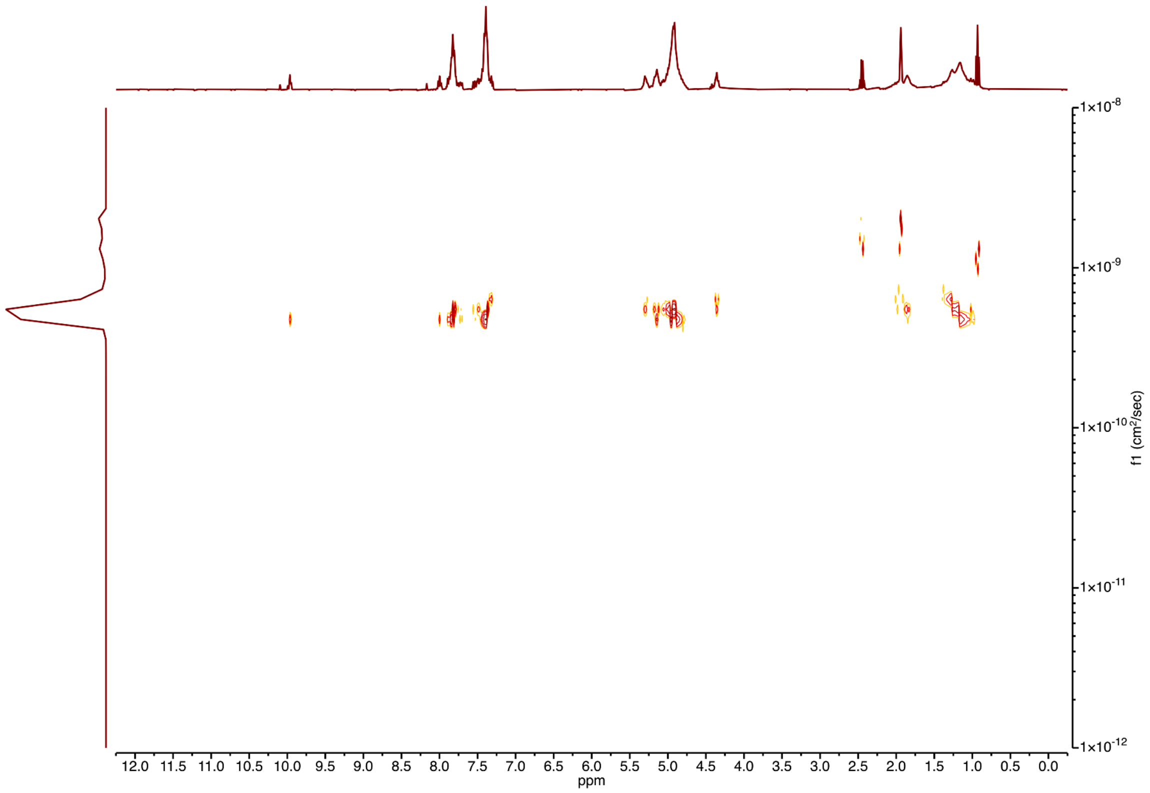

Figure S92: *Pseudo-2D DOSY plot of dynamer **3d** (0.125 M, 10 mol% of Et_3N; CD_3CN).*

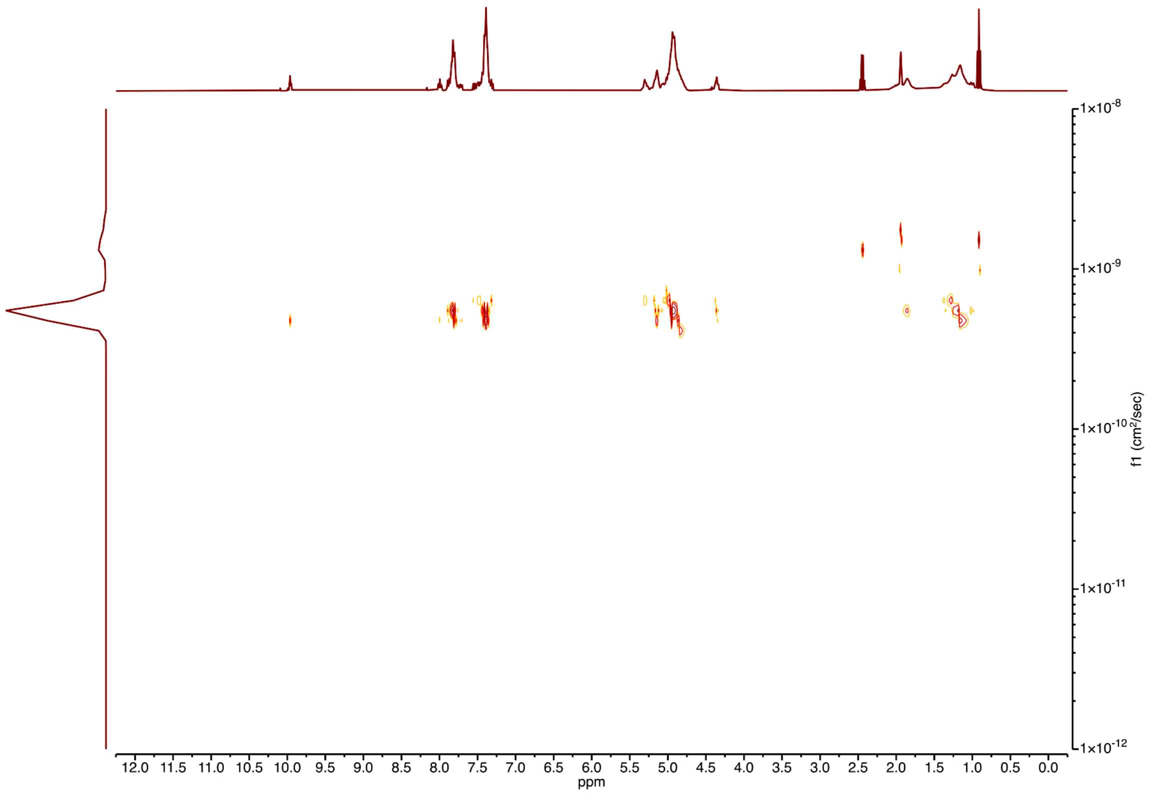

Figure S93: *Pseudo-2D DOSY plot of dynamer* ***3d*** *(0.25 M, 10 mol% of Et_3N; CD_3CN).*

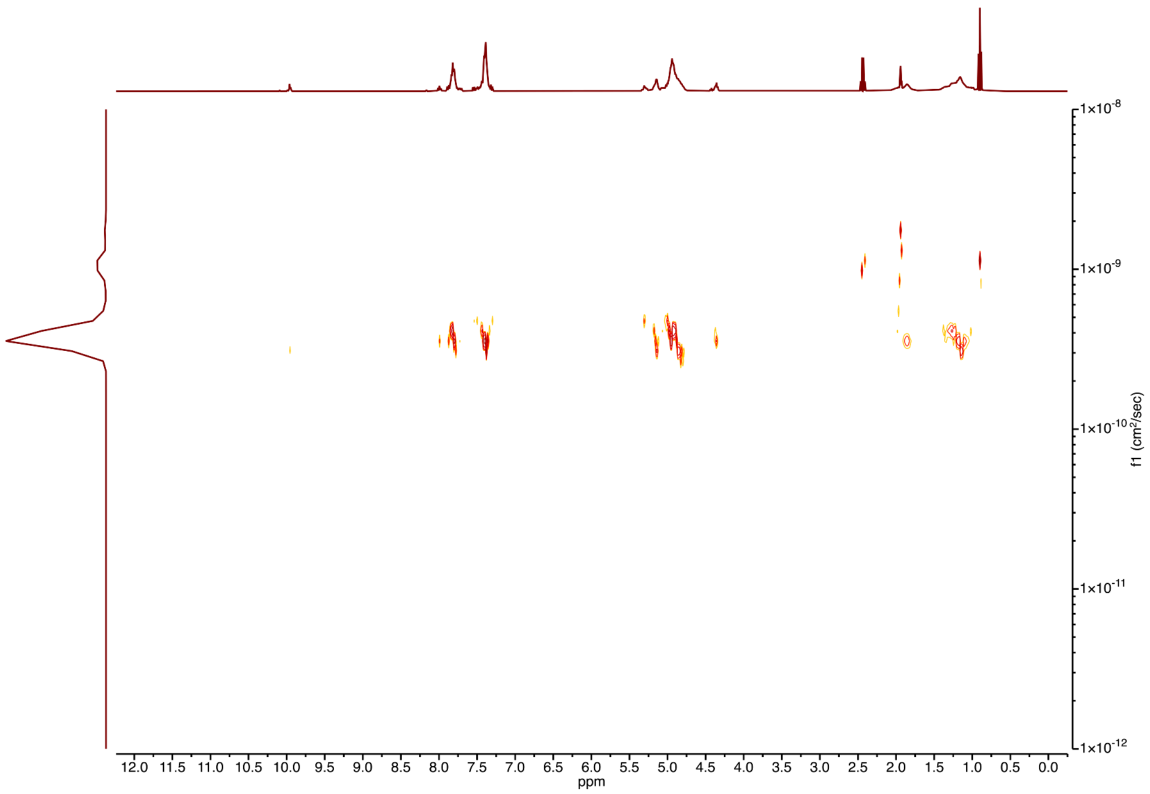

Figure S94: *Pseudo-2D DOSY plot of dynamer* ***3d*** *(0.375 M, 10 mol% of Et_3N; CD_3CN).*

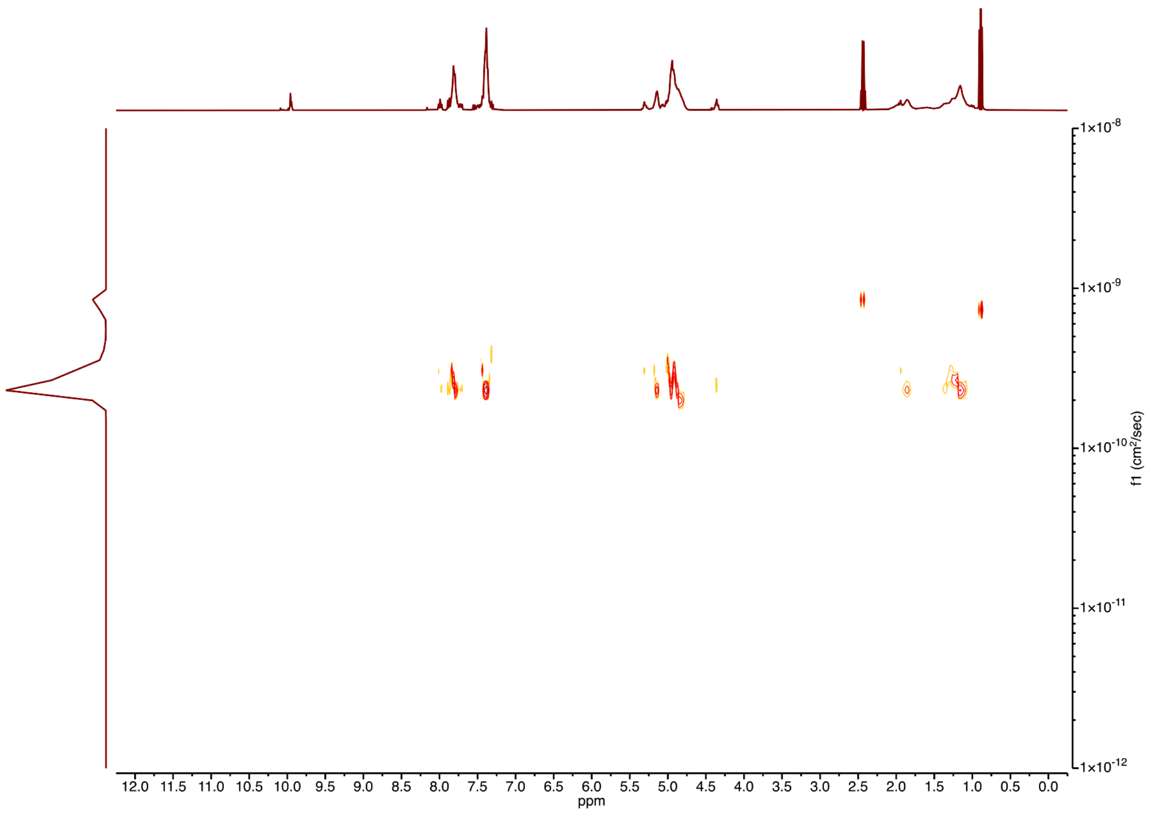

Figure S95: *Pseudo-2D DOSY plot of dynamer* ***3d*** *(0.5 M, 10 mol% of Et_3N; CD_3CN).*

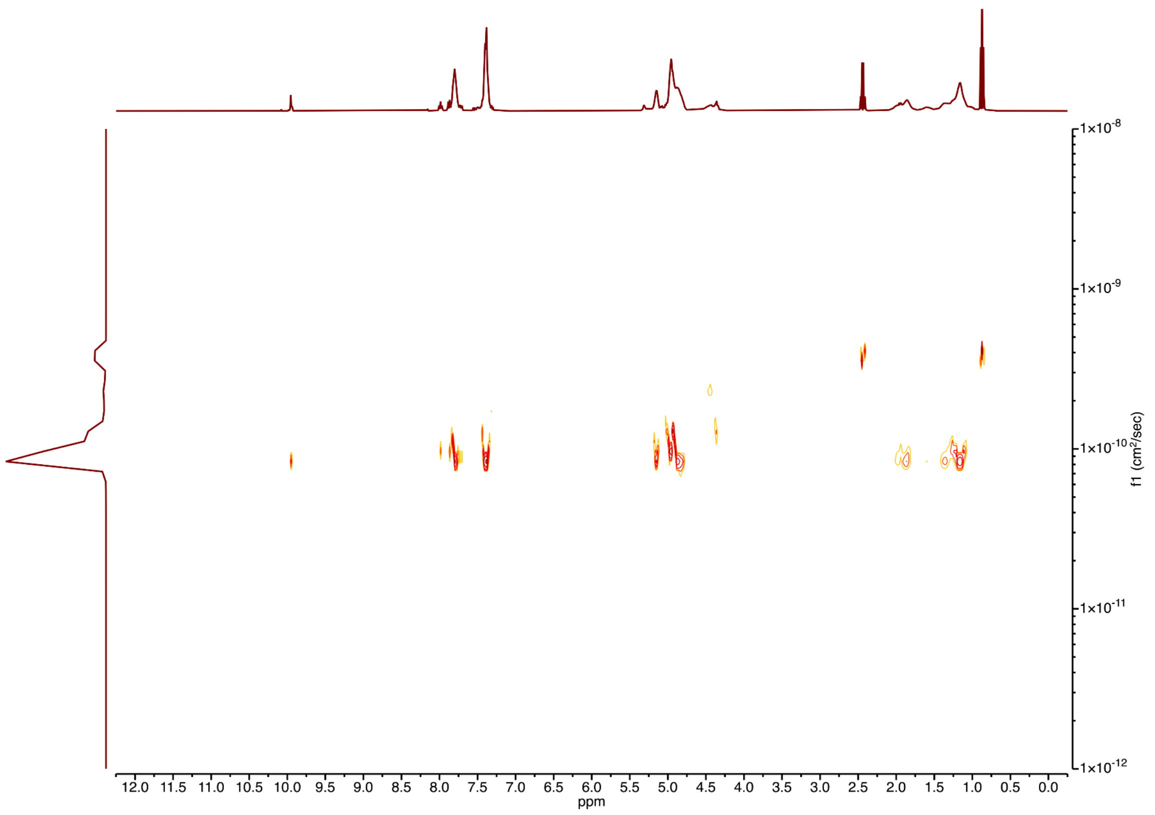

Figure S96: *Pseudo-2D DOSY plot of dynamer* ***3d*** *(1 M, 10 mol% of Et_3N; CD_3CN).*

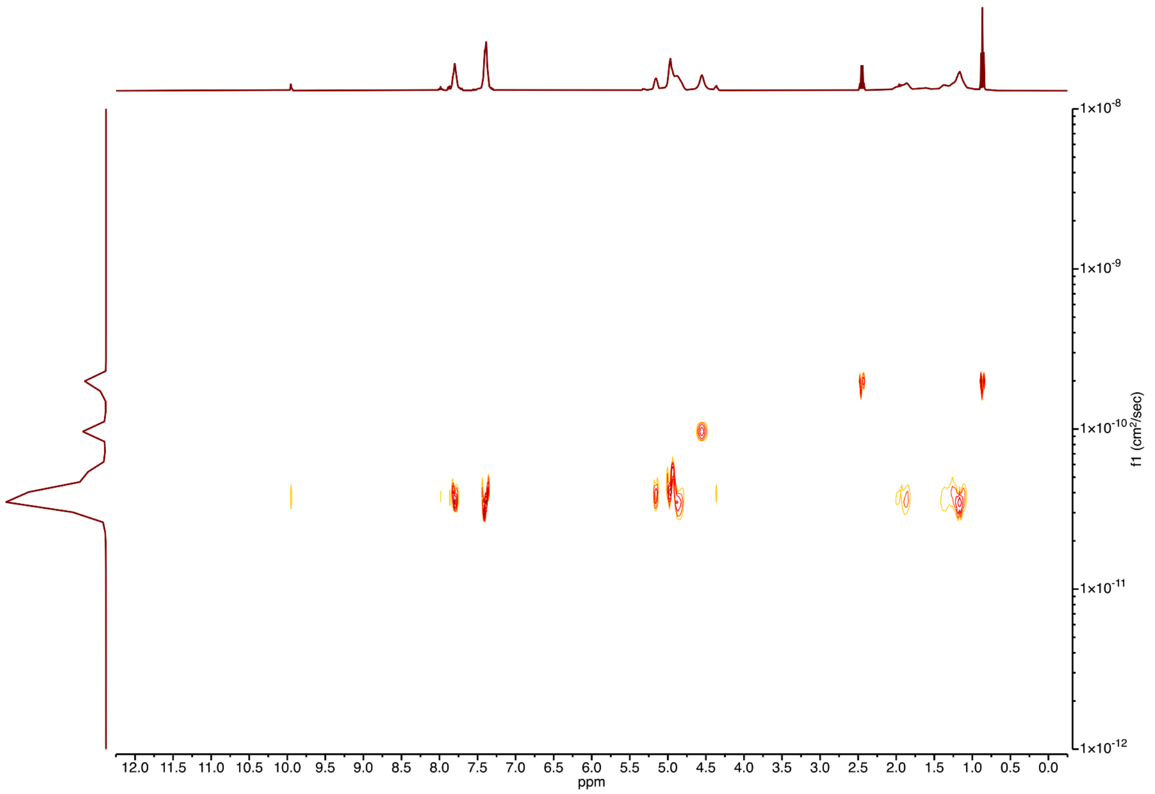

Figure S97: *Pseudo-2D DOSY plot of dynamer* ***3d*** *(1.5 M, 10 mol% of Et_3N; CD_3CN).*

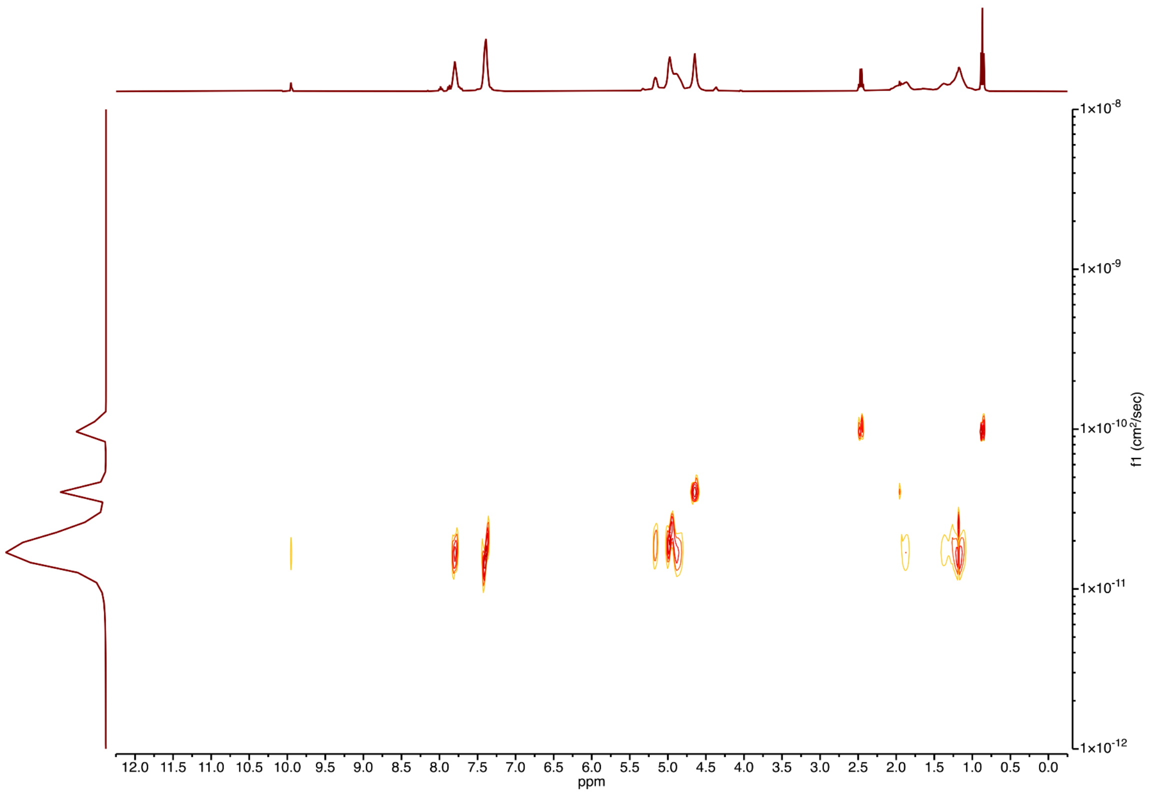

Figure S98: *Pseudo-2D DOSY plot of dynamer* ***3d*** *(2 M, 10 mol% of Et_3N; CD_3CN).*

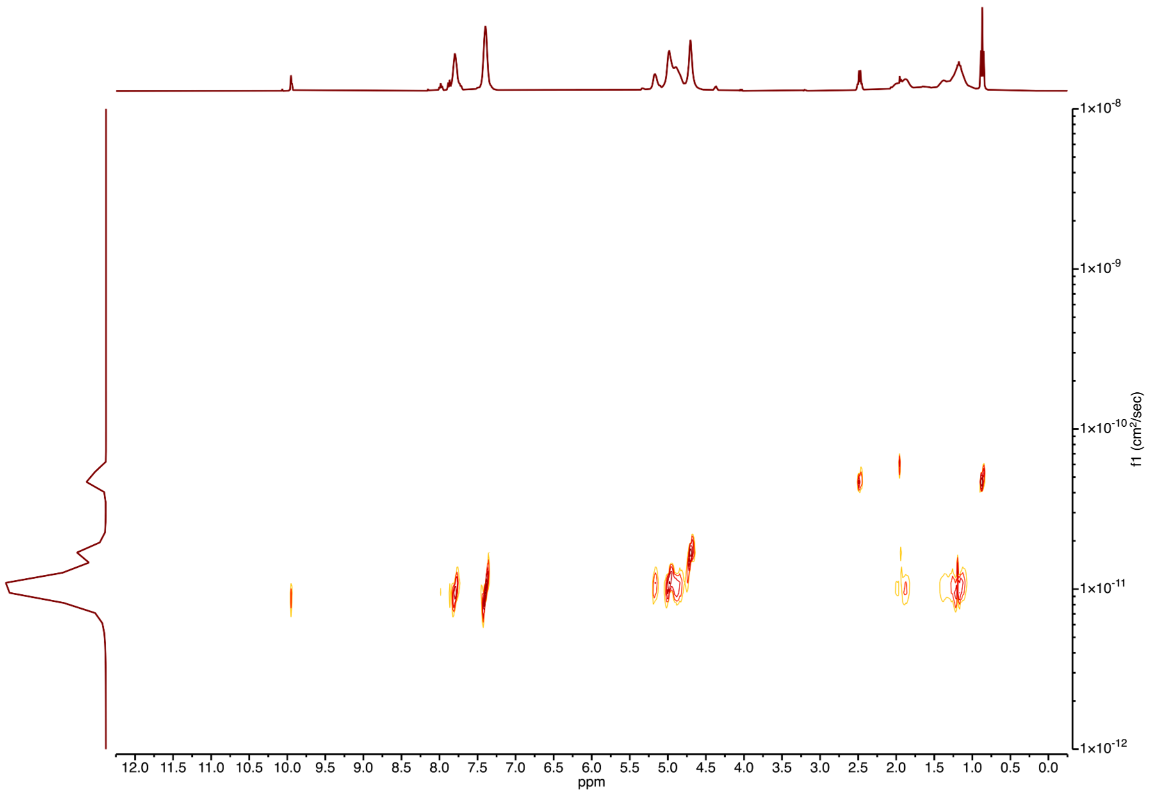

Figure S99: *Pseudo-2D DOSY plot of dynamer **3d** (2.5 M, 10 mol% of Et_3N; CD_3CN).*

Molecular weight

The solvent of the solutions of dynamer **3d** was replaced by THF and the samples analyzed by GPC.

Table S6: *Effect of feed concentration on molecular weight.*

Feed concentration (M)	M_n (g/mol)	M_w (g/mol)	M_z (g/mol)	*Đ*
0.125	2800	2900	3100	1.0
0.25	6500	8600	11700	1.3
0.375	7200	10600	15400	1.5
0.50	9600	14800	22200	1.5
1.0	10100	16600	26400	1.6
1.5	10400	17500	28000	1.7
2.0	9000	15400	25000	1.7
2.5	8800	15100	24800	1.7

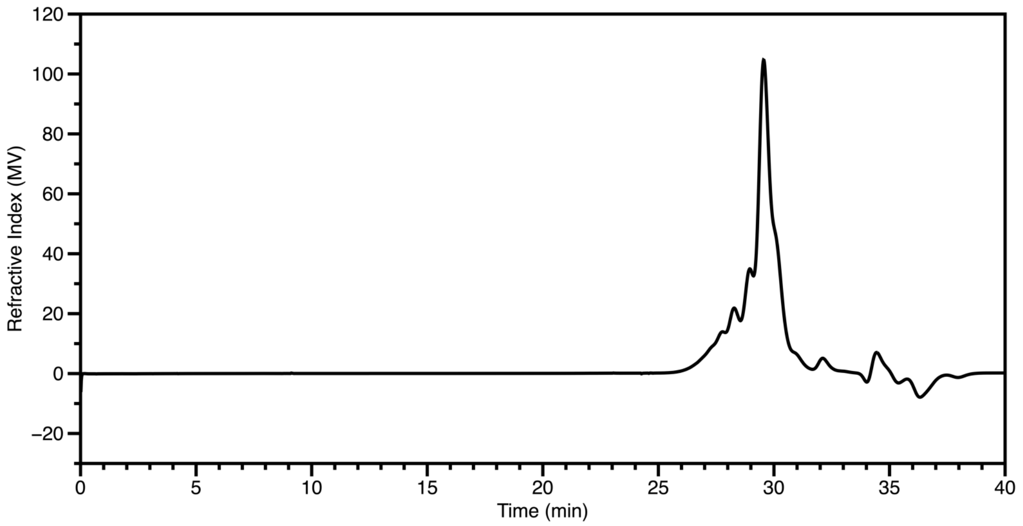

Figure S100: *GPC profile of dynamer **3d** (0.125 M, 10 mol% Et_3N).*

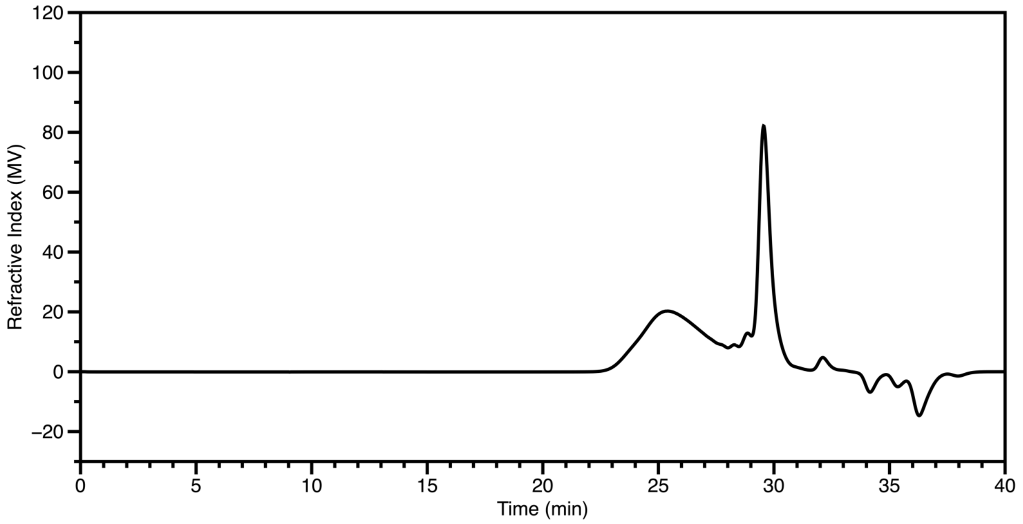

Figure S101: *GPC profile of dynamer **3d** (0.25 M, 10 mol% Et_3N).*

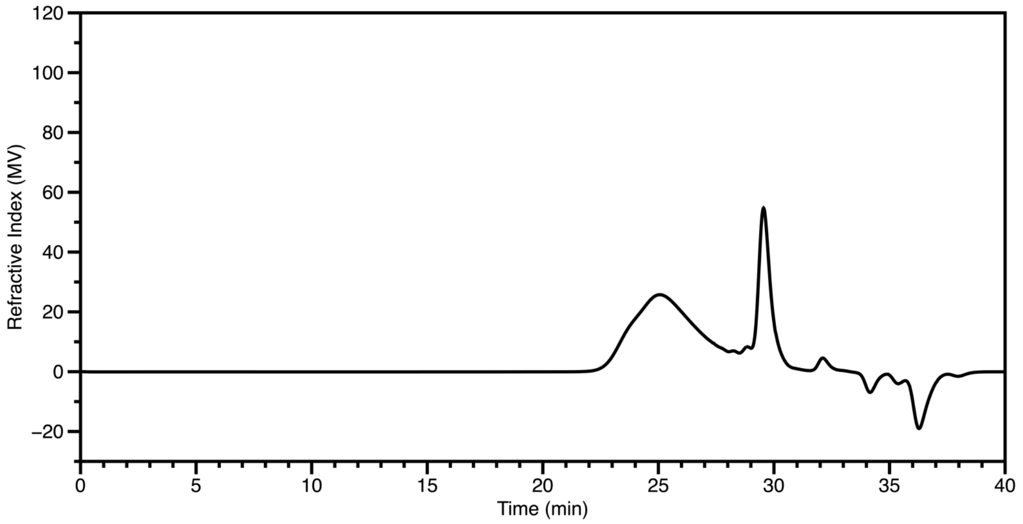

Figure S102: *GPC profile of dynamer **3d** (0.375 M, 10 mol% Et_3N).*

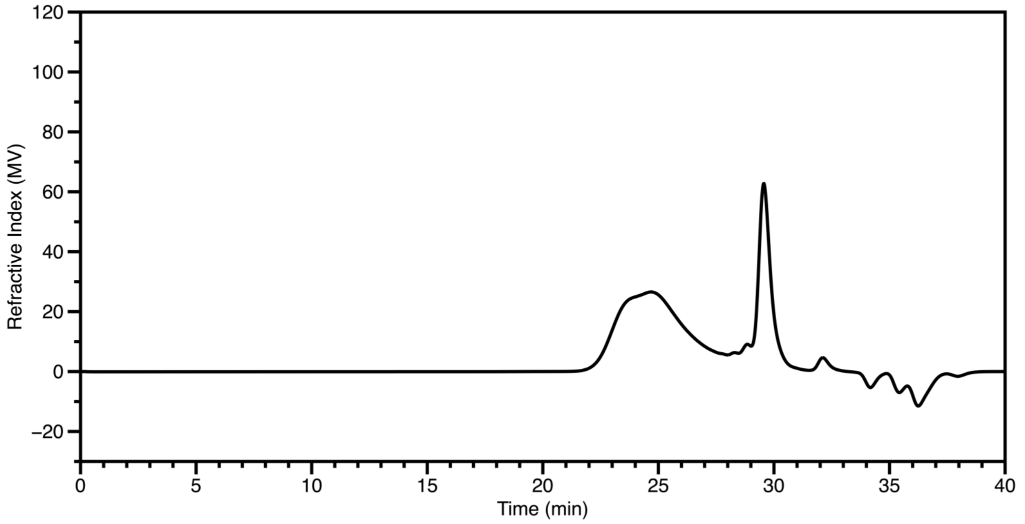

Figure S103: *GPC profile of dynamer* ***3d*** *(0.5 M, 10 mol% Et_3N).*

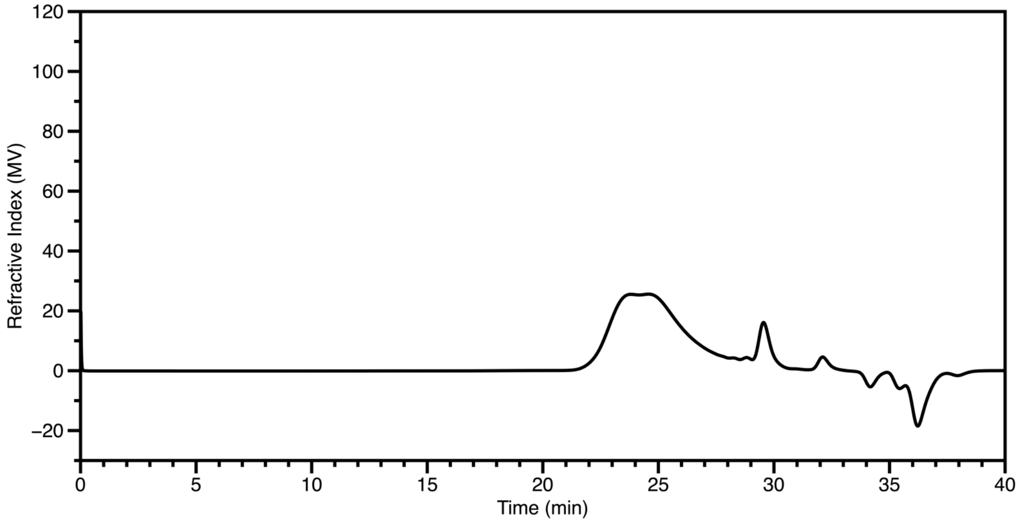

Figure S104: *GPC profile of dynamer* ***3d*** *(1 M, 10 mol% Et_3N).*

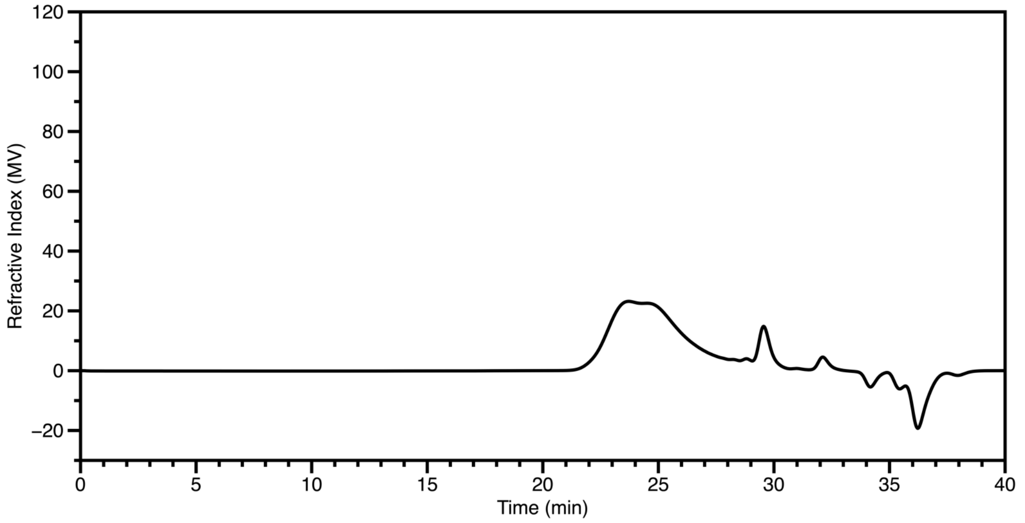

Figure S105: *GPC profile of dynamer* ***3d*** *(1.5 M, 10 mol% Et_3N).*

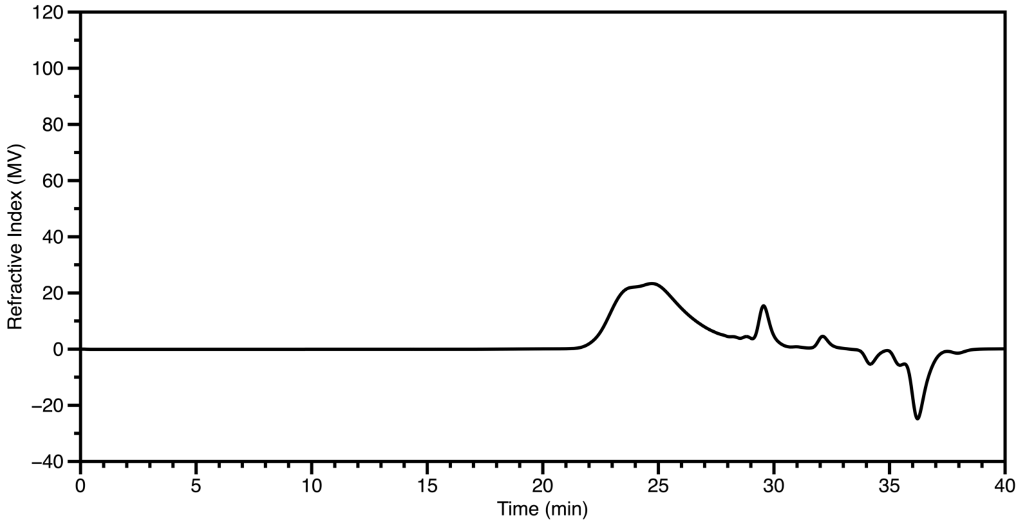

Figure S106: *GPC profile of dynamer **3d** (2 M, 10 mol% Et_3N).*

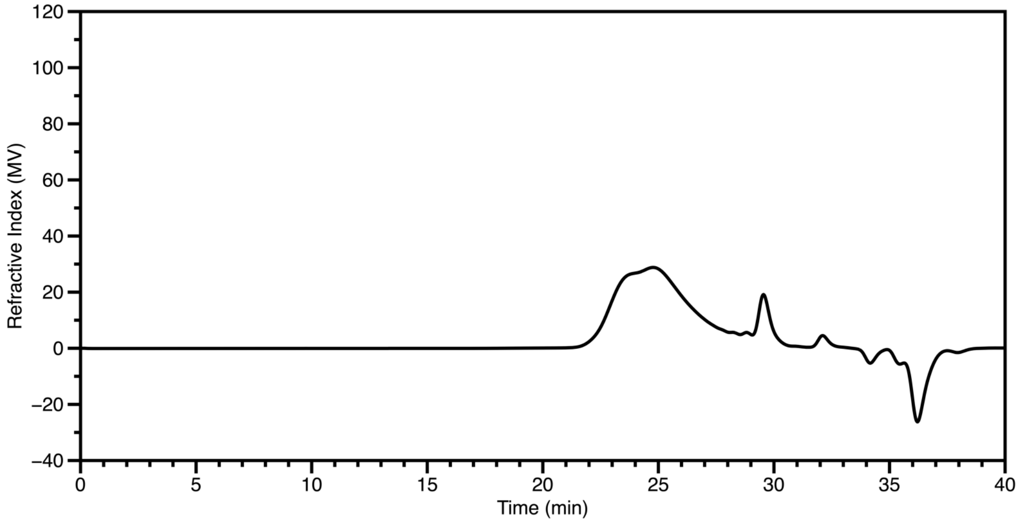

Figure S107: *GPC profile of dynamer **3d** (2.5 M, 10 mol% Et_3N).*

System dynamics - stimuli-responsiveness

Solutions of dynamer **3d** were made from compounds **1** and **2d** in CD_3CN (0.5 M, 10 mol% Et_3N). To these, one equivalent of compounds **4**, **5**, or **2b** was added, respectively. After 24 h, the samples were analyzed by DOSY NMR, after which he solvent was replaced by THF and the solutions analyzed by GPC.

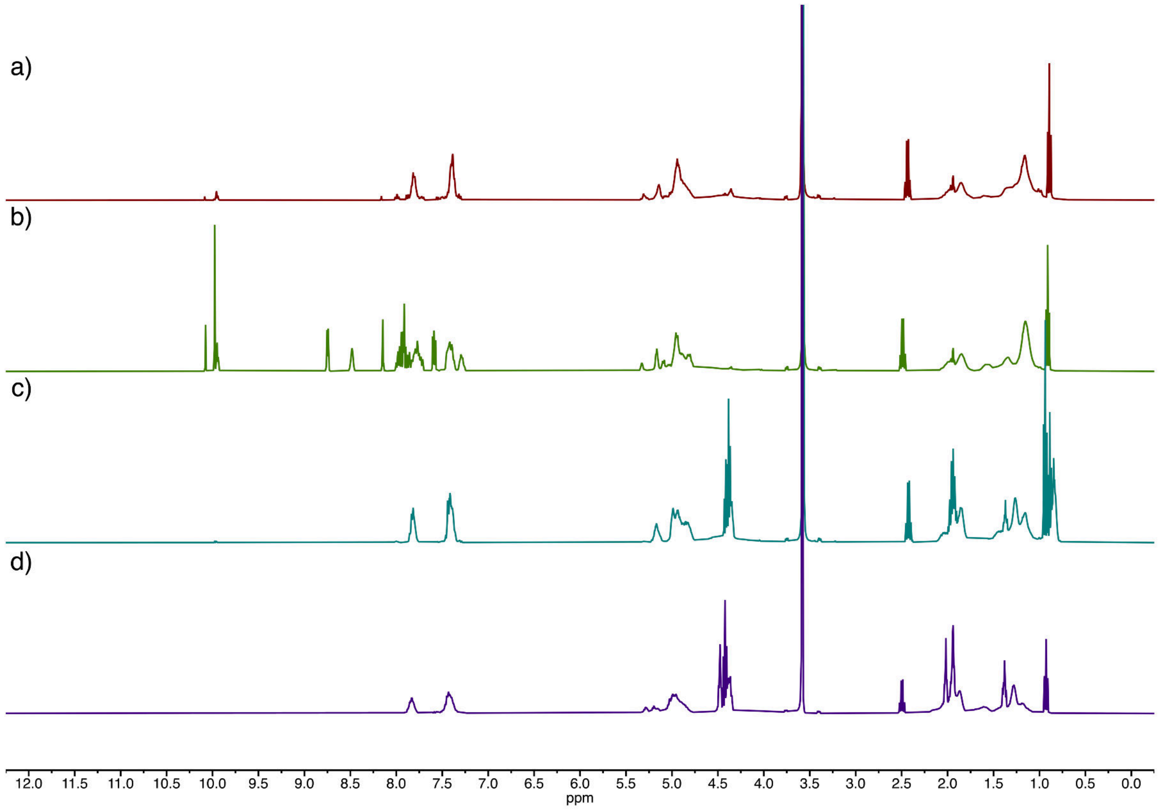

Figure S108: *^{1}H NMR spectrum of a) compound **3d**; b) compounds **3d+4**; c) compounds **3d+5**; d) compounds **3d+2b** (0.5 M, 10 mol% Et_3N; CD_3CN).*

Table S7: *Stimuli-responsive effects on diffusion coefficient (D) of dynamer **3d**.*

Sample	*D* (cm²/s)
**3d**	2.60×10^{-10}
**3d+4**	3.06×10^{-10}
**3d+5**	4.06×10^{-10}
**3d+2b**	3.59×10^{-10}

Table S8: *Stimuli-responsive effects on molecular weight of dynamer **3d**.*

Sample	M_n (g/mol)	M_w (g/mol)	M_z (g/mol)	*Đ*
**3d**	9600	14800	22200	1.5
**3d+4**	2800	3000	3300	1.1
**3d+5**	2300	2500	2800	1.1
**3d+2b**	1800	1800	1900	1.1

References

(1) Abebayehu, A.; Dutta, R.; Lee, C.-H. *Chem. Eur. J.* **2016**, *22* (39), 13850–13856.
(2) Ballini, R.; Barboni, L.; Giarlo, G. *J. Org. Chem.* **2004**, *69* (20), 6907–6908.
(3) Schulz, M.; Wimmer, K.; Görls, H.; Westerhausen, M. *Z. Naturforsch. B* **2011**, *66* (6), 611–623.
